# Exploring pyrrolidinyl-spirooxindole natural products as promising platforms for the synthesis of novel spirooxindoles as EGFR/CDK2 inhibitors for halting breast cancer cells

**DOI:** 10.3389/fchem.2024.1364378

**Published:** 2024-02-29

**Authors:** Mohamed S. Nafie, Abdullah Mohammed Al-Majid, M. Ali, Abdulmajeed Abdullah Alayyaf, Matti Haukka, Sajda Ashraf, Zaheer Ul-Haq, Ayman El-Faham, Assem Barakat

**Affiliations:** ^1^ Department of Chemistry, College of Sciences, University of Sharjah, Sharjah, United Arab Emirates; ^2^ Chemistry Department, Faculty of Science, Suez Canal University, Ismailia, Egypt; ^3^ Department of Chemistry, College of Science, King Saud University, Riyadh, Saudi Arabia; ^4^ Department of Chemistry, University of Jyväskylä, Jyväskylä, Finland; ^5^ Dr. Panjwani Center for Molecular medicine and Drug Research, International Center for Chemical and Biological Sciences, University of Karachi, Karachi, Pakistan; ^6^ Department of Chemistry, Faculty of Science, Alexandria University, Alexandria, Egypt

**Keywords:** spirooxindole, [3+2] cycloaddition, breast cancer (MCF-7 and MDA-MB-231), EGFR, CDK-2, molecular dynamics

## Abstract

Cancer represents a global challenge, and the pursuit of developing new cancer treatments that are potent, safe, less prone to drug resistance, and associated with fewer side effects poses a significant challenge in cancer research and drug discovery. Drawing inspiration from pyrrolidinyl-spirooxindole natural products, a novel series of spirooxindoles has been synthesized through a one-pot three-component reaction, involving a [3 + 2] cycloaddition reaction. The cytotoxicity against breast cancer cells (MCF-7 and MDA-MB-231) and safety profile against WISH cells of the newly developed library were assessed using the MTT assay. Compounds **5l** and **5o** exhibited notable cytotoxicity against MCF-7 cells (IC_50_ = 3.4 and 4.12 μM, respectively) and MDA-MB-231 cells (IC_50_ = 8.45 and 4.32 μM, respectively) compared to Erlotinib. Conversely, compounds **5a-f** displayed promising cytotoxicity against MCF-7 cells with IC_50_ values range (IC_50_ = 5.87–18.5 μM) with selective activity against MDA-MB-231 cancer cells. Compound **5g** demonstrated the highest cytotoxicity (IC_50_ = 2.8 μM) among the tested compounds. Additionally, compounds **5g**, **5l**, and **5n** were found to be safe (non-cytotoxic) against WISH cells with higher IC_50_ values ranging from 39.33 to 47.2 μM. Compounds **5g**, **5l**, and **5n** underwent testing for their inhibitory effects against EGFR and CDK-2. Remarkably, they demonstrated potent EGFR inhibition, with IC_50_ values of 0.026, 0.067, and 0.04 μM and inhibition percentages of 92.6%, 89.8%, and 91.2%, respectively, when compared to Erlotinib (IC_50_ = 0.03 μM, 95.4%). Furthermore, these compounds exhibited potent CDK-2 inhibition, with IC_50_ values of 0.301, 0.345, and 0.557 μM and inhibition percentages of 91.9%, 89.4%, and 88.7%, respectively, in contrast to Roscovitine (IC_50_ = 0.556 μM, 92.1%). RT-PCR analysis was performed on both untreated and **5g**-treated MCF-7 cells to confirm apoptotic cell death. Treatment with **5g** increased the gene expression of pro-apoptotic genes P53, Bax, caspases 3, 8, and 9 with notable fold changes while decreasing the expression of the anti-apoptotic gene Bcl-2. Molecular docking and dynamic simulations (100 ns simulation using AMBER22) were conducted to investigate the binding mode of the most potent candidates, namely, **5g**, **5l**, and **5n**, within the active sites of EGFR and CDK-2.

## Introduction

Cancer, a widespread and intricate group of diseases, presents a significant challenge in global healthcare, impacting millions of lives (Sung et al., 2021). Characterized by uncontrolled cell growth, it forms malignant tumors and persists as a major public health concern despite medical advancements. The multifaceted nature of cancer involves diverse forms and complex interactions of genetic, environmental, and lifestyle factors. The problem’s gravity is highlighted by its global prevalence and substantial emotional, economic, and healthcare burdens on individuals and communities. Urgency is fueled by rising incidence rates and the ongoing search for effective treatments and prevention. The introduction emphasizes the need to understand cancer’s intricacies for developing innovative therapies and prevention strategies, setting the stage for exploring its multifaceted aspects and addressing the challenges it poses to public health improvement.

Among women globally, breast cancer stood out as the predominant form of cancer, comprising 30% of the total newly diagnosed cases in the year 2021 (Sung et al., 2021). Breast cancer cell lines, such as MCF-7 and MDA-MB-231, play a crucial role in cancer research. Derived from breast cancer tumors, these cell lines serve as invaluable tools for studying the disease’s biology, testing treatments and understanding molecular mechanisms. They are cultured in laboratories, allowing researchers to investigate various aspects of breast cancer, including genetic makeup, treatment responses, and drug resistance. These cell lines are vital for the preclinical testing of new therapies, contributing to developing more effective treatments for breast cancer patients.

The distinct structural framework of these compounds, characterized by a spiro ring fusion at position-3 of the oxindole, gives rise to their diverse biological activities. This arrangement enables the oxindole moiety to serve as either a hydrogen bond donor or acceptor, thereby augmenting its interactions with diverse biological targets. Furthermore, their adaptability in forming combinations with various bioactive cycloalkyl or heterocyclic motifs substantially boosts their effectiveness across various applications (Zhou et al., 2020).

**FIGURE 1:**
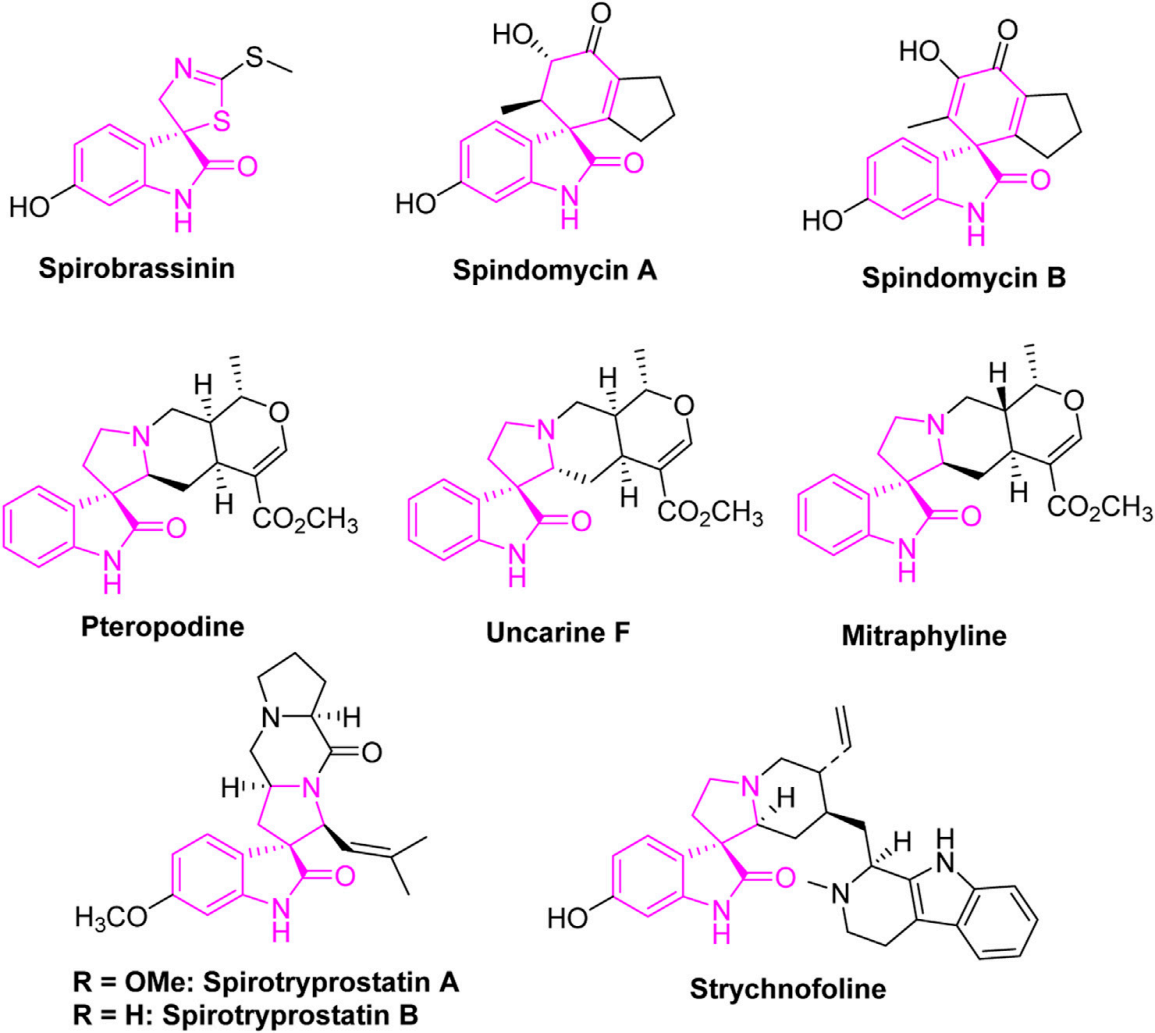
Naturally occurring spirooxindole compounds possessing anti-tumor properties.

Figure 1 depicts diverse spirooxindole frameworks sourced from nature, showcasing potent anti-cancer activity (Yu et al., 2015). Of particular note is Spirobrassinin, an oxindole alkaloid renowned for its robust anti-tumor properties (Budovská et al., 2020), along with spindomycins A and B, identified as potential inhibitors of the tyrosine kinase Bcr-Abl (Guo et al., 2014). Spirotryprostatins A and B exhibit noteworthy inhibitory effects against mouse breast cancer, specifically targeting tsFT210 (Ding et al., 2005; Al-Rashood et al., 2020). Pteropodine and Uncarine F have shown robust inhibitory effects against CEM-C7H2 cells, whereas Mitraphylline has exhibited significant inhibitory activity against various cancer cell lines, including neuroblastoma SKN-BE, glioma GAMG, human Ewing’s sarcoma MHH-ES-1, and breast cancer MT-3 cells in a dose-dependent manner (García Giménez et al., 2010). Strychnofoline is an additional example, demonstrating efficacy against melanoma and Ehrlich tumor cells (Yu et al., 2018; Yuenyongsawad et al., 2013). Mitraphylline, Uncarine F, and Pteropodine represent natural spirooxindole alkaloids extracted from Uncaria tomentosa (Bacher et al., 2006).

Spirotryprostatin A (Cui, et al., 1996; Edmondson et al., 1999) is an example of a neutrally occurring spirooxindole scaffold targeting breast cancer cells. Derived from the tryprostatin alkaloid family (Islam et al., 2023; Marti and Carreira, 2003), this compound exhibits notable inhibitory effects against breast cancer cell lines. The unique structural features of the spirooxindole scaffold, including a spiro ring fusion at position-3 of the oxindole, contribute to its ability to interact with biological targets in breast cancer cells. Studies suggest that Spirotryprostatin A hinders breast cancer cell proliferation and induces apoptosis, making it a potential candidate for further exploration in the development of targeted breast cancer therapies. The compound exemplifies the potential of spirooxindole scaffolds in the quest for innovative and effective treatments for breast cancer.

**FIGURE 2:**
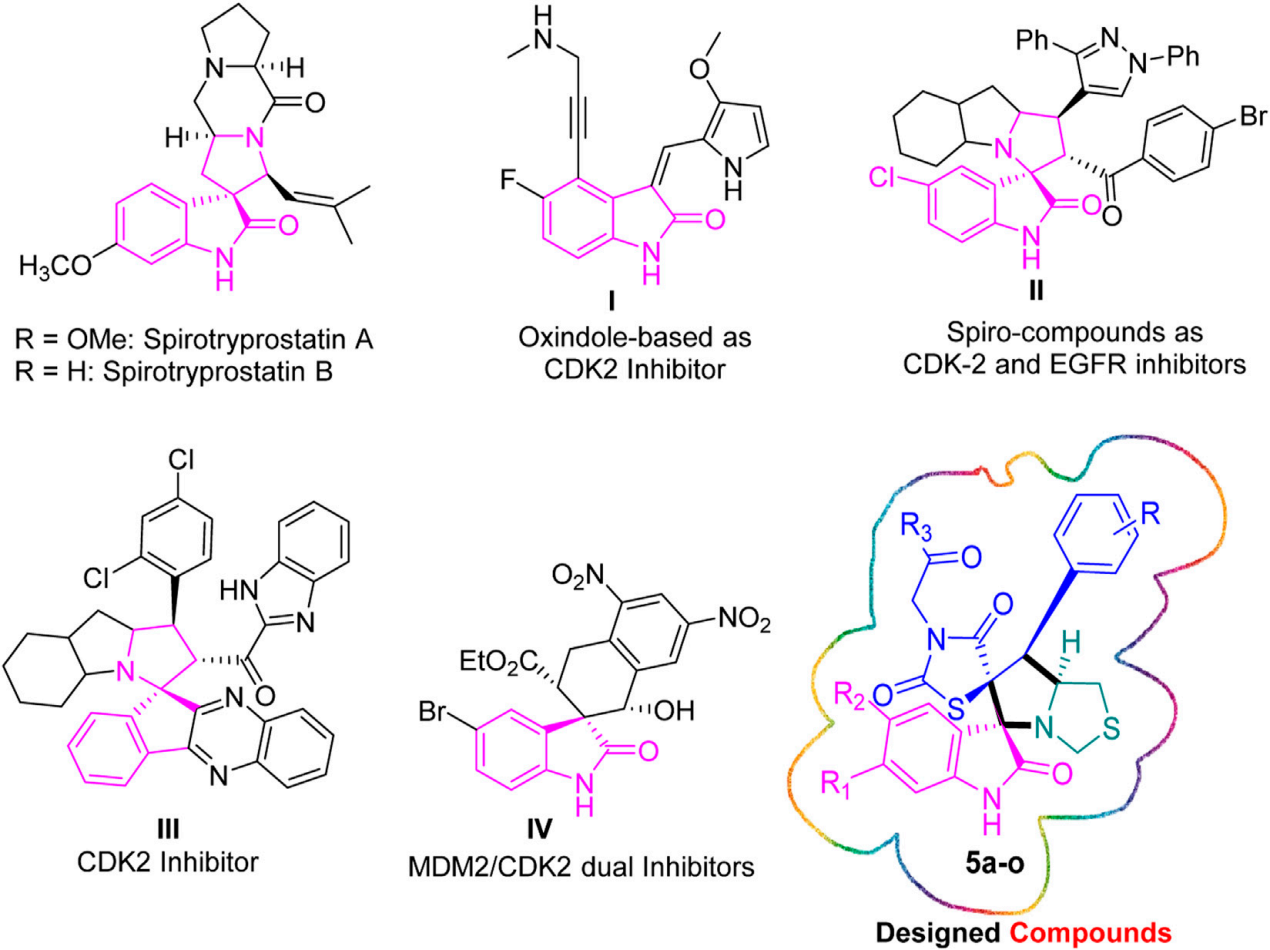
Rational design inspired by both natural products and novel synthetic spirooxindoles.

Cyclin-Dependent Kinase 2 (CDK2) (Tadesse, et al., 2018; Golsteyn, 2005) is a compelling target in cancer therapy due to its crucial role in regulating the cell cycle, particularly the transition from G1 to S phase. Aberrant activation of CDK2 is associated with uncontrolled cell proliferation in various cancers. Inhibitors designed to selectively target CDK2 have shown promise in preclinical and clinical settings by inducing cell cycle arrest and triggering apoptosis in cancer cells. Targeting CDK2 offers a strategic approach to impede cancer cell division, and ongoing research aims to optimize CDK2 inhibitors for enhanced efficacy and reduced side effects, highlighting its potential as an innovative avenue in cancer therapy. We built upon the groundwork established by benchmark oxindole-based CDK2 inhibitors (**I**) (Luk, 2004; Venkanna, et al., 2020; Bramson, et al., 2001) and spiro (Al-Jassas, et al., 2023; Barakat, et al., 2023) anti-cancer agents recognized for their kinase inhibition, specifically those aimed at CDK2. This rational study involved a detailed exploration of the CDK2 inhibitory potential within the investigated series, as depicted in Figure 2.

Al-Jassas (Al-Jassas, et al., 2023) designed, synthesized, and assessed a novel spirooxindole scaffold for its dual inhibitory properties against CDK2 and EGFR. Compound **II** exhibited notable inhibition, with IC_50_ values of 0.189 ± 0.01 µM (MCF-7) and 1.04 ± 0.21 µM (HepG2). Additionally, it demonstrated potent CDK-2 inhibition (34.98 nM) and an IC_50_ of 96.6 nM for EGFR inhibition. Compound **II** also effectively modulated the expression of pro-apoptotic genes (P53, Bax, caspases-3, 8, and 9) while downregulating the anti-apoptotic gene Bcl-2.

Barakat research group has reported a combinatorial stereoselective synthesis of rationally designed spiroindeno [1,2-*b*]quinoxaline-based CDK2 inhibitors **III** for non-small cell lung cancer (NSCLC) therapy (Barakat, et al., 2023). Among the derivatives tested, hit **III** emerged as the most promising, exhibiting potent inhibitory effects against A549 cells and normal lung fibroblasts Wi-38, with an IC_50_ value of 54 nM and a selectivity index (SI) of 6.64.

Biao Wang et al. (Wang, et al., 2020) identified the THN-fused spirooxindole derivative, **IV**, as a potent inhibitor using a rational drug design approach, complemented by the asymmetric synthesis of the designed compounds. Notably, **IV** exhibited robust inhibitory effects on both MDM2 and CDK4 in glioblastoma cells expressing either wild-type or mutant P53. Molecular dynamics simulations suggested a tight binding affinity of **IV** to both MDM2 and CDK4. Furthermore, **IV** demonstrated the ability to induce substantial apoptosis and G1 phase cell cycle arrest.

Based on the aforementioned findings, this study explores the realm of pyrrolidinyl-spirooxindole natural products, drawing inspiration from their distinctive chemical structures for the potential development of therapeutic agents. These naturally occurring compounds serve as intriguing templates, providing valuable insights into the design of novel medications. The investigation aims to unveil the therapeutic potential inherent in pyrrolidinyl-spirooxindoles, with the goal of developing innovative and effective therapeutic agents for diverse medical applications (Galliford and Scheidt., 2007). The study involves the synthesis and evaluation of a new set of spirooxindoles against breast cancer cells, along with an assessment of their inhibitory activities against CDK2 and EGFR. Additionally, the study explores apoptotic cell death, pro-apoptotic genes, and anti-apoptotic gene assays. Finally, molecular docking and dynamic simulations are employed to investigate the binding modes of the most potent candidates within the active sites of EGFR and CDK-2.

## Results and discussion

**SCHEME 1:**
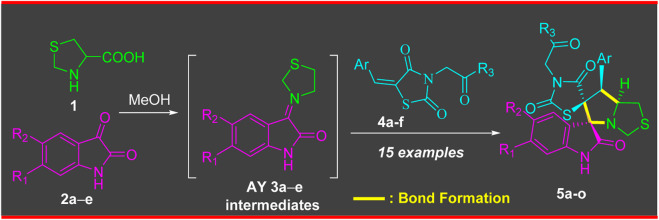
Synthesis of compounds **5a-o** *via* a 32CA reaction of AY **3a-e** with ethylene derivative **4a-f**.

Scheme 1 illustrates the efficient and highly selective synthesis of the targeted bi-spirooxindole-incorporated rhodanine analog. The starting material chalcones based rhodanine motif **4a-f**, was synthesized following a literature-reported method (Barakat et al., 2021). Employing a one-pot multicomponent 32CA reaction, the arylidene rhodanine analogue **4a-f**, isatin derivatives **2a-e**, and thioproline **1** were reacted under refluxed conditions in MeOH for 2 h, resulting in the desired stereo-selective bi-spirooxindole-incorporated rhodanine analog **5a-o**. The reaction proceeded in two steps: first, isatin derivatives **2a-e** reacted with secondary amino acid (thioproline) **1** to generate the azomethine ylide (AY). In the second step, the generated azomethine ylide (AY 3) reacted with arylidene rhodanine analog **4a-f** through completely *ortho* regioselective and *exo* stereoiselective. Spectral data analysis and elucidation confirmed the proposed structure, and single crystal X-ray diffraction analysis further validated the chemical structure.

### Crystal structure description

**FIGURE 3:**
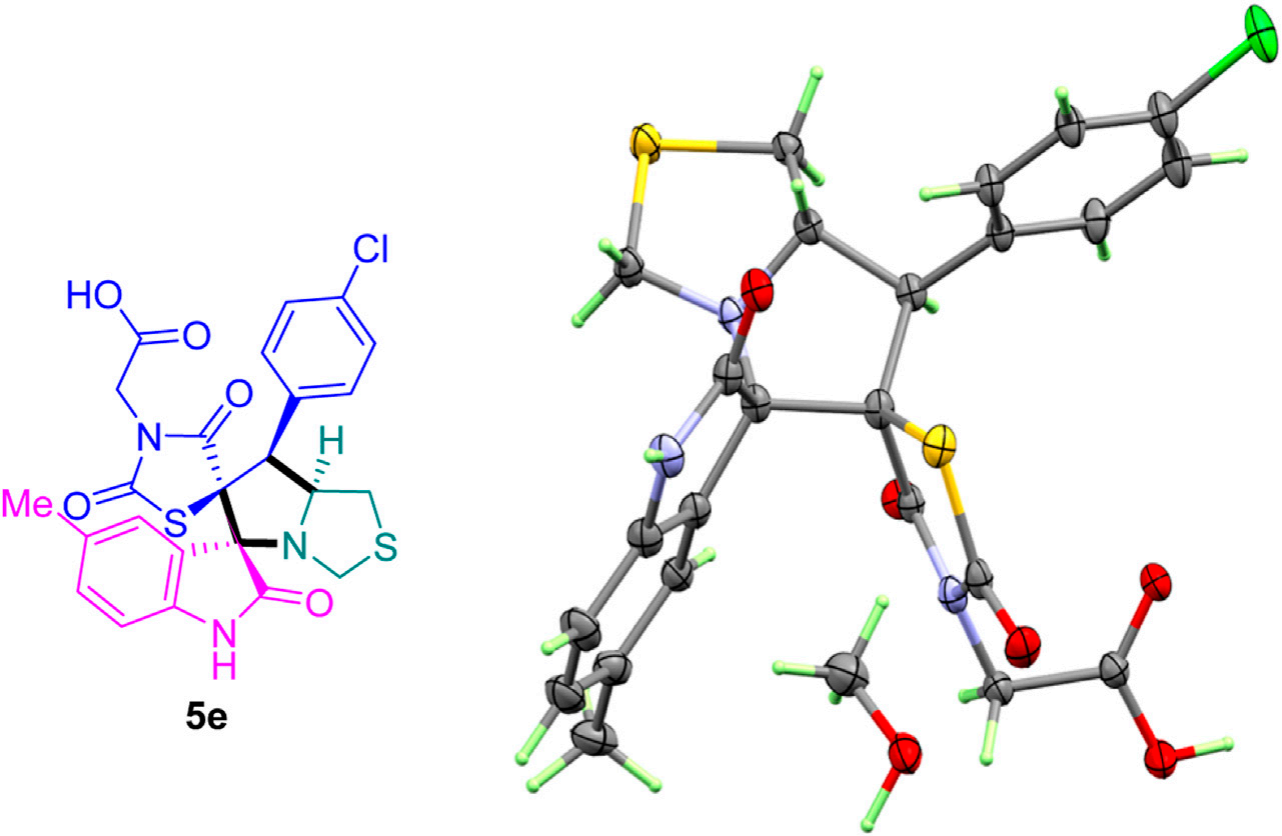
ORTEP for compound **5e.**

The X-ray structure of the studied compound **5e** (Figure 3) revealed the formation of the target organic hybrid, which crystallized with one molecule of methanol as a crystal solvent. It crystallized in monoclinic crystal system and *P2*_*1*_ */n* as a space group. The unit cell parameters are *a* = 11.5475 (3), *b* = 15.7550 (4) *c* = 14.6493 (3) Å and *β* = 104.655 (2)˚. There is one molecule as asymmetric formula while *z* = 4. It is evident from the reported X-ray structure the presence of four stereogenic centers located at C9, C12, C13 and C14 atoms. This indicated and assigned the absolute configuration of the final spiroxindoles adduct.

### Cytotoxic activity

The cytotoxicity of the synthesized compounds was tested using the MTT assay on breast cancer cells (MCF-7 and MDA-MB-231). As seen in Table 1, compounds **5l-5o** showed potent cytotoxicity against MCF-7 cells with IC_50_ values range of 3.4–4.5 μM compared to Erlotinib (IC_50_ = 2.14 µM), and they exhibited potent cytotoxicity against MDA-MB-231 with IC_50_ values range of 4.3–8.4 μM compared Erlotinib (IC_50_ = 3.25 µM). Compounds **5a-f** showed promising cytotoxicity against MCF-7 cells with IC_50_ values range of 5.87–18.5 μM, with selective cytotoxicity against MDA-MB-231 cancer cells with higher IC_50_ values. Interestingly, compound **5g** had the highest cytotoxicity among the tested compounds, with IC_50_ value of 2.8 μM. Furthermore, potent compounds **5g**, **5l,** and **5n** were safe (non-cytotoxic) against the WISH cells with higher IC_50_ values with an IC_50_ value range of 39.33–47.2 μM.

**TABLE 1:** Cytotoxicity of the tested compounds against MCF-7 and MDA-MB-231 breast cancer cells using the MTT assay.

Chemical structures	IC_50_ ± SD [μM]		
	MCF-7	MDA-MB-231	WISH
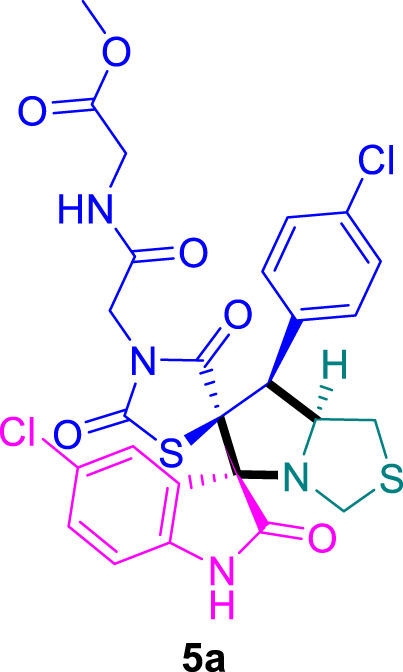	5.87 ± 0.5	29.5 ± 1.3	NT
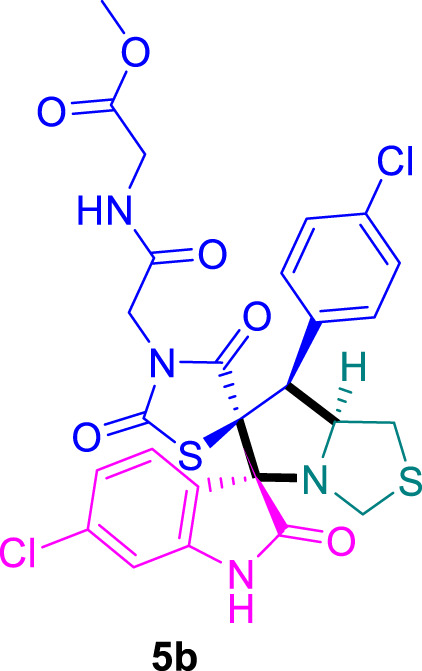	7.8 ± 0.32	32.5 ± 0.42	NT
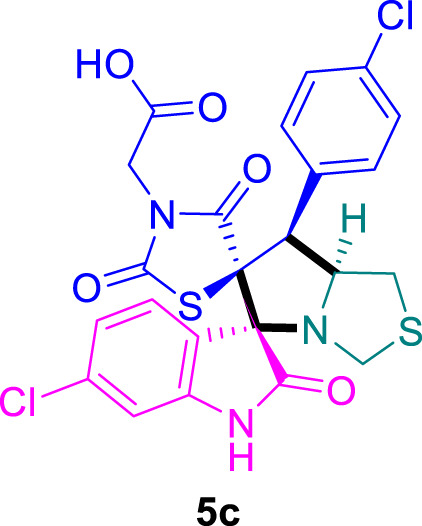	15.8 ± 0.4	24.2 ± 1.1	NT
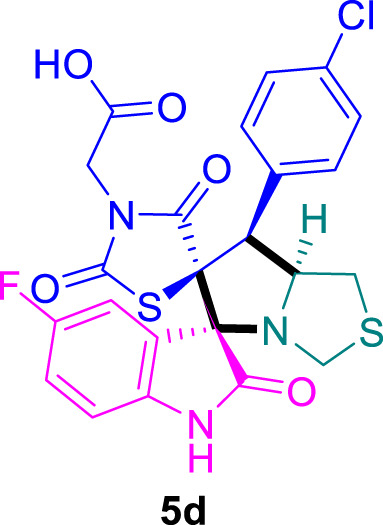	19.4 ± 0.4	9.8 ± 0.35	NT
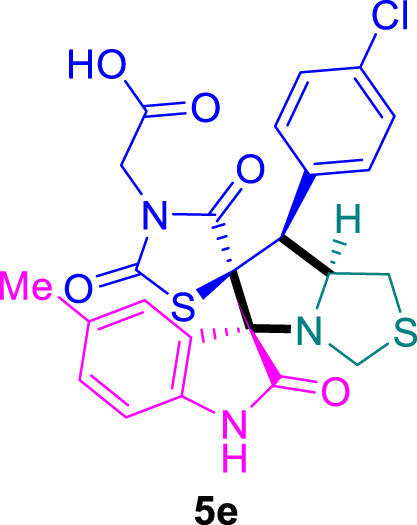	9.8 ± 0.7	5.4 ± 0.6	NT
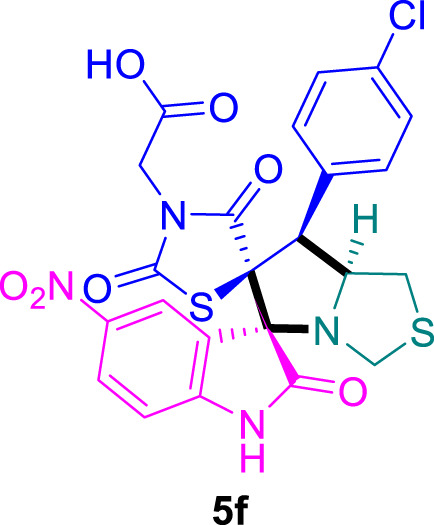	18.5 ± 0.6	35.5 ± 1.1	NT
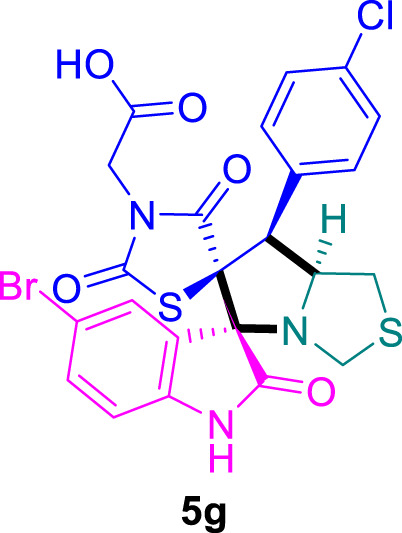	2.8 ± 0.4	23.5 ± 0.9	39.33 ± 1.8
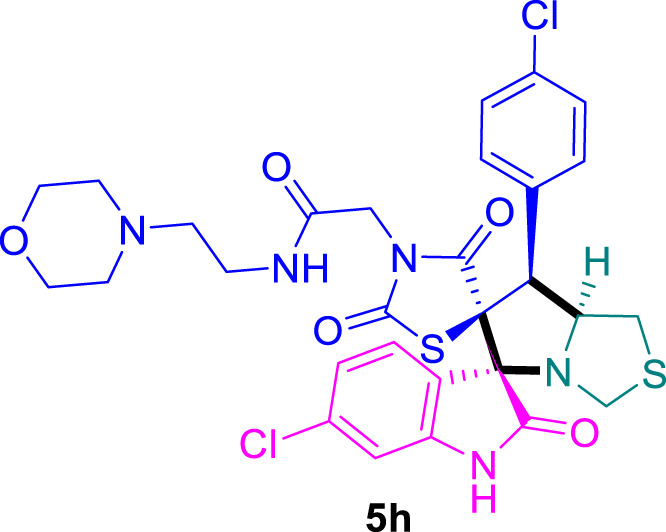	24.3 ± 0.9	31.5 ± 1.0	NT
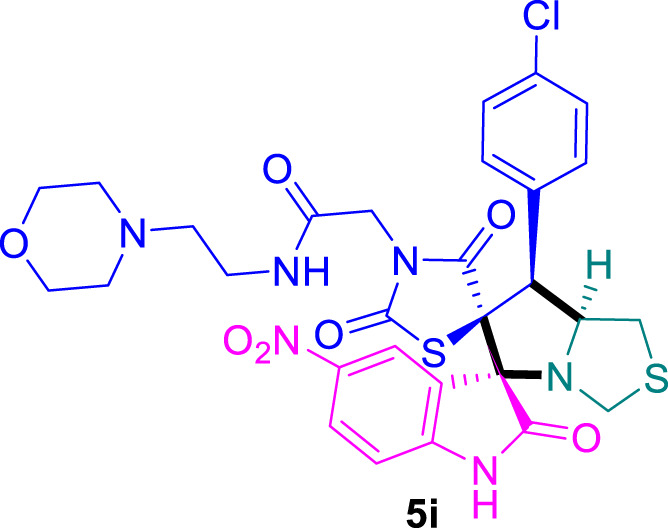	28.7 ± 0.8	35.3 ± 1.2	NT
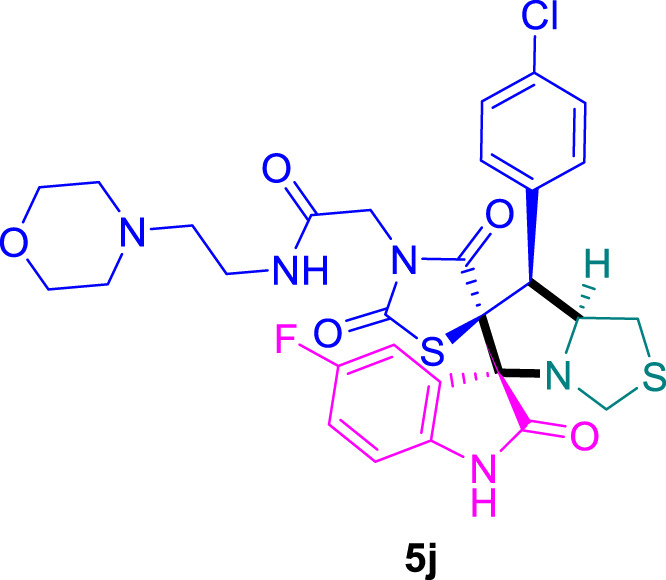	23.4 ± 1.2	21.4 ± 0.8	NT
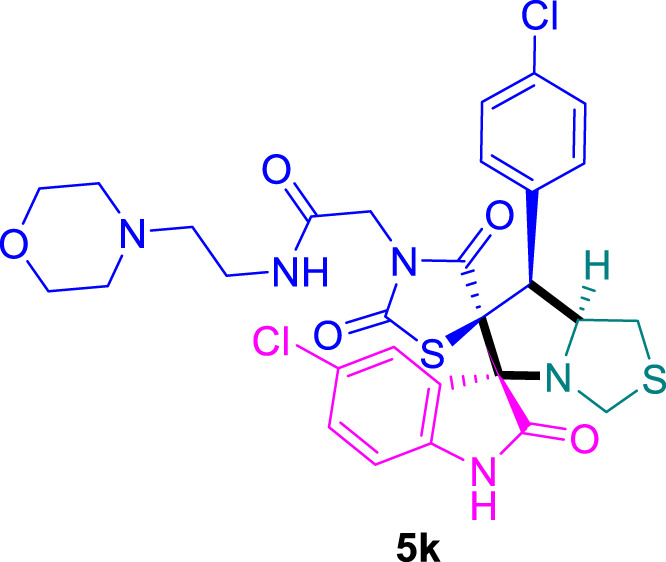	21.4 ± 0.9	19.5 ± 0.7	NT
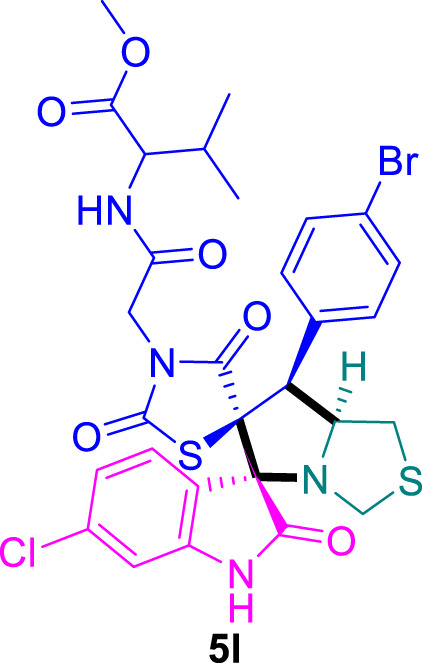	3.4 ± 0.5	8.43 ± 0.6	43.5 ± 2.0
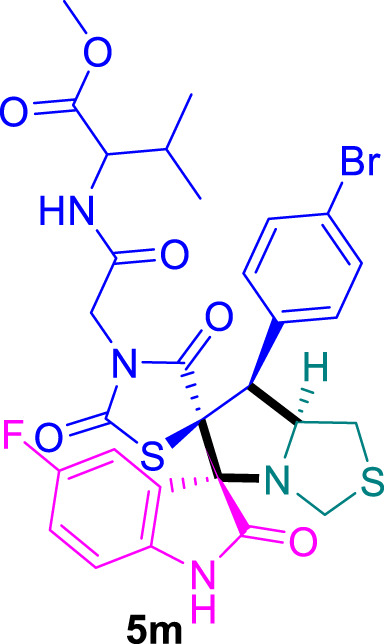	4.5 ± 0.4	6.3 ± 0.3	NT
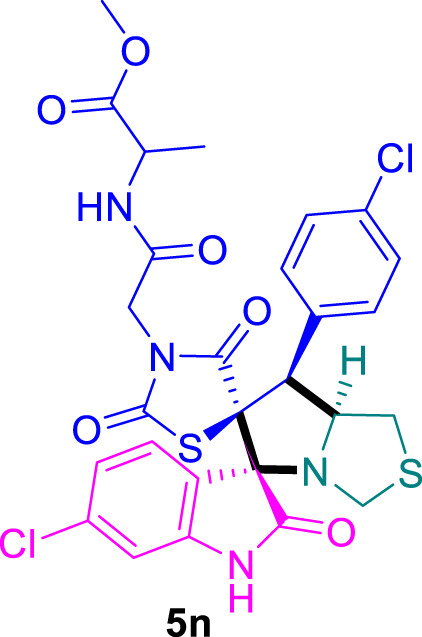	3.9 ± 0.3	5.3 ± 0.4	47.2 ± 2.1
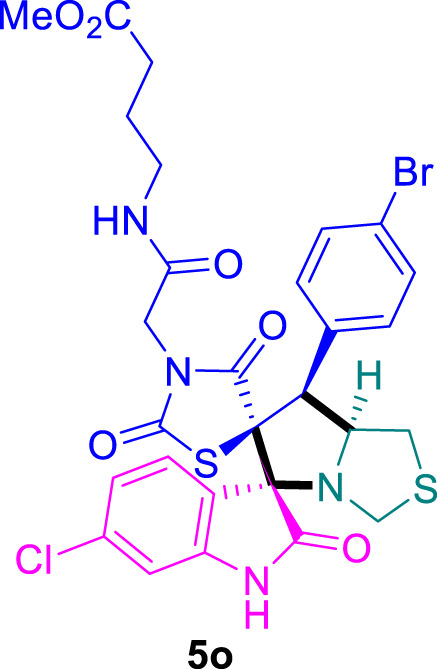	4.12 ± 0.4	4.32 ± 0.5	NT
Standard: Erlotinib	2.14 ± 0.3	3.25 ± 0.5	NT

*Values are expressed as Mean ± SD, of three independent trials. NT: non tested.

### EGFR/CDK-2 inhibition

Inhibitory activities of **5g, 5l,** and **5n** were tested against EGFR and CDK-2. Interestingly, as seen in Table 2, they exhibited potent EGFR inhibition, with IC_50_ values of 0.026, 0.067, and 0.04 μM with percentages of inhibition of 92.6%, 89.8%, 91.2% compared to Erlotinib (IC_50_ = 0.03 μM, 95.4%). Additionally, they exhibited potent CDK-2 inhibition, with IC_50_ values of 0.301, 0.345, and 0.557 μM with percentages of inhibition of 91.9%, 89.4%, 88.7% compared to Roscovitine (IC_50_ = 0.556 μM, 92.1%). These findings highlight the promising EGFR/CDK-2 enzyme inhibition.

**TABLE 2:** IC_50_ values of EGFR and CDK-2 kinase activities of the tested compounds.

Compound	EGFR kinase		CDK-2 kinase	
	IC_50_ [μM]^a^	% Of EGFR inhibition	IC_50_ [μM]^a^	% Of CDK-2 inhibition
5 g	0.026 ± 0.006	92.6 ± 1.9	0.301 ± 0.011	91.9 ± 2.1
5l	0.067 ± 0.001	89.8 ± 2.8	0.345 ± 0.011	89.4 ± 2.8
5n	0.04 ± 0.001	91.2 ± 2.1	0.557 ± 0.017	88.7 ± 1,9
Erlotinib	0.03 ± 0.002	95.4 ± 2.7	--	--
Roscovitine	--	--	0.556 ± 0.001	92.1 ± 2.7

^a^ Values are expressed as an average of three independent replicates. IC_50_ values were calculated using sigmoidal non-linear regression curve fit of percentage inhibition against five concentrations of each compound.

### Apoptotic investigation

#### Annexin V/PI staining with cell cycle analysis

**FIGURE 4:**
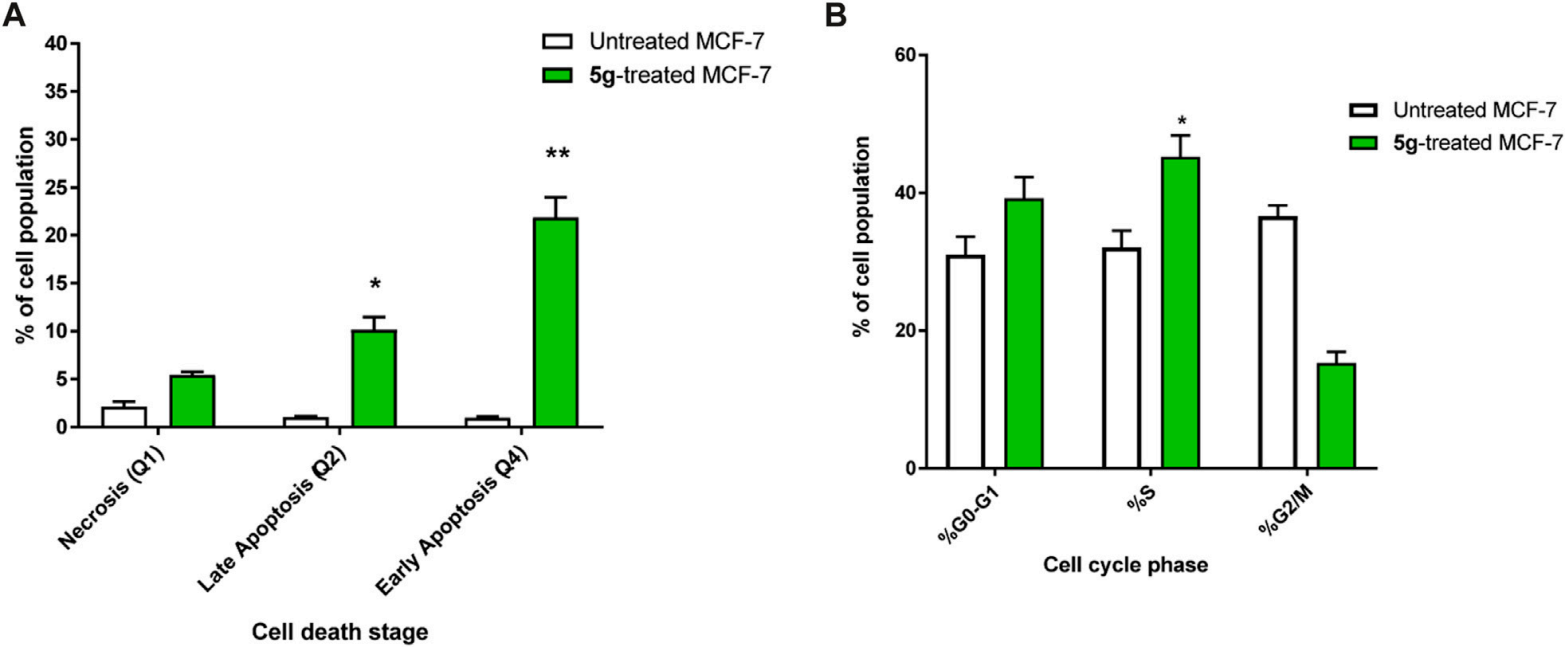
**(A)** Bar representation of the percentage of apoptosis (early and late apoptotic cell population) using Annexin V/Propidium Iodide staining. **(B)** Bar representation of the percentage of percentage of cell population at each cell phase in untreated and **5g**-treated MCF-7 cells, at the IC_50_ value at 48 h “Data shown are the average of three independent experimental runs (Mean ± SD). *p ≤ 0.05 and **p < 0.001 compared to untreated control”.

The apoptotic activity of compounds **5g** was determined by flow cytometric analysis of Annexin V/PI staining of untreated and treated MCF-7 cells. Figure 4A) showed that compounds **5g** significantly activated apoptotic cell death, increasing the cell population in total apoptosis by 31.9% (10.15% late and 21.87% early apoptosis) compared to the untreated control group (1.98%). Additionally, they induced necrotic cell death by 5.43% compared to 2.12% in the untreated control. Hence, compound **5g**-treatment induced apoptosis more than necrotic cell death.

Additionally, As can be shown in Figure 4B), the cell population in the G0-G1-phase was considerably raised by 39.8% after treatment with compound **5g**, compared to the control 31%, whereas the cell population in the S-phase was significantly increased by 45.2% after treatment compared to the control 32.1%, hence, in contrast, cells population at G2/M phase were decreased upon treatment.

#### RT-PCR gene expression of apoptosis-related genes

**FIGURE 5:**
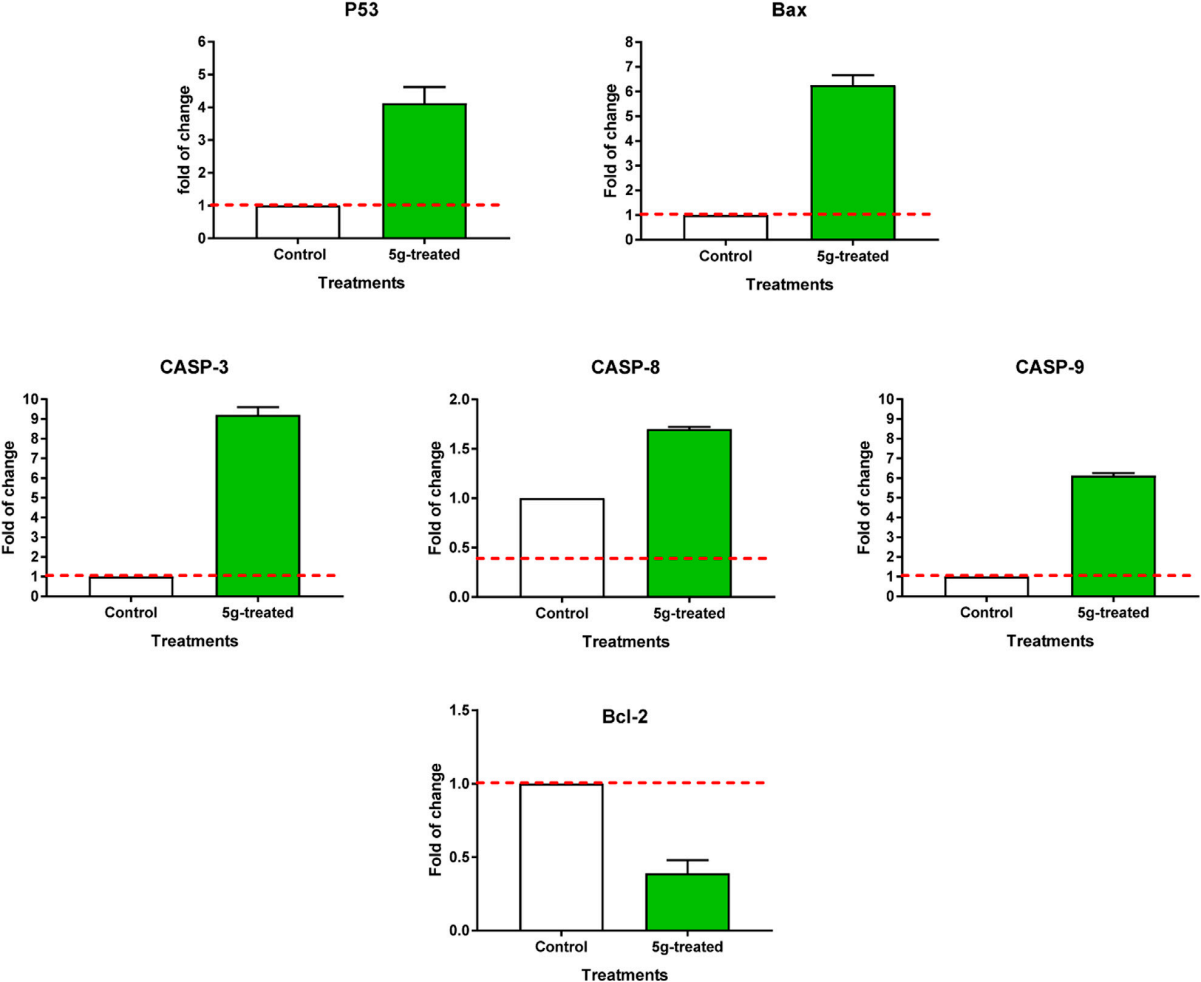
RT-PCR of apoptosis-related genes of untreated and **5g**-treated MCF-7 cells at the IC_50_ value. “Values are expressed as Mean ± SD of three independent replicates”. “Data were normalized using β-actin as house-keeping gene. The dashed line represents the gene expression level of untreated control. Fold change was calculated using 2^^- ΔΔCT^”.

Both the untreated and treated MCF-7 cells were subjected to RT-PCR to confirm apoptotic cell death (Figure 5). The expression of pro-apoptotic genes P53, Bax, caspases 3, 8, and 9 was upregulated by **5g** treatment, with corresponding fold changes of 4.1, 6.26, 9.2, 1.7, and 6.13, respectively. Concurrently, it resulted in a 0.39-fold reduction in the expression of the anti-apoptotic gene Bcl-2. These findings are in line with the possibility of triggering cell death by blocking enzymes. Activation of the intrinsic apoptotic pathway leads to mitochondrial potential loss and cytochrome c release. When the ratio of proteins that promote cell death to those that prevent it rises, a cascade reaction involving caspases 3 and 9 is set in motion, leading to cell death by caspase-dependent apoptosis.

**FIGURE 6:**
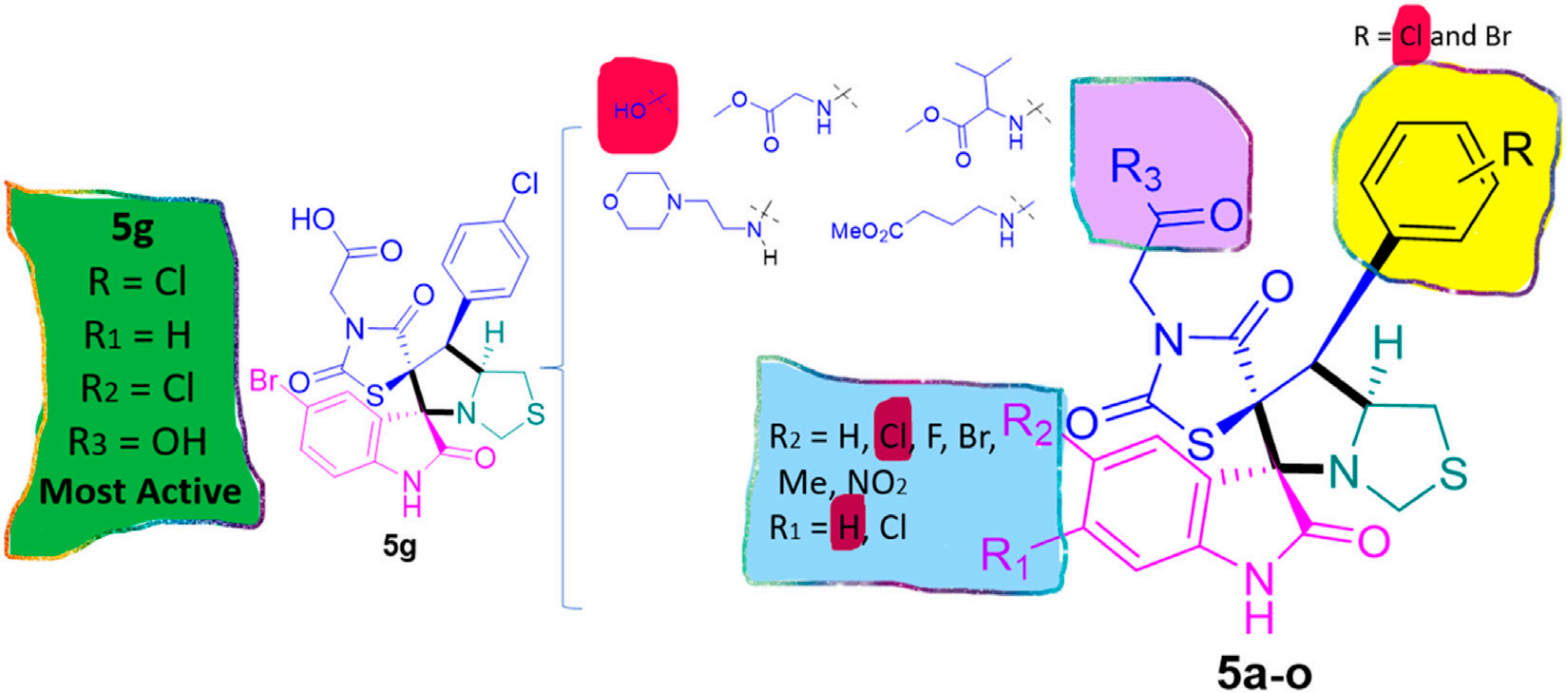
Structure reactivity relationship (SAR) of the synthesized compounds.

Structure-Activity Relationship (SAR) analysis is pivotal in understanding the correlation between the structural features of the synthesized spirooxindoles and their biological activities. In the case of these compounds inspired by pyrrolidinyl-spirooxindole natural products, SAR exploration involves assessing how variations in the molecular structure impact cytotoxicity against specific cancer cell lines, inhibitory activities against enzymes like EGFR and CDK-2, and safety profiles. The goal is to identify key structural elements that contribute to therapeutic efficacy, guiding further optimization for the development of more effective compounds. The results indicate that among the synthesized library of inspired spiroxindoles, compound **5g** stands out as the most active. Notably, it features a *p*-Cl-substituted benzene, 5-Cl-substituted oxindole, and *N*-substituted acid in its chemical structure (Figure 6).

#### Molecular docking study

**FIGURE 7:**
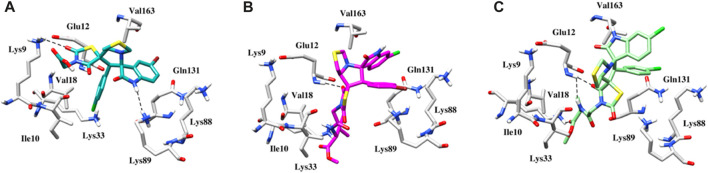
**(A)** Binding pose of the **5g** within the active site of CDK2 protein. **(B)** Binding pose of the **5l** within the active site of CDK2 protein. **(C)** Binding pose of the **5n** within the active site of CDK2 protein. Interactions between hydrogen bonds are shown by the dashed black lines.

Using the MOE docking suite, the most active compound of the series and the reference anti-cancer drugs roscovitine and erlotinib were docked into the active sites of the target proteins CDK2 (PDB ID 6Q4G) (Wood et al., 2019) and EGFR (PDB ID 1M17) (Stamos et al., 2002), respectively, to explore the anti-cancer potential of spirooxindole engrafted rhodanine derivatives. The binding modes of the most active compounds, **5g**, **5l**, and **5n**, were established using MOE with binding energies ranging from −5.3 to −7.6 kcal mol-1. Figure 7A depicts the binding pose of compound **5g**, where the oxygen of the dioxothiazolidin ring is involved in a hydrogen bond interaction with the nitrogen of the side chain of Lys9 at a distance of 3.2Å. The protein-ligand interactions are further stabilized by hydrophobic interactions between the ligand and Ile10, Lys88, and Val163 of the CDK2 protein. In the case of compound **5l**, a strong hydrogen bond interaction is observed between the oxygen of the dioxothiazolidin ring and the side chain of Glu12 at a distance of 2.1Å (Figure 7B). Meanwhile, in the case of compound **5n**, which is the most active compound of the series, two hydrogen bond interactions are exhibited with the main chain of Glu12 at distances of 1.9 and 3.4Å (Figure 7C). Another hydrogen bond is observed between the oxygen of the dioxothiazolidin ring and the side chain of Lys89 at a distance of 3.4Å. Apart from the H-bond interactions, an oxygen atom of compounds **5l** and **5n** participates in a salt bridge interaction with the positively charged Lys33 residue. These compounds also exhibit hydrophobic interactions with Val18, Gln131, and Val163.

**FIGURE 8:**
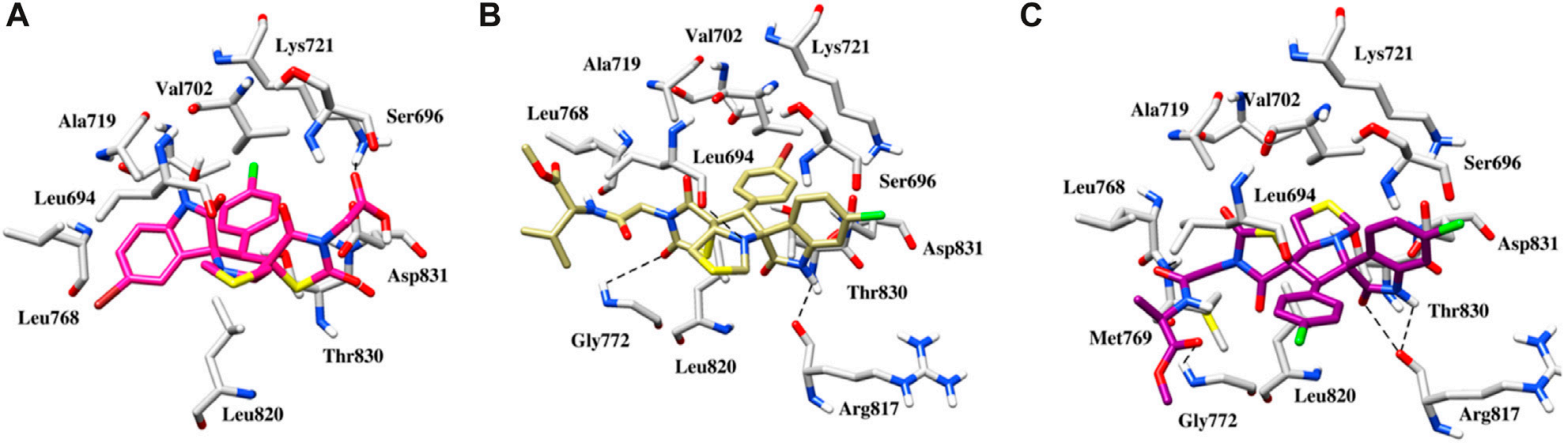
**(A)** Binding pose of the **5g** within the active site of EGFR protein. **(B)** Binding pose of the **5l** within the active site of EGFR protein. **(C)** Binding pose of the **5n** within the active site of EGFR protein. The dashed black line depicts hydrogen bond interaction.

In EGFR docking, the conformation exhibiting the most favorable binding energy (approximately −7.4 to −8.3 kcal mol-1) substantiates the notion that the compounds are effectively incorporated within the binding pocket. The binding patterns are also slightly different, which may be responsible for the variations in activity. It is important to mention that the reference inhibitor forms a hydrogen bond with the backbone NH of Met769 in the Hinge region, while the compound is deeply embedded into the EGFR active site *via* hydrophobic interactions that are conserved in the majority of the structures. In the case of compound **5g**, in addition to hydrophobic interactions, a salt bridge interaction is also observed with Lys721 (Figure 8A). The plausible binding mode of compound **5l** is depicted in Figure 8B. The nitrogen of the thiazole and indolin ring is involved in a hydrogen bond interaction with the oxygen of Leu694 and Arg817 at distances of 3.3 and 3.5 Å, respectively. Another hydrogen bond interaction is observed between the oxygen of the dioxothiazolidin ring and the side chain of Gly772 at a distance of 3.3 Å. Figure 8C presents the binding mode of compound **5n**. This compound also exhibits three hydrogen bond interactions with Leu694, Gly772, and Arg817 at distances of 3.4, 3.3, and 2.5 Å, respectively. Further, anchorage is provided by hydrophobic interactions with Leu694, Val702, Leu768, and Leu820.

A 100 ns simulation using AMBER22 was conducted to understand the dynamic behavior of the active chemical. An evaluation of the system’s overall stability and simulation quality was conducted using the RMSD, RMSF, and RG (radius of gyration) as metrics for quantitative analysis (RoG).

**FIGURE 9:**
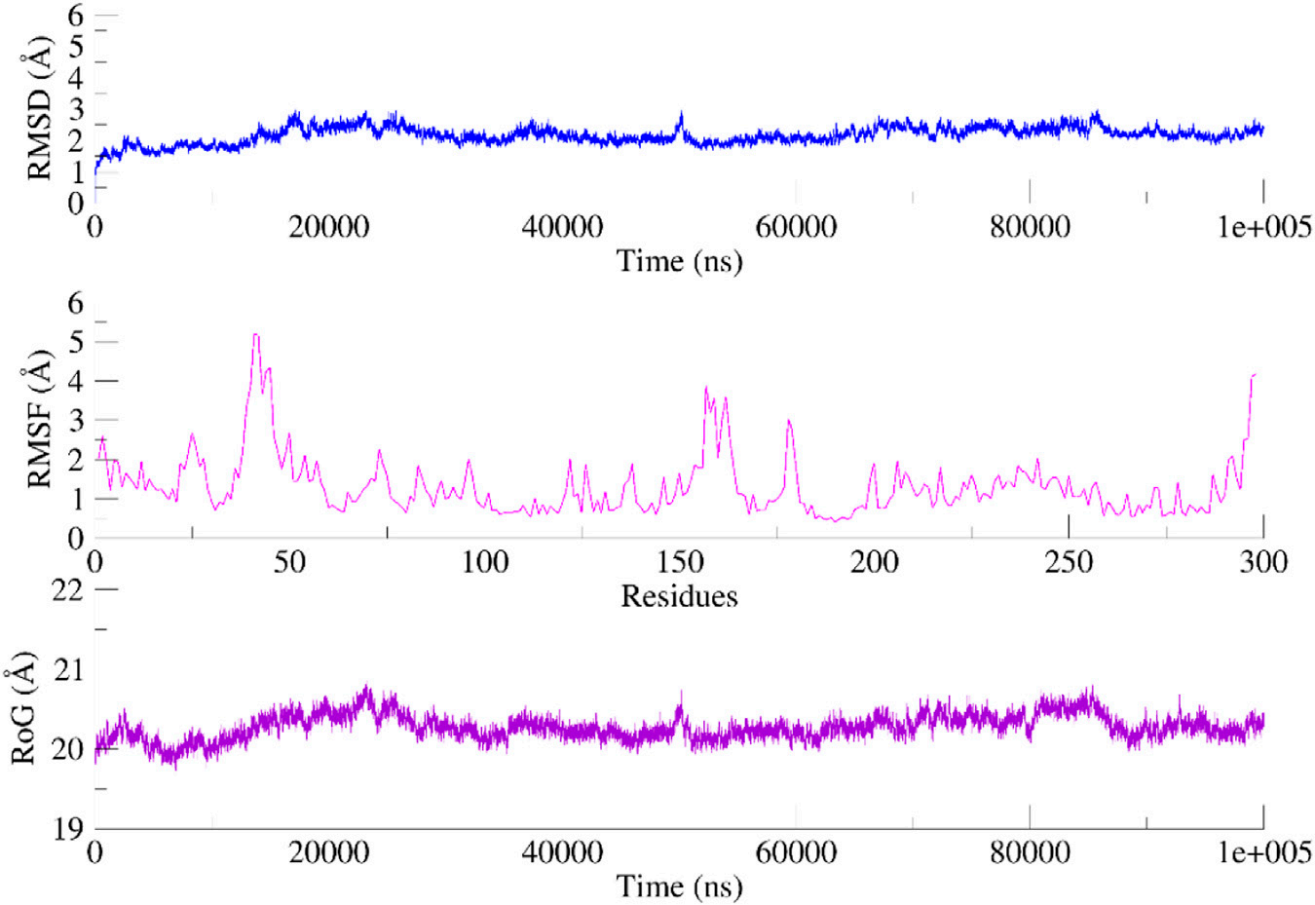
RMSD, RMSF and RoG of the CDK2 systems calculated as a function of time.

**FIGURE 10:**
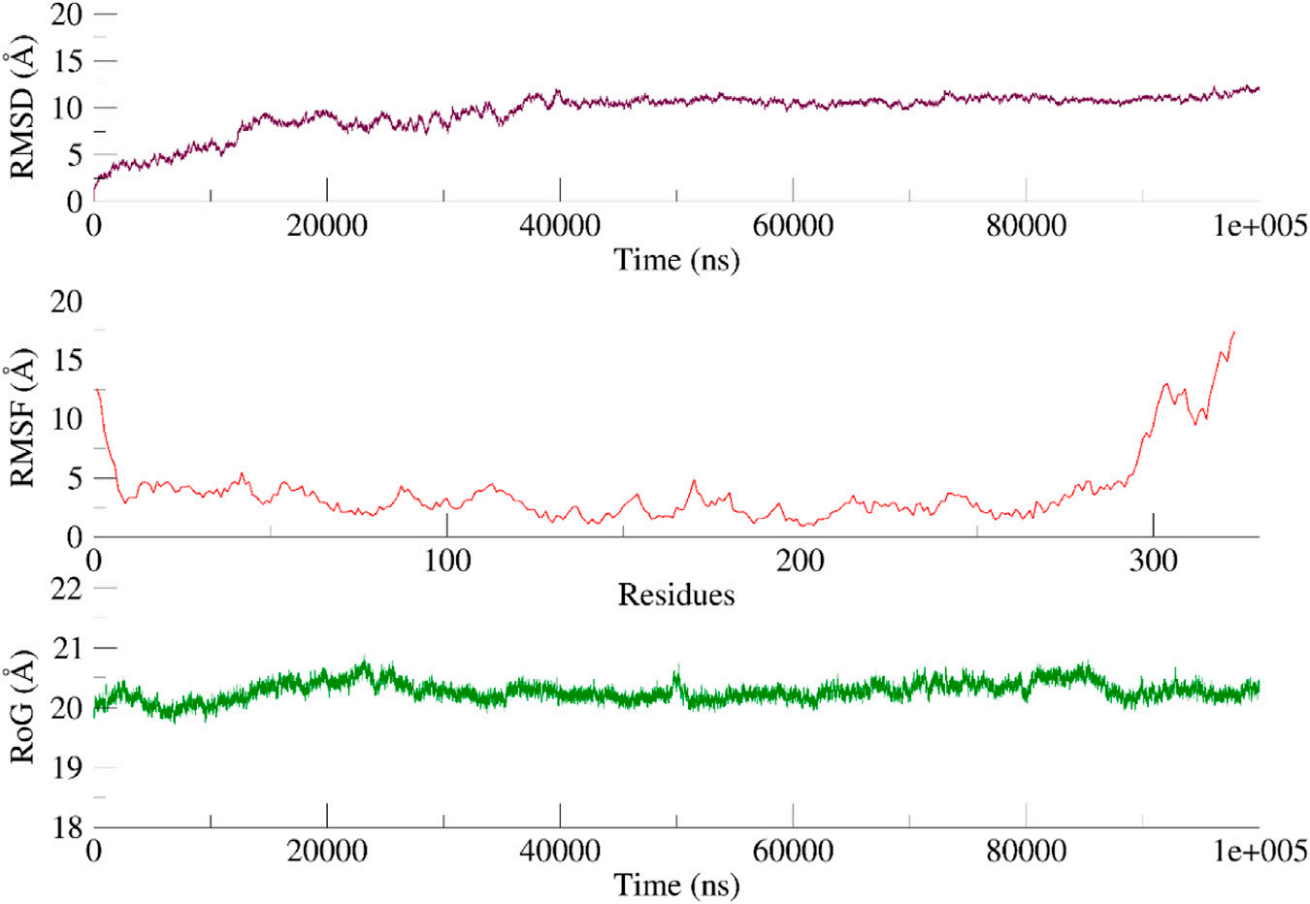
RMSD, RMSF and RoG of the EGFR systems calculated as a function of time Based on the visual examination of simulation trajectories, it can be inferred that compound **5n** achieves stability within the cavity of CDK2 and EGFR by effectively facilitating hydrophobic and hydrophilic contacts with the active site residue. The examination of time-varying paths of **5n** reveals intriguing findings. The polar atoms of the dioxothiazolidin moiety maintain contact with the protein through polar interactions with Glu12 and Lys89, with occupancies of 65% and 55%, respectively.

The RMSD of the heavy atoms in the main chain of the proteins was computed using the “rms” tool in CPPTRAJ. Figures 9, 10; Supplementary Figure S10 illustrate the RMSD of the heavy atoms in the protein backbone. Figures 7, 8 clearly demonstrate the system’s stability, as indicated by an average RMSD value of 2.7 Å in the case of CDK2. In the case of EGFR, higher RMSD is observed due to flexibility in its domain. This observation was further corroborated by examining the RoG, indicating that the systems were tightly compressed (Figures 7, 8). In addition, to comprehend the behavior of the side chains of residues, the RMSF of the protein was computed over time (Figures 9, 10). The results indicated that the amino acid residues in the protein-ligand complex remained stable upon interaction with the active chemical of the series.

Regarding EGFR, a visual examination of the paths shows that **5n** engages in hydrophobic interactions with Leu694, Val702, Leu768, and Leu820. Compound **5n** demonstrates a significant affinity for the “hinge region key residue,” Met769, of the EGFR target. This residue is essential for the active site and is occupied 68% of the time. Throughout the simulation, the majority of the protein-ligand interactions were observed to align with the docking position.

## Materials and methods

### General notes

“Isatin derivatives **2a-e** and thioproline **1** are commercially available. ^1^H-NMR and ^13^C-NMR are recorded in DMSO-*d*_6_ (JEOL Spectrometer (400 MHz). The X-ray diffraction data was collected on a Rigaku Oxford Diffraction Supernova diffractometer using Cu Kα radiation. The desired starting material **4a-f** was synthesized according to reported literature Abd Alhameed et al., 2020). The desired sprio-compounds derived rhodanine amino-acids **5h-k** was synthesized according to previous reported literature (Barakat et al., 2021)”.

### General procedure for the synthesis of spiro compounds analogues 5a-o

A mixture of three components reaction including substituted isatin **2a-e** (0.5 mmol), L-thioproline **1** (66.5 mg, 0.5 mmol), and compounds **4a-f** (0.5 mmol) were refluxed on oil bath for 2 h. After completion of the reaction, as evident from (TLC Eluent: Ethyl acetate: *n-*Hexane 40%), Without additional purification, the reaction mixture was left at room temperature overnight to slowly evaporate. The solid crystalline components were filtered out to give compounds **5a-o**, which were solid compounds with a light faint yellow color and an 80%–90% chemical yield.

### Methyl (2-((3*S*,6′*S*,7′*S*,7a′*S*)-5-chloro-7'-(4-chlorophenyl)-2,2″,4″-trioxo-7′,7a′-dihydro-1′*H*,3′*H*-dispiro [indoline-3,5′-pyrrolo [1,2-*c*]thiazole-6′,5″-thiazolidin]-3″-yl)acetyl)glycinate 5a

The 5-chloro-isatin **2a** (90.5 mg) and **4a** (192.0 mg) were utilized according to the general method, and the sprio-compound **5a** was obtained in 90% yield.

^1^H NMR (400 MHz, DMSO-*d*_6_) δ 11.17 (s, 1H), 8.65 (t, *J* = 5.8 Hz, 1H), 7.69 (d, *J* = 8.7 Hz, 1H), 7.63 (d, *J* = 8.7 Hz, 1H), 7.52–7.36 (m, 4H), 7.20 (d, *J* = 2.5 Hz, 1H), 6.90 (d, *J* = 8.2 Hz, 1H), 4.81 (q, *J* = 7.8 Hz, 1H), 4.35 (s, 1H), 4.19–4.06 (m, 2H), 4.00 (d, *J* = 16.2 Hz, 1H), 3.91 (dd, *J* = 6.1, 3.0 Hz, 3H), 3.78 (d, *J* = 6.0 Hz, 1H), 3.45 (d, *J* = 5.9 Hz, 1H), 2.99 (dd, *J* = 9.5, 5.7 Hz, 1H), 2.82–2.71 (m, 1H).^13^C-NMR (100 Hz, DMSO-*d*_6_): *δ* 176.1, 174.5, 168.6, 164.7, 158.2, 154.2, 151.1, 145.4, 135.9, 135.6, 133.4, 132.3, 131.5, 129.1, 121.6, 110.8, 76.2, 75.5, 70.1, 66.7, 57.7, 55.9, 53.8, 47.1, 43.9, 36.7, 33.3; Chemical Formula: C_26_H_22_Cl_2_N_4_O_6_S_2_; LCMS (*m*/*z*): 622.54 [M + H]^+^, Elemental Analysis: [Calculated: C, 50.25; H, 3.57; N, 9.01; S, 10.32; Found: C, 50.30; H, 3.60; N, 9.11; S, 10.40].

### Methyl (2-((3*S*,6′*S*,7′*S*,7a′*S*)-6-chloro-7'-(4-chlorophenyl)-2,2″,4″-trioxo-7′,7a′-dihydro-1′*H*,3′*H*-dispiro [indoline-3,5′-pyrrolo [1,2-*c*]thiazole-6′,5″-thiazolidin]-3″-yl)acetyl)glycinate 5b

The 6-chloro-isatin **2b** (90.5 mg) and **4a** (192.0 mg) were utilized according to the general method, and the spiro compound **5b** was obtained in 91% yield.

^1^H NMR (400 MHz, DMSO-*d*_6_) δ 11.18 (s, 1H), 8.61 (t, *J* = 5.8 Hz, 1H), 7.51–7.37 (m, 5H), 7.25 (d, *J* = 8.1 Hz, 1H), 7.05 (dd, *J* = 8.3, 2.2 Hz, 1H), 6.90 (d, *J* = 2.1 Hz, 1H), 4.88–4.76 (m, 1H), 4.35 (s, 1H), 4.17 (d, *J* = 9.4 Hz, 1H), 4.04 (d, *J* = 6.7 Hz, 2H), 3.89 (d, *J* = 6.0 Hz, 2H), 3.82 (d, *J* = 6.4 Hz, 1H), 3.45 (d, *J* = 6.0 Hz, 1H), 3.01 (dd, *J* = 9.6, 5.7 Hz, 1H);^13^C NMR (101 MHz, DMSO-*d*_6_) δ 207.15, 178.72, 176.21, 174.69, 170.53, 169.03, 168.67, 164.42, 146.26, 145.35, 138.13, 135.92, 133.48, 132.21, 130.31, 129.29, 121.47, 105.91, 104.02, 95.33, 94.30, 76.20, 75.51, 74.09, 60.51, 40.68, 40.47, 40.26, 40.15, 40.05, 39.84, 39.63, 39.43, 31.34; Chemical Formula: C_26_H_22_Cl_2_N_4_O_6_S_2_; LCMS (*m*/*z*): 622.54 [M + H]^+^, Elemental Analysis: [Calculated: C, 50.25; H, 3.57; N, 9.01; S, 10.32; Found: C, 50.31; H, 3.60; N, 9.12; S, 10.39].

### 2-((3*S*,6′*S*,7′*S*,7a′*S*)-6-chloro-7'-(4-chlorophenyl)-2,2″,4″-trioxo-7′,7a′-dihydro-1′H,3′H-dispiro [indoline-3,5′-pyrrolo [1,2-*c*]thiazole-6′,5″-thiazolidin]-3″-yl)acetic acid 5c

The 6-chloro-isatin **2b** (90.5 mg) and **4b** (156.0 mg) were utilized according to the general method, and the spiro-compound **5c** was obtained in 89% yield.

^1^H NMR (400 MHz, DMSO-*d*_6_) δ 11.19 (s, 1H), 7.50–7.36 (m, 5H), 7.29 (d, *J* = 8.5 Hz, 1H), 7.06 (d, *J* = 8.6 Hz, 1H), 6.90 (s, 1H), 4.88–4.77 (m, 1H), 4.22–4.11 (m, 2H), 4.05 (d, *J* = 17.4 Hz, 1H), 3.84 (d, *J* = 6.3 Hz, 1H), 3.47 (d, *J* = 6.0 Hz, 1H), 3.00 (d, *J* = 5.7 Hz, 1H), 2.78 (dd, *J* = 9.9, 7.3 Hz, 1H); ^13^C NMR (101 MHz, DMSO-*d*_6_) δ 176.18, 174.57, 174.10, 174.06, 168.68, 167.87, 153.13, 152.58, 145.27, 137.10, 135.98, 135.43, 134.50, 133.47, 132.44, 125.81, 124.78, 121.44, 118.15, 111.68, 107.49, 106.78, 86.80, 79.46, 76.26, 75.62; Chemical Formula: C_23_H_17_Cl_2_N_3_O_5_S_2_; LCMS (*m*/*z*): 551.40 [M + H]^+^, Elemental Analysis: [Calculated: C, 50.19; H, 3.11; N, 7.63; S, 11.65; Found: C, 50.18; H, 3.09; N, 7.659; S, 11.60].

### 2-((3*S*,6′*S*,7′*S*,7a′*S*)-7'-(4-Chlorophenyl)-5-fluoro-2,2″,4″-trioxo-7′,7a′-dihydro-1′*H*,3′*H*-dispiro [indoline-3,5′-pyrrolo [1,2-*c*]thiazole-6′,5″-thiazolidin]-3″-yl)acetic acid 5d

The 5-fluoro-isatin **2c** (82.5 mg) and **4b** (156.0 mg) were utilized according to the general method, and the spiro-compound **5d** was obtained in 85% yield.

^1^H NMR (400 MHz, DMSO-*d*_6_) δ 11.06 (s, 1H), 7.50–7.37 (m, 5H), 7.17 (td, *J* = 8.9, 2.7 Hz, 1H), 7.07 (dd, *J* = 9.1, 2.7 Hz, 1H), 6.88 (dd, *J* = 8.5, 4.6 Hz, 1H), 4.89–4.78 (m, 1H), 4.23–4.13 (m, 2H), 4.05 (d, *J* = 17.4 Hz, 1H), 3.82 (d, *J* = 6.0 Hz, 1H), 3.47 (d, *J* = 6.1 Hz, 1H), 3.00 (dd, *J* = 9.6, 5.8 Hz, 1H);^13^C NMR (101 MHz, DMSO-*d*_6_) δ 176.22, 174.50, 168.78, 167.82, 164.98, 160.63, 157.47, 139.96, 137.26, 136.95, 135.39, 134.18, 133.47, 132.30, 129.84, 129.38, 124.15, 121.31, 116.73, 112.07, 110.97, 84.14, 79.14, 76.48, 75.81, 66.90, 56.67, 47.04, 42.99, 40.79, 40.68, 40.59, 40.47, 40.37, 40.26, 40.16, 40.05, 39.84, 39.63, 39.52, 39.43, 32.17, 31.31, 31.21; Chemical Formula: C_23_H_17_ClFN_3_O_5_S_2_; LCMS (*m*/*z*): 534.94 [M + H]^+^, Elemental Analysis: [Calculated: C, 51.74; H, 3.21; N, 7.87; S, 12.01; Found: C, 51.76; H, 3.23; N, 7.92; S, 12.00].

### 2-((3*S*,6′*S*,7′*S*,7a′*S*)-7'-(4-Chlorophenyl)-5-methyl-2,2″,4″-trioxo-7′,7a′-dihydro-1′*H*,3′*H*-dispiro [indoline-3,5′-pyrrolo [1,2-*c*]thiazole-6′,5″-thiazolidin]-3″-yl)acetic acid 5e

The 5-methyl-isatin **2d** (80.5 mg) and **4b** (156.0 mg) were utilized according to the general method, and the spiro-compound **5e** was obtained in 87% yield.

^1^H NMR (400 MHz, DMSO-*d*_6_) δ 10.93 (s, 1H), 7.50–7.38 (m, 5H), 7.16–7.04 (m, 2H), 6.76 (d, *J* = 8.0 Hz, 1H), 4.91–4.80 (m, 1H), 4.22–4.09 (m, 2H), 4.02 (d, *J* = 17.5 Hz, 1H), 3.85 (d, *J* = 5.9 Hz, 1H), 3.45 (d, *J* = 5.9 Hz, 1H), 3.01 (dd, *J* = 9.6, 5.7 Hz, 1H), 2.77 (d, *J* = 7.3 Hz, 0H); ^13^C NMR (101 MHz, DMSO-*d*_6_) δ 176.16, 174.99, 168.90, 167.84, 165.29, 141.27, 139.39, 135.86, 133.39, 132.20, 129.29, 127.99, 127.39, 126.84, 122.57, 112.70, 110.78, 95.65, 92.01, 89.01, 76.36, 75.94, 70.22, 70.09, 56.40, 47.04, 41.87, 40.68, 40.56, 40.47, 40.36, 40.26, 40.15, 40.05, 39.84, 39.63, 39.42, 32.28, 30.03, 18.89; Chemical Formula: C_24_H_20_ClN_3_O_5_S_2_; LCMS (*m*/*z*): 531.01 [M + H]^+^, Elemental Analysis: [Calculated: C, 54.39; H, 3.80; N, 7.93; S, 12.10; Found: C, 54.39; H, 3.80; N, 7.93; S, 12.10].

### 2-((3*S*,6′*S*,7′*S*,7a′*S*)-7'-(4-Chlorophenyl)-5-nitro-2,2″,4″-trioxo-7′,7a′-dihydro-1′*H*,3′*H*-dispiro [indoline-3,5′-pyrrolo [1,2-*c*]thiazole-6′,5″-thiazolidin]-3″-yl)acetic acid 5f

The 5-nitro-isatin **2e** (96.0 mg) and **4b** (156.0 mg) were utilized according to the general method, and the Spiro-compound **5f** was obtained in 82% yield.

^1^H NMR (400 MHz, DMSO-*d*_6_) δ 13.24 (s, 1H), 11.71 (s, 1H), 8.27 (dd, *J* = 8.8, 2.6 Hz, 1H), 8.13 (d, *J* = 2.7 Hz, 1H), 7.51–7.39 (m, 5H), 7.08 (d, *J* = 8.4 Hz, 1H), 4.85–4.74 (m, 1H), 4.25–4.13 (m, 2H), 4.01 (d, *J* = 17.5 Hz, 1H), 3.85 (d, *J* = 6.1 Hz, 1H), 3.49 (d, *J* = 6.0 Hz, 1H), 3.02 (dd, *J* = 9.6, 5.7 Hz, 1H); ^13^C NMR (101 MHz, DMSO-*d*_6_) δ 176.75, 174.20, 168.40, 167.73, 159.45, 149.95, 143.45, 135.24, 133.56, 132.43, 130.71, 129.02, 128.48, 124.17, 124.06, 123.25, 115.86, 113.97, 111.40, 111.26, 76.46, 75.17, 70.37, 56.26, 46.89, 40.71, 40.50, 40.29, 40.08, 39.87, 39.66, 39.46, 32.22; Chemical Formula: C_23_H_17_ClN_4_O_7_S_2_; LCMS (*m*/*z*): 561.93 [M + H]^+^, Elemental Analysis: [Calculated: C, 49.24; H, 3.05; N, 9.99; S, 11.43; Found: C, 49.26; H, 3.10; N, 10.9; S, 11.45].

### 2-((3*S*,6′*S*,7′S,7a′*S*)-5-bromo-7'-(4-chlorophenyl)-2,2″,4″-trioxo-7′,7a′-dihydro-1′*H*,3′*H*-dispiro [indoline-3,5′-pyrrolo [1,2-*c*]thiazole-6′,5″-thiazolidin]-3″-yl)acetic acid 5g

The 5-bromo-isatin **2f** (96.0 mg) and **4b** (156.0 mg) were utilized according to the general method, and the final compound **5g** was obtained in 86% yield.

^1^H NMR (400 MHz, DMSO-*d*_6_) δ 13.34 (s, 1H), 11.17 (s, 1H), 7.54–7.35 (m, 7H), 6.84 (d, *J* = 8.6 Hz, 1H), 4.80 (q, *J* = 7.7 Hz, 1H), 4.26–4.14 (m, 2H), 3.97 (d, *J* = 17.0 Hz, 1H), 3.77 (d, *J* = 6.0 Hz, 1H), 3.47 (d, *J* = 6.3 Hz, 1H), 2.99 (dd, *J* = 9.9, 5.5 Hz, 1H);^13^C NMR (101 MHz, DMSO-*d*_6_) δ 175.80, 174.40, 168.58, 167.67, 143.04, 135.36, 134.42, 134.01, 133.50, 132.30, 130.58, 130.39, 129.21, 124.90, 114.83, 113.06, 110.51, 76.67, 75.52, 68.59, 56.28, 56.20, 49.23, 46.98, 44.35, 40.71, 40.50, 40.29, 40.09, 39.88, 39.67, 39.54, 39.46, 32.86; Chemical Formula: C_23_H_17_BrClN_3_O_5_S_2_; LCMS (*m*/*z*): 595.97 [M + H]^+^, Elemental Analysis: [Calculated: C, 49.24; H, 3.05; N, 9.99; S, 11.43; Found: C, 46.50; H, 3.00; N, 7.12; S, 10.83].

### Methyl (2-((3*S*,6′*S*,7′*S*,7a′*S*)-7'-(4-bromophenyl)-6-chloro-2,2″,4″-trioxo-7′,7a′-dihydro-1′*H*,3′*H*-dispiro [indoline-3,5′-pyrrolo [1,2-*c*]thiazole-6′,5″-thiazolidin]-3″-yl)acetyl)valinate 5l

The 6-chloro-isatin **2b** (90.5 mg) and **4c** (235.0 mg) were utilized according to the general method, and the spiro-compound **5l** was obtained in 80% yield.

^1^H NMR (400 MHz, DMSO-*d*_6_) δ 11.18 (d, *J* = 3.9 Hz, 1H), 8.51 (dd, *J* = 8.8, 5.3 Hz, 1H), 7.71–7.58 (m, 1H), 7.50–7.36 (m, 4H), 7.26 (dd, *J* = 8.4, 4.0 Hz, 1H), 7.04 (dd, *J* = 8.1, 2.2 Hz, 1H), 6.91 (s, 1H), 4.82 (dt, *J* = 13.1, 5.2 Hz, 1H), 4.18 (td, *J* = 9.3, 4.2 Hz, 2H), 4.07 (d, *J* = 6.1 Hz, 1H), 4.05 (s, 0H), 3.82 (dd, *J* = 6.0, 2.2 Hz, 1H), 3.69–3.61 (m, 3H), 3.46 (dd, *J* = 6.1, 3.2 Hz, 1H), 3.01 (dd, *J* = 9.6, 5.8 Hz, 1H), 2.84–2.73 (m, 1H), 2.16–2.09 (m, 1H), 0.86 (ddd, *J* = 18.5, 6.9, 4.2 Hz, 7H); ^13^C NMR (101 MHz, DMSO-*d*_6_) δ 207.05, 176.20, 174.73, 174.60, 172.19, 168.65, 166.00, 165.16, 165.07, 145.35, 135.99, 135.61, 133.42, 132.25, 129.28, 121.57, 106.62, 106.31, 76.29, 76.20, 75.59, 75.52, 57.92, 55.53, 52.37, 47.06, 43.66, 40.67, 40.58, 40.46, 40.37, 40.25, 40.16, 40.04, 39.95, 39.83, 39.63, 39.42, 32.27, 31.37, 31.26, 31.15, 31.04, 30.81, 19.40, 18.46; Chemical Formula: C_29_H_28_BrClN_4_O_6_S_2_; LCMS (*m*/*z*): 709.01 [M + H]^+^, Elemental Analysis: [Calculated: C, 49.19; H, 3.99; N, 7.91; 56; S, 9.06; Found: C, 49.16; H, 4.01; N, 7.99; 56; S, 9.02].

### Methyl (2-((3*S*,6′*S*,7′*S*,7a′*S*)-7'-(4-bromophenyl)-5-fluoro-2,2″,4″-trioxo-7′,7a′-dihydro-1′*H*,3′*H*-dispiro [indoline-3,5′-pyrrolo [1,2-*c*]thiazole-6′,5″-thiazolidin]-3″-yl)acetyl)valinate 5m

The 5-Fluoro-isatin **2c** (82.5 mg) and **4c** (235.0 mg) were utilized according to the general method, and the spiro-compound **5m** was obtained in 82% yield.

^1^H NMR (400 MHz, DMSO-*d*_6_) δ 11.06 (s, 1H), 8.53 (t, *J* = 9.0 Hz, 1H), 7.72–7.58 (m, 1H), 7.51–7.37 (m, 4H), 7.19 (td, *J* = 8.9, 2.7 Hz, 1H), 7.02 (dt, *J* = 8.8, 3.0 Hz, 1H), 6.89 (dd, *J* = 8.7, 4.5 Hz, 1H), 4.83 (d, *J* = 7.6 Hz, 1H), 4.24–4.05 (m, 4H), 3.81 (d, *J* = 6.0 Hz, 1H), 3.65 (d, *J* = 10.5 Hz, 3H), 3.46 (s, 1H), 2.99 (dd, *J* = 9.6, 5.7 Hz, 1H), 2.15–2.07 (m, 2H), 0.92–0.80 (m, 7H);^13^C NMR (101 MHz, DMSO-*d*_6_) δ 207.11, 192.69, 176.19, 174.76, 172.15, 170.54, 168.73, 165.73, 165.14, 140.04, 135.57, 133.42, 131.58, 129.60, 125.81, 123.36, 92.33, 79.20, 76.28, 75.75, 59.09, 58.38, 55.59, 52.35, 52.30, 46.85, 40.80, 40.68, 40.59, 40.48, 40.38, 40.27, 40.17, 40.06, 39.85, 39.72, 39.64, 39.43, 31.40, 31.30, 31.20, 31.10; Chemical Formula: C_29_H_28_BrFN_4_O_6_S_2_; LCMS (*m*/*z*): 692.59 [M + H]^+^, Elemental Analysis: [Calculated: C, 50.37; H, 4.08; N, 8.10; S, 9.27; Found: C, 50.36; H, 4.09; N, 8.07; S, 9.29].

### Methyl (2-((3*S*,6′*S*,7′*S*,7a′*S*)-6-chloro-7'-(4-chlorophenyl)-2,2″,4″-trioxo-7′,7a′-dihydro-1′H,3′H-dispiro [indoline-3,5′-pyrrolo [1,2-*c*]thiazole-6′,5″-thiazolidin]-3″-yl)acetyl)alaninate 5n

The 6-chloro-isatin **2b** (90.5 mg) and **4d** (221.5 mg) were utilized according to the general method, and the spiro-compound **5n** was obtained in 84% yield.

^1^H NMR (400 MHz, DMSO-*d*_6_) δ 11.18 (d, *J* = 5.0 Hz, 1H), 8.63 (t, *J* = 6.8 Hz, 1H), 7.72–7.59 (m, 1H), 7.47 (d, *J* = 8.1 Hz, 2H), 7.41 (d, *J* = 8.2 Hz, 2H), 7.25 (t, *J* = 8.9 Hz, 1H), 7.05 (t, *J* = 9.4 Hz, 1H), 6.90 (s, 1H), 4.82 (q, *J* = 7.9, 7.4 Hz, 1H), 4.35–4.23 (m, 1H), 4.21–4.12 (m, 1H), 4.10–3.91 (m, 2H), 3.82 (d, *J* = 6.0 Hz, 1H), 3.64 (d, *J* = 6.7 Hz, 3H), 3.49–3.41 (m, 1H), 3.01 (t, *J* = 7.7 Hz, 1H), 1.28 (q, *J* = 7.1 Hz, 3H); ^13^C NMR (101 MHz, DMSO-*d*_6_) δ 181.16, 176.20, 174.37, 172.16, 169.71, 167.74, 164.67, 151.40, 143.74, 135.98, 135.63, 135.05, 133.41, 132.31, 131.02, 126.13, 125.81, 123.05, 112.07, 94.30, 84.51, 76.27, 75.58, 59.25, 54.52, 52.58, 48.11, 46.69, 40.71, 40.63, 40.51, 40.30, 40.09, 39.88, 39.67, 39.46, 17.74; Chemical Formula: C_27_H_24_Cl_2_N_4_O_6_S_2_; LCMS (*m*/*z*): 636.59 [M + H]^+^, Elemental Analysis: [Calculated: C, 51.03; H, 3.81; N, 8.82; S, 10.09; Found: C, 51.03; H, 3.81; N, 8.82; S, 10.09].

### Methyl 4-(2-((3*S*,6′*S*,7′*S*,7a′*S*)-7'-(4-bromophenyl)-6-chloro-2,2″,4″-trioxo-7′,7a′-dihydro-1′*H*,3′*H*-dispiro [indoline-3,5′-pyrrolo [1,2-*c*]thiazole-6′,5″-thiazolidin]-3″-yl)acetamido)butanoate 5o

The 6-chloro-isatin **2b** (90.5 mg) and **4e** (228.5 mg) were utilized according to the general method, and the spiro-compound **5o** was obtained in 81% yield.

^1^H NMR (400 MHz, DMSO-*d*_6_) δ 11.18 (s, 1H), 8.16 (t, *J* = 5.6 Hz, 1H), 7.68 (d, *J* = 8.6 Hz, 0H), 7.63 (d, *J* = 8.7 Hz, 0H), 7.52–7.38 (m, 5H), 7.26 (d, *J* = 8.5 Hz, 1H), 7.06 (dd, *J* = 8.4, 2.3 Hz, 1H), 6.90 (d, *J* = 2.1 Hz, 1H), 4.82 (q, *J* = 7.7, 7.2 Hz, 1H), 4.29–4.12 (m, 2H), 4.07–3.77 (m, 3H), 3.45 (d, *J* = 6.1 Hz, 1H), 3.13–3.00 (m, 3H), 3.05–2.96 (m, 1H), 2.77 (dd, *J* = 9.8, 7.6 Hz, 1H), 1.72–1.57 (m, 3H); ^13^C NMR (101 MHz, DMSO-*d*_6_) δ 176.22, 174.78, 173.62, 173.08, 168.74, 167.22, 165.52, 164.72, 145.38, 136.00, 135.64, 133.40, 132.33, 129.46, 129.42, 123.04, 121.61, 112.52, 112.22, 76.19, 75.48, 70.60, 63.64, 56.11, 51.86, 50.22, 48.78, 47.03, 43.86, 40.71, 40.63, 40.50, 40.47, 40.29, 40.09, 39.88, 39.67, 39.54, 39.46, 38.62, 32.26, 31.11, 24.92; Chemical Formula: C_28_H_26_BrClN_4_O_6_S_2_; LCMS (*m*/*z*): 695.02 [M + H]^+^, Elemental Analysis: [Calculated: C, 48.46; H, 3.78; N, 8.07; S, 9.24; Found: C, 48.49; H, 3.80; N, 8.11; S, 9.25].

### Crystal structure determination

The technical protocol and data manipulation software details (Rikagu Oxford Diffraction CrysAlisPro, 2020; Sheldrick. 2015; Hübschle et al., 2011) are available in the Supplementary Material S1.

### Biological investigations

The methods for the Cytotoxic activity (Mosmann, 1983; Nafie et al., 2020a); EGFR/CDK-2 enzyme inhibition (Nafie et al., 2022a); Flow cytometry using Annexin V/PI staining; Gene expression analysis using RT-PCR (Nafie et al., 2022b); are amended in the Supplementary Material S1.

### Molecular docking and molecular dynamic simulation

The protcol for the Molecular docking and Molecular dynamic simulation are provided in in the Supplementary Material S1 ((Wood et al., 2019; Stamos et al., 2002; Chemical Computing Group, 2013; Case et al., 2023; Khalil et al., 2019; Roe and Cheatham, 2013).

## Conclusion

In conclusion, the synthesized compounds, particularly **5g**, **5l**, and **5n**, exhibited remarkable cytotoxicity against cancer cells, with noteworthy potency against both MCF-7 and MDA-MB-231 cells. Additionally, these compounds demonstrated promising inhibitory activities against EGFR and CDK-2, showcasing their potential as dual inhibitors. The RT-PCR results further confirmed their impact on promoting apoptotic cell death by modulating the expression of key pro-apoptotic and anti-apoptotic genes. Molecular docking and dynamic simulations provided insights into the binding modes of these compounds within the active sites of EGFR and CDK-2, reinforcing their potential as therapeutic agents. Overall, this comprehensive study underscores the multifaceted potential of these compounds in cancer treatment, warranting further investigation and development.

## Data Availability

The original contributions presented in the study are included in the article/Supplementary Material, further inquiries can be directed to the corresponding authors.

## References

[B1] Abd Alhameed R. Almarhoon Z. Bukhari S. I. El-Faham A. de la Torre B. G. Albericio F. (2020). Synthesis and antimicrobial activity of a new series of thiazolidine-2,4-diones carboxamide and amino acid derivatives. Molecules 25, 105. 10.3390/molecules25010105 PMC6983134 31892119

[B2] Al-Jassas R. M. Islam M. S. Al-Majid A. M. Nafie M. S. Haukka M. Rahman A. M. (2023). Synthesis and SARs study of novel spiro-oxindoles as potent antiproliferative agents with CDK-2 inhibitory activities. Arch. Pharm. 356, e2300185. 10.1002/ardp.202300185 37253118

[B3] Al-Rashood S. T. Hamed A. R. Hassan G. S. Alkahtani H. M. Almehizia A. A. Alharbi A. (2020). Anti-tumor properties of certain spirooxindoles towards hepatocellular carcinoma endowed with antioxidant activity. J. Enzyme Inhib. Med. Chem. 35 (1), 831–839. 10.1080/14756366.2020.1743281 32208781 PMC7144320

[B4] Bacher N. Tiefenthaler M. Sturm S. Stuppner H. Ausserlechner M. J. Kofler R. (2006). Oxindole alkaloids from Uncaria tomentosa induce apoptosis in proliferating, G0/G1-arrested and bcl-2-expressing acute lymphoblastic leukaemia cells. Br. J. Haematol. 132 (5), 615–622. 10.1111/j.1365-2141.2005.05907.x 16445836

[B5] Barakat A. Alshahrani S. Al-Majid A. M. Alamary A. S. Haukka M. Abu-Serie M. M. (2023). New spiro-indeno[1,2-b]quinoxalines clubbed with benzimidazole scaffold as CDK2 inhibitors for halting non-small cell lung cancer; stereoselective synthesis, molecular dynamics and structural insights. J. Enzyme Inhib. Med. Chem. 38 (1), 2281260. 10.1080/14756366.2023.2281260 37994663 PMC11003489

[B6] Barakat A. Haukka M. Soliman S. M. Ali M. Al-Majid A. M. El-Faham A. (2021). Straightforward regio- and diastereoselective synthesis, molecular structure, intermolecular interactions and mechanistic study of spirooxindole-engrafted rhodanine analogs. Molecules 26, 7276. 10.3390/molecules26237276 34885853 PMC8658983

[B7] Bramson H. N. Corona J. Davis S. T. Dickerson S. H. Edelstein M. Frye S. V. (2001). Oxindole-based inhibitors of cyclin-dependent kinase 2 (CDK2): design, synthesis, enzymatic activities, and X-ray crystallographic analysis. J. Med. Chem. 44 (25), 4339–4358. 10.1021/jm010117d 11728181

[B8] Budovská M. Tischlerová V. Mojžiš J. Kozlov O. Gondová T. (2020). An alternative approach to the synthesis of anti-cancer molecule Spirobrassinin and its 2′-amino analogues. Monatsh. Chem. 151 (1), 63–77. 10.1007/s00706-019-02528-x

[B9] Case D. A. A. Belfon K. Ben-Shalom I. Y. Berryman J. T. Brozell S. R. Cerutti D. S. (2023). Amber 2023. San Francisco: University of California.

[B10] Chemical Computing Group (2013). Molecular operating environment (MOE). Sherbooke St. West, Montreal, QC, Canada.

[B11] Cui C. B. Kakeya H. Osada H. (1996). Spirotryprostatin B, a novel mammalian cell cycle inhibitor produced by Aspergillus fumigatus. J. Antibiot. 49 (8), 832–835. 10.7164/antibiotics.49.832 8823522

[B12] Ding K. Lu Y. Nikolovska-Coleska Z. Qiu S. Ding Y. Gao W. (2005). Structure-based design of potent non-peptide MDM2 inhibitors. J. Am. Chem. Soc. 127 (29), 10130–10131. 10.1021/ja051147z 16028899

[B13] Edmondson S. Danishefsky S. J. Sepp-Lorenzino L. Rosen N. (1999). Total synthesis of spirotryprostatin A, leading to the discovery of some biologically promising analogues. J. Am. Chem. Soc. 121 (10), 2147–2155. 10.1021/ja983788i

[B14] Galliford C. V. Scheidt K. A. (2007). Pyrrolidinyl‐spirooxindole natural products as inspirations for the development of potential therapeutic agents. Angew. Chem. Int. Ed. Engl. 46 (46), 8748–8758. 10.1002/anie.200701342 17943924

[B15] García Giménez D. García Prado E. Sáenz Rodríguez T. Fernández Arche A. De La Puerta R. (2010). Cytotoxic effect of the pentacyclic oxindole alkaloid mitraphylline isolated from Uncaria tomentosa bark on human ewing’s sarcoma and breast cancer cell lines. Planta Med. 76 (2), 133–136. 10.1055/s-0029-1186048 19724995

[B16] Golsteyn R. M. (2005). Cdk1 and Cdk2 complexes (cyclin dependent kinases) in apoptosis: a role beyond the cell cycle. Cancer Lett. 217 (2), 129–138. 10.1016/j.canlet.2004.08.005 15617830

[B17] Guo K. Fang T. Wang J. Wu A. A. Wang Y. Jiang J. (2014). Two new spirooxindole alkaloids from rhizosphere strain streptomyces sp. Xzqh-9. Bioorg. Med. Chem. Lett. 24 (21), 4995–4998. 10.1016/j.bmcl.2014.09.026 25278238

[B18] Hübschle C. B. Sheldrick G. M. Dittrich B. (2011). ShelXle: a Qt graphical user interface for SHELXL. J. Appl. Cryst. 44, 1281–1284. 10.1107/S0021889811043202 22477785 PMC3246833

[B19] Islam F. Dehbia Z. Zehravi M. Das R. Sivakumar M. Krishnan K. (2023). Indole alkaloids from marine resources: understandings from therapeutic point of view to treat cancers. Chem. Biol. Interact. 383, 110682. 10.1016/j.cbi.2023.110682 37648047

[B20] Khalil R. Ashraf S. Khalid A. Ul-Haq Z. (2019). Exploring novel N-myristoyltransferase inhibitors: a molecular dynamics simulation approach. ACS omega 15 (9), 13658–13670. 10.1021/acsomega.9b00843 PMC6714517 31497683

[B21] Luk K.-C. Simcox M. E. Schutt A. Rowan K. Thompson T. Chen Y. (2004). A new series of potent oxindole inhibitors of CDK2. Bioorg. Med. Chem. Lett. 14 (4), 913–917. 10.1016/j.bmcl.2003.12.009 15012993

[B22] Marti C. Carreira E. M. (2003). Construction of Spiro [pyrrolidine‐3, 3′‐oxindoles]− recent applications to the synthesis of oxindole alkaloids. EurJOC 2003 (12), 2209–2219. 10.1002/ejoc.200300050

[B23] Mosmann T. (1983). Rapid colorimetric assay for cellular growth and survival: Application to proliferation and cytotoxicity assays. J. Immunol. Methods. 65, 55–63. 10.1016/0022-1759(83)90303-4 6606682

[B24] Nafie M. S. Amer A. M. Mohamed A. K. Tantawy E. S. (2020b). Discovery of novel pyrazolo[3,4-b]pyridine scaffold-based derivatives as potential PIM-1 kinase inhibitors in breast cancer MCF-7 cells. Bioorg. Med. Chem. 28, 115828. 10.1016/j.bmc.2020.115828 33166925

[B25] Nafie M. S. Arafa K. Sedky N. K. Alakhdar A. A. Arafa R. K. (2020a). Triaryl dicationic DNA minor-groove binders with antioxidant activity display cytotoxicity and induce apoptosis in breast cancer. Chem. Biol. Interact. 324, 109087. 10.1016/j.cbi.2020.109087 32294457

[B26] Nafie M. S. Boraei A. T. A. (2022). Exploration of novel VEGFR2 tyrosine kinase inhibitors via design and synthesis of new alkylated indolyl-triazole Schiff bases for targeting breast cancer. Bioorg. Chem. 122, 105708. 10.1016/j.bioorg.2022.105708 35290929

[B27] Nafie M. S. Elghazawy N. H. Owf S. M. Arafa K. Abdel-Rahman M. A. Arafa R. K. (2022b). Control of ER-positive breast cancer by ERα expression inhibition, apoptosis induction, cell cycle arrest using semisynthetic isoeugenol derivatives. Chem. Biol. Interact. 351, 109753. 10.1016/j.cbi.2021.109753 34801536

[B28] Nafie M. S. Kishk S. M. Mahgoub S. Amer A. M. (2022a). Quinoline-based thiazolidinone derivatives as potent cytotoxic and apoptosis-inducing agents through EGFR inhibition. Chem. Biol. Drug Des. 99, 547–560. 10.1111/cbdd.13997 34873844

[B29] Rikagu Oxford Diffraction CrysAlisPro (2020) CrysAlisPro, Yarnton, Oxfordshire, England: Rikagu Oxford Diffraction inc.,

[B30] Roe D. R. Cheatham T. E. III (2013). PTRAJ and CPPTRAJ: software for processing and analysis of molecular dynamics trajectory data. J. Chem. theory Comput. 9 (7), 3084–3095. 10.1021/ct400341p 26583988

[B31] Sheldrick G. M. (2015). Crystal structure refinement with SHELXL. Acta Cryst. C71, 3–8. 10.1107/S2053229614024218 PMC4294323 25567568

[B32] Sheldrick G. M. (2015). SHELXT – integrated space-group and crystal-structure determination. Acta Cryst. A71, 3–8. 10.1107/S2053273314026370 PMC4283466 25537383

[B33] Stamos J. Sliwkowski M. X. Eigenbrot C. (2002). Structure of the epidermal growth factor receptor kinase domain alone and in complex with a 4-anilinoquinazoline inhibitor. J. Biol. Chem. 277 (48), 46265–46272. 10.1074/jbc.M207135200 12196540

[B34] Sung H. Ferlay J. Siegel R. L. Laversanne M. Soerjomataram I. Jemal A. (2021). Global cancer statistics 2020: GLOBOCAN estimates of incidence and mortality worldwide for 36 cancers in 185 countries. Ca. Cancer J. Clin. 71 (3), 209–249. 10.3322/caac.21660 33538338

[B35] Tadesse S. Caldon E. C. Tilley W. Wang S. (2018). Cyclin-dependent kinase 2 inhibitors in cancer therapy: an update. J. Med. Chem. 62 (9), 4233–4251. 10.1021/acs.jmedchem.8b01469 30543440

[B36] Venkanna A. Subedi L. Teli M. K. Lama P. D. Nangunuri B. G. Lee S.-Y. (2020). Positioning of an unprecedented spiro [5.5] undeca ring system into kinase inhibitor space. Sci. Rep. 10 (1), 21265. 10.1038/s41598-020-78158-9 33277542 PMC7719162

[B37] Wang B. Peng F. Huang W. Zhou J. Zhang N. Sheng J. (2020). Rational drug design, synthesis, and biological evaluation of novel chiral tetrahydronaphthalene-fused spirooxindole as MDM2-CDK4 dual inhibitor against glioblastoma. Acta Pharm. Sin. B 10 (8), 1492–1510. 10.1016/j.apsb.2019.12.013 32963945 PMC7488488

[B38] Wood D. J. Lopez-Fernandez J. D. Knight L. E. Al-Khawaldeh I. Gai C. Lin S. (2019). FragLites—minimal, halogenated fragments displaying pharmacophore doublets. An efficient approach to druggability assessment and hit generation. J. Med. Chem. 62 (7), 3741–3752. 10.1021/acs.jmedchem.9b00304 30860382

[B39] World Cancer Research Fund (2021). Cancer facts and figures 2021. London, United Kingdom: World Cancer Research Fund International, 1–4.

[B40] Yu B. Yu D. Q. Liu H. M. (2015). Spirooxindoles: promising scaffolds for anti-cancer agents. Eur. J. Med. Chem. 97, 673–698. 10.1016/j.ejmech.2014.06.056 24994707

[B41] Yu Q. Guo P. Jian J. Chen Y. Xu J. (2018). Nine-step total synthesis of (-)-Strychnofoline. Chem. Commun. 54 (9), 1125–1128. 10.1039/c7cc08938d 29334094

[B42] Yuenyongsawad S. Bunluepuech K. Wattanapiromsakul C. Tewtrakul S. (2013). Anti-cancer activity of compounds from Bauhinia strychnifolia stem. J. Ethnopharmacol. 150 (2), 765–769. 10.1016/j.jep.2013.09.025 24120967

[B43] Zhou L. M. Qu R. Y. Yang G. F. (2020). An overview of spirooxindole as a promising scaffold for novel drug discovery. Expert Opin. Drug Discov. 15 (5), 603–625. 10.1080/17460441.2020.1733526 32106717

