# Supplementary material for: Exploring pyrrolidinyl-spirooxindole natural products as promising platforms for the synthesis of novel spirooxindoles as EGFR/CDK2 inhibitors for halting breast cancer cells
Source: Front Chem. 2024 Feb 29;12:1364378. doi: 10.3389/fchem.2024.1364378 (PMC10937419; doi:10.3389/fchem.2024.1364378)

checkCIF/PLATON report

Structure factors have been supplied for datablock(s) ab1194

THIS REPORT IS FOR GUIDANCE ONLY. IF USED AS PART OF A REVIEW PROCEDURE FOR PUBLICATION, IT SHOULD NOT REPLACE THE EXPERTISE OF AN EXPERIENCED CRYSTALLOGRAPHIC REFEREE.

No syntax errors found. CIF dictionary Interpreting this report

Datablock: ab1194

```
Bond precision:  C-C = 0.0034 A          Wavelength=1.54184

Cell:            a=11.5475(3)    b=15.7550(4)       c=14.6493(3)
                 alpha=90        beta=104.655(2)    gamma=90
Temperature:     120 K
```

	Calculated	Reported
Volume	2578.45(11)	2578.45(11)
Space group	P 21/n	P 21/n
Hall group	-P 2yn	-P 2yn
Moiety formula	C24 H20 Cl N3 O5 S2, C H4 O	C25 H24 Cl N3 O6 S2
Sum formula	C25 H24 Cl N3 O6 S2	C25 H24 Cl N3 O6 S2
Mr	562.04	562.04
Dx,g cm-3	1.448	1.448
Z	4	4
Mu (mm-1)	3.225	3.225
F000	1168.0	1168.0
F000’	1175.36	
h,k,lmax	14,19,18	14,19,18
Nref	5445	5413
Tmin,Tmax	0.583,0.846	0.726,1.000
Tmin’	0.423	

```
Correction method= # Reported T Limits: Tmin=0.726 Tmax=1.000
AbsCorr = GAUSSIAN

Data completeness= 0.994          Theta(max)= 76.853

R(reflections)= 0.0487( 5038)     wR2(reflections)= 0.1291( 5413)

S = 1.099                  Npar= 344
```

The following ALERTS were generated. Each ALERT has the format
test-name_ALERT_alert-type_alert-level.
Click on the hyperlinks for more details of the test.

Alert level C

```
PLAT906_ALERT_3_C Large K Value in the Analysis of Variance ......      3.142 Check
PLAT911_ALERT_3_C Missing FCF Refl Between Thmin & STh/L=    0.600          2 Report
PLAT975_ALERT_2_C Check Calcd Resid. Dens.  0.85A   From O1              0.41 eA-3
```

Alert level G

```
PLAT007_ALERT_5_G Number of Unrefined Donor-H Atoms ..............          1 Report
PLAT042_ALERT_1_G Calc. and Reported MoietyFormula Strings  Differ     Please Check
PLAT793_ALERT_4_G Model has Chirality at C9          (Centro SPGR)          R Verify
PLAT793_ALERT_4_G Model has Chirality at C12         (Centro SPGR)          S Verify
PLAT793_ALERT_4_G Model has Chirality at C13         (Centro SPGR)          R Verify
PLAT793_ALERT_4_G Model has Chirality at C14         (Centro SPGR)          R Verify
PLAT883_ALERT_1_G No Info/Value for _atom_sites_solution_primary .     Please Do !
PLAT912_ALERT_4_G Missing # of FCF Reflections Above STh/L=  0.600         29 Note
PLAT941_ALERT_3_G Average HKL Measurement Multiplicity ...........        3.8 Low
PLAT965_ALERT_2_G The SHELXL WEIGHT Optimisation has not Converged     Please Check
PLAT978_ALERT_2_G Number C-C Bonds with Positive Residual Density.          6 Info
```

0 **ALERT level A** = Most likely a serious problem - resolve or explain
0 **ALERT level B** = A potentially serious problem, consider carefully
3 **ALERT level C** = Check. Ensure it is not caused by an omission or oversight
11 **ALERT level G** = General information/check it is not something unexpected

2 ALERT type 1 CIF construction/syntax error, inconsistent or missing data
3 ALERT type 2 Indicator that the structure model may be wrong or deficient
3 ALERT type 3 Indicator that the structure quality may be low
5 ALERT type 4 Improvement, methodology, query or suggestion
1 ALERT type 5 Informative message, check

It is advisable to attempt to resolve as many as possible of the alerts in all categories. Often the minor alerts point to easily fixed oversights, errors and omissions in your CIF or refinement strategy, so attention to these fine details can be worthwhile. In order to resolve some of the more serious problems it may be necessary to carry out additional measurements or structure refinements. However, the purpose of your study may justify the reported deviations and the more serious of these should normally be commented upon in the discussion or experimental section of a paper or in the "special_details" fields of the CIF. checkCIF was carefully designed to identify outliers and unusual parameters, but every test has its limitations and alerts that are not important in a particular case may appear. Conversely, the absence of alerts does not guarantee there are no aspects of the results needing attention. It is up to the individual to critically assess their own results and, if necessary, seek expert advice.

Publication of your CIF in IUCr journals

A basic structural check has been run on your CIF. These basic checks will be run on all CIFs submitted for publication in IUCr journals (*Acta Crystallographica*, *Journal of Applied Crystallography*, *Journal of Synchrotron Radiation*); however, if you intend to submit to *Acta Crystallographica Section C* or *E* or *IUCrData*, you should make sure that full publication checks are run on the final version of your CIF prior to submission.

Publication of your CIF in other journals

Please refer to the *Notes for Authors* of the relevant journal for any special instructions relating to CIF submission.

PLATON version of 05/12/2020; check.def file version of 05/12/2020

Datablock ab1194 - ellipsoid plot

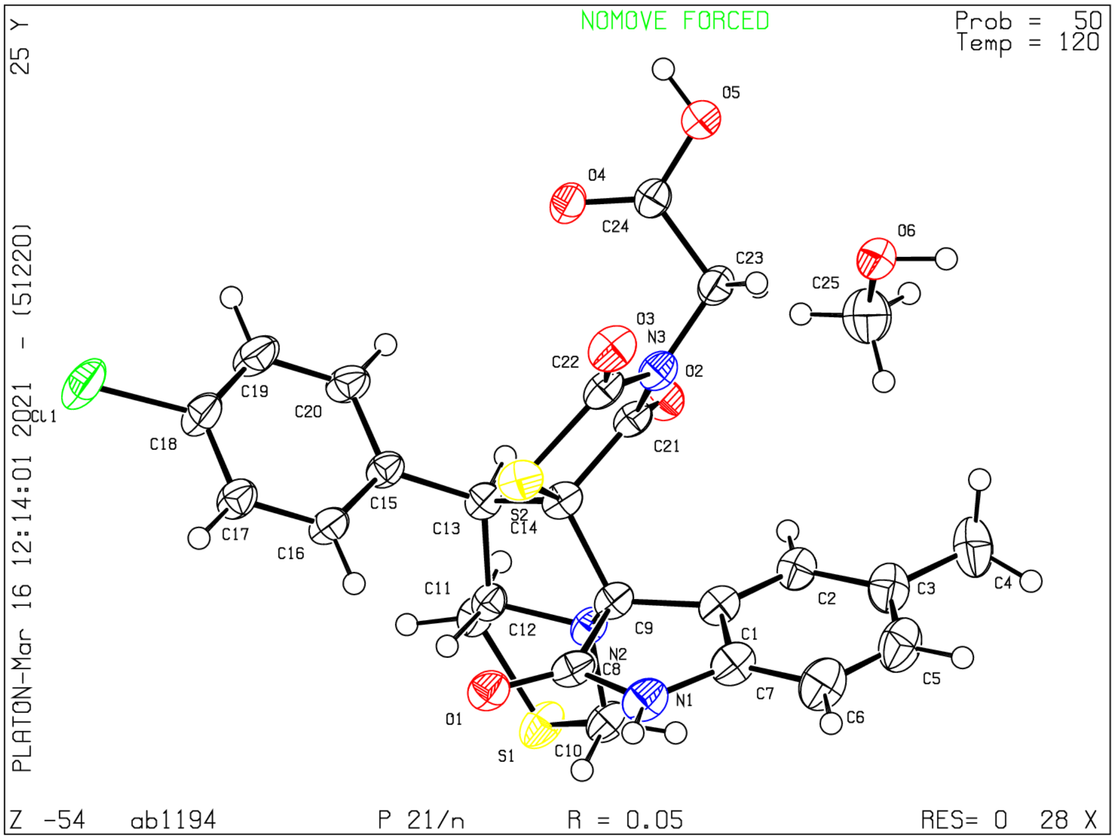

Supplement: Supplementary file 1 [file DataSheet1.PDF]
