# Supplementary material for: Exploring pyrrolidinyl-spirooxindole natural products as promising platforms for the synthesis of novel spirooxindoles as EGFR/CDK2 inhibitors for halting breast cancer cells
Source: Front Chem. 2024 Feb 29;12:1364378. doi: 10.3389/fchem.2024.1364378 (PMC10937419; doi:10.3389/fchem.2024.1364378)

**SUPPORTING MATERIALS**

**Exploring Pyrrolidinyl-Spirooxindole Natural Products as Promising Platforms for the Synthesis of Novel Spirooxindoles as CDK2 Inhibitors for Halting Breast Cancer Cells**

**Mohamed S. Nafie^1, 2^ Abdullah Mohammed Al-Majid,^3^ M. Ali,^3^ Abdulmajeed Abdullah Alayyaf^3^, Matti Haukka,^4^ Sajda Ashraf,^5^ Zaheer Ul-Haq, ^5^ Ayman El-Faham****, ^6,*^and Assem Barakat^3,*^**

1. Department of Chemistry, College of Sciences, University of Sharjah, Sharjah (P.O. Box 27272), United Arab Emirates.
2. Chemistry Department, Faculty of Science, Suez Canal University, Ismailia, 41522, Egypt. Email: mohamed_nafie@science.suez.edu.eg (M.S.N.).
3. Department of Chemistry, College of Science, King Saud University, P. O. Box 2455, Riyadh 11451, Saudi [Arabia. amajid@ksu.edu.sa](mailto:Arabia.%20Maly.c@ksu.edu.sa%20(M.A.);%20amajid@ksu.edu.sa) (A.M.A.); maly.c@ksu.edu.sa (M.A.).
4. Department of Chemistry, University of Jyväskylä, P.O. Box 35, FI-40014 Jyväskylä, Finland. matti.o.haukka@jyu.fi (M.H.).
5. Dr. Panjwani Center for Molecular medicine and Drug Research, International Center for Chemical and Biological Sciences, University of Karachi, Karachi-75270, Pakistan. [sajda.ashraf@yahoo.com](mailto:sajda.ashraf@yahoo.com) (S.A.); zaheer_qasmi@hotmail.com (Z.U.-H.).
6. Department of Chemistry, Faculty of Science, Alexandria University, P.O. Box 426, Ibrahimia, Alexandria 21321, Egypt. [ayman.elfaham@alexu.edu.eg](mailto:ayman.elfaham@alexu.edu.eg) (A.E.-F).

* Correspondence: Emails: [ayman.elfaham@alexu.edu.eg](mailto:ayman.elfaham@alexu.edu.eg) (A.E.-F); [ambarakat@ksu.edu.sa](mailto:ambarakat@ksu.edu.sa) (A.B.).

1. **X-Ray structure determination**

The crystal of **5e** was immersed in cryo-oil, mounted in a loop, and measured at a temperature of 120 K. The X-ray diffraction data were collected on a Rigaku Oxford Diffraction Supernova diffractometer using Cu Kα radiation. The *CrysAlisPro* [1] software package was used for cell refinement and data reduction. A Multi-scan absorption correction (*CrysAlisPro*[1]) was applied to the intensities before the structure solution. The structure was solved by the intrinsic phasing (*SHELXT* [2]) method. Structural refinement was carried out using *SHELXL*[3] software with *SHELXLE*[4] graphical user interface. The NH and O(5)-H(5) hydrogen atoms were located from the difference Fourier map and refined isotropically. The O(6)-H(6A) hydrogen was located from the difference Fourier map but constrained to ride on its parent oxygen with U_iso_ = 1.5^.^U_eq_(parent atom). Other hydrogen atoms were positioned geometrically and constrained to ride on their parent atoms, with C-H = 0.95 -1.00 Å and U_iso_ = 1.2-1.5·U_eq_ (parent atom). The crystallographic details are summarized in Table S1.

**Table S1.** Crystal Data.

|  | **5e** |
| --- | --- |
| CCDC | 2293600 |
| empirical formula | C_25_H_24_ClN_3_O_6_S_2_ |
| fw | 562.04 |
| temp (K) | 120(2) |
| *λ*(Å) | 1.54184 |
| cryst syst | Monoclinic |
| space group | P2**_1_**/n |
| *a* (Å) | a = 11.5475(3) |
| *b* (Å) | b = 15.7550(4) |
| *c* (Å) | c = 14.6493(3) |
| β (deg) | 104.655(2) |
| *V* (Å^3^) | 2578.45(11) |
| Z | 4 |
| *ρ*_calc_ (Mg/m^3^) | 1.448 |
| *μ*(Mo K*α*) (mm^-1^) | 3.225 |
| No. reflns. | 20832 |
| Unique reflns. | 5413 |
| Completeness to θ=67.684° | 100% |
| GOOF (F^2^) | 1.099 |
| R_int_ | 0.0287 |
| R1^a^ (*I* ≥ 2σ) | 0.0487 |
| wR2^b^ (*I* ≥ 2σ) | 0.1291 |

*^a^* *R*_1_ = Σ||*F*_o_| – |*F*_c_||/Σ|*F*_o_|. *^b^wR*_2_ = {Σ[*w*(*F*_o_^2^ – *F*_c_^2^)^2^]/ Σ[*w*(*F*_o_^2^)^2^]}^1/2^

**2. Biology**

**2.1. Cytotoxicity**

Both breast cancer; MCF-7 and MDA-MB-231 and normal (WISH) cells were purchased from the National Research Institute, Egypt, and maintained in RPMI-1640 medium L-Glutamine (Lonza Verviers SPRL, Belgium, cat#12-604F). All cells were incubated at 37 ºC in 5% carbon dioxide atmosphere (NuAire). Cells were plated at a density of 5x10^4^ cells in triplicates in a plate of 96 wells. On the second day, cells were treated with the compounds with concentrations of (0.01, 0.1, 1, 10, and 100 µM). Cell viability was assessed using the MTT assay [5] [6].

**2.2. PIM-1 kinase Inhibitory Assay**

Compounds **5g, 5l, 5n,** Erlotinib and Roscovitine were evaluated for their EGFR (BPS Bioscience Corporation catalog#40321) and CDK-2 kinase (Catalog #79599, Kinase-Glo

Plus, Promega, USA) inhibition assay. They were dissolved in DMSO (0.1%), and four serial concentrations were prepared following Barakat *et al.* 2023 [7] and the manufacturer's instructions [8].

**2.3. Investigation of apoptosis**

**2.3.1. Annexin V/PI staining and cell cycle analysis**

MCF-7 cells were seeded into 6-well culture plates (3-5× 10^5^ cells/well) and incubated overnight. Cells were treated with compound **5g** at their IC_50_ values for 48 h. Next, media supernatants and cells were collected and rinsed with ice-cold PBS. Then cells were suspended the cells in 100 µL of annexin binding buffer solution "25 mM CaCl2, 1.4 M NaCl, and 0.1 M Hepes/NaOH, pH 7.4” and incubation with “Annexin V-FITC solution (1:100) and propidium iodide (PI)” at a concentration equals 10 µg/mL in the dark for 30 min. Stained cells were then acquired by BD FACSCalibur™ Flow Cytometer [9–11].

**2.3.3.2. Real time-polymerase chain reaction for the selected genes**

Gene expression of Bcl-2, the anti-apoptotic gene, and the pro-apoptotic genes P53, Bax, and Caspapses-3,8,9 were examined to delve deeper into the apoptotic pathway. MCF-7 cells were then treated with compound **5g** at their IC_50_ values for 48 h. After treatment, RT-PCR reaction was carried out following routine work. Then, the Ct values were collected to calculate the relative genes’ expression in all samples by normalization to the β-actin housekeeping gene [9,12]**.**

**Raw data of biology work**

- **Cytotoxicity against MCF-7 cancer cells**


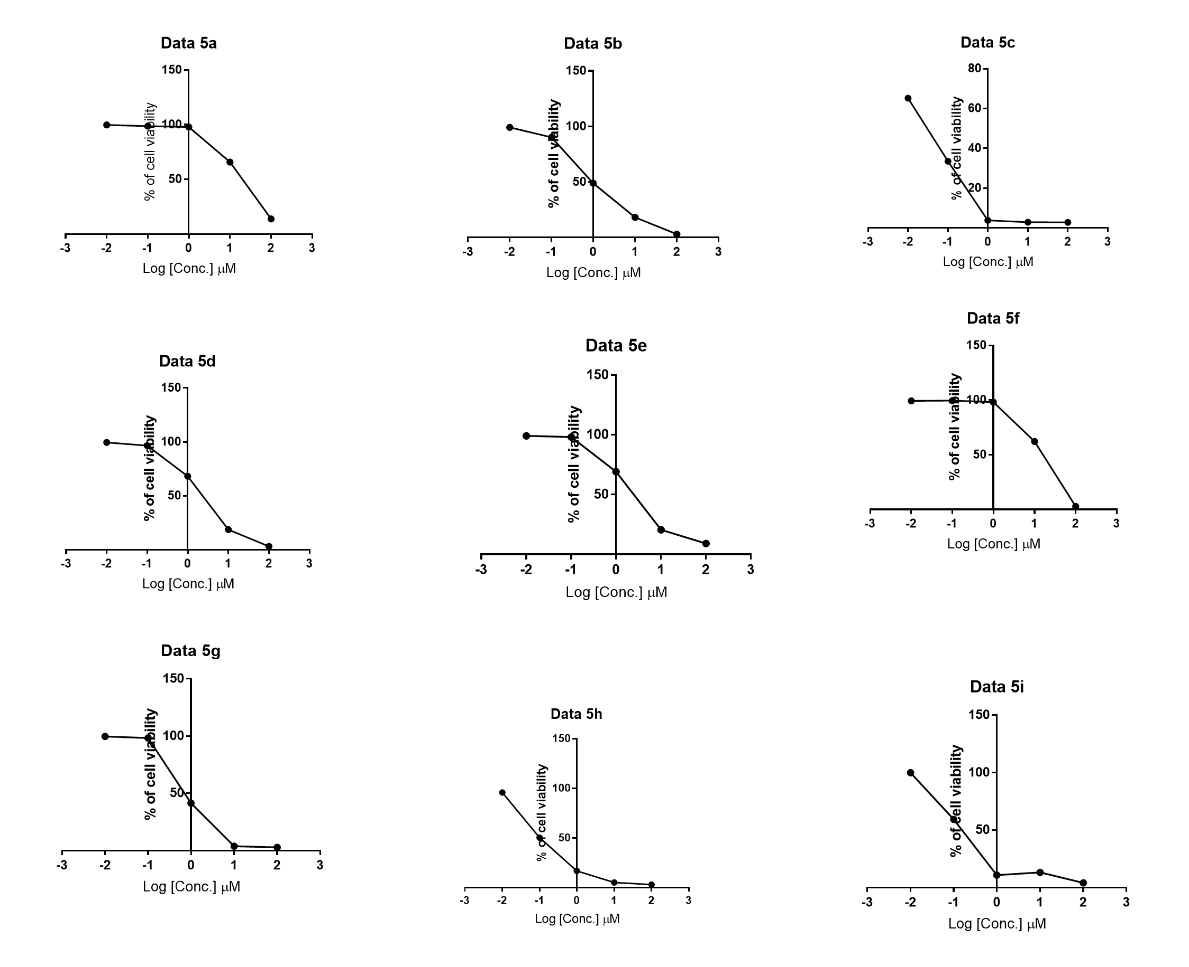


**
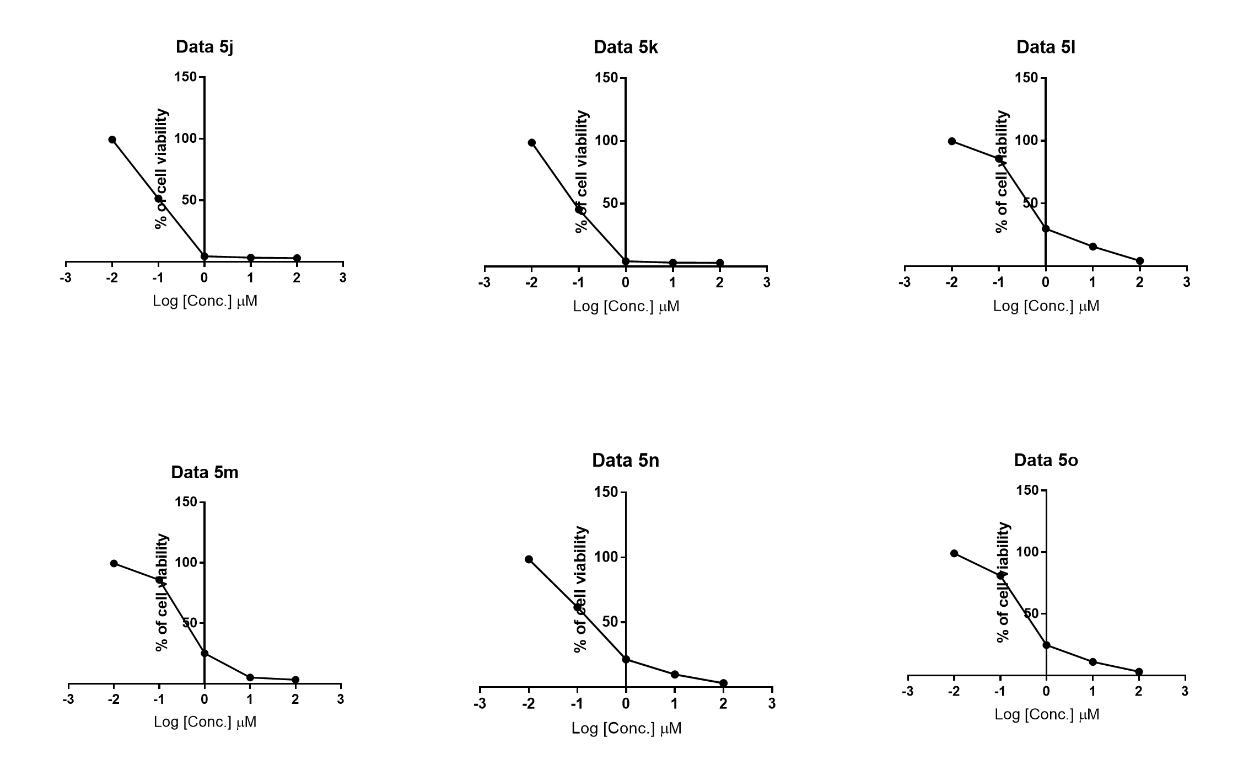
**

- **Cytotoxicity against MDA-MB-231 cancer cells**

**
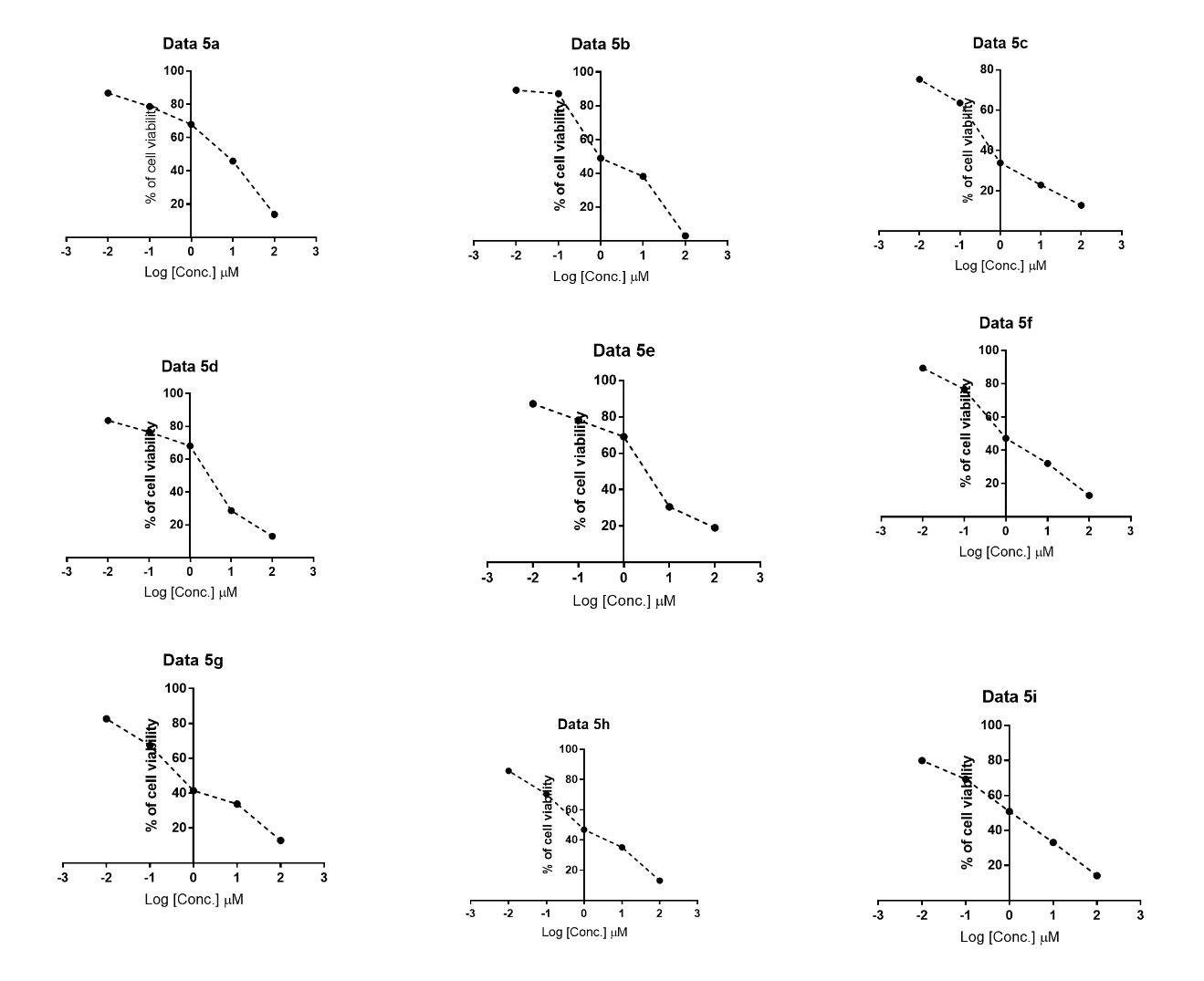
**

**
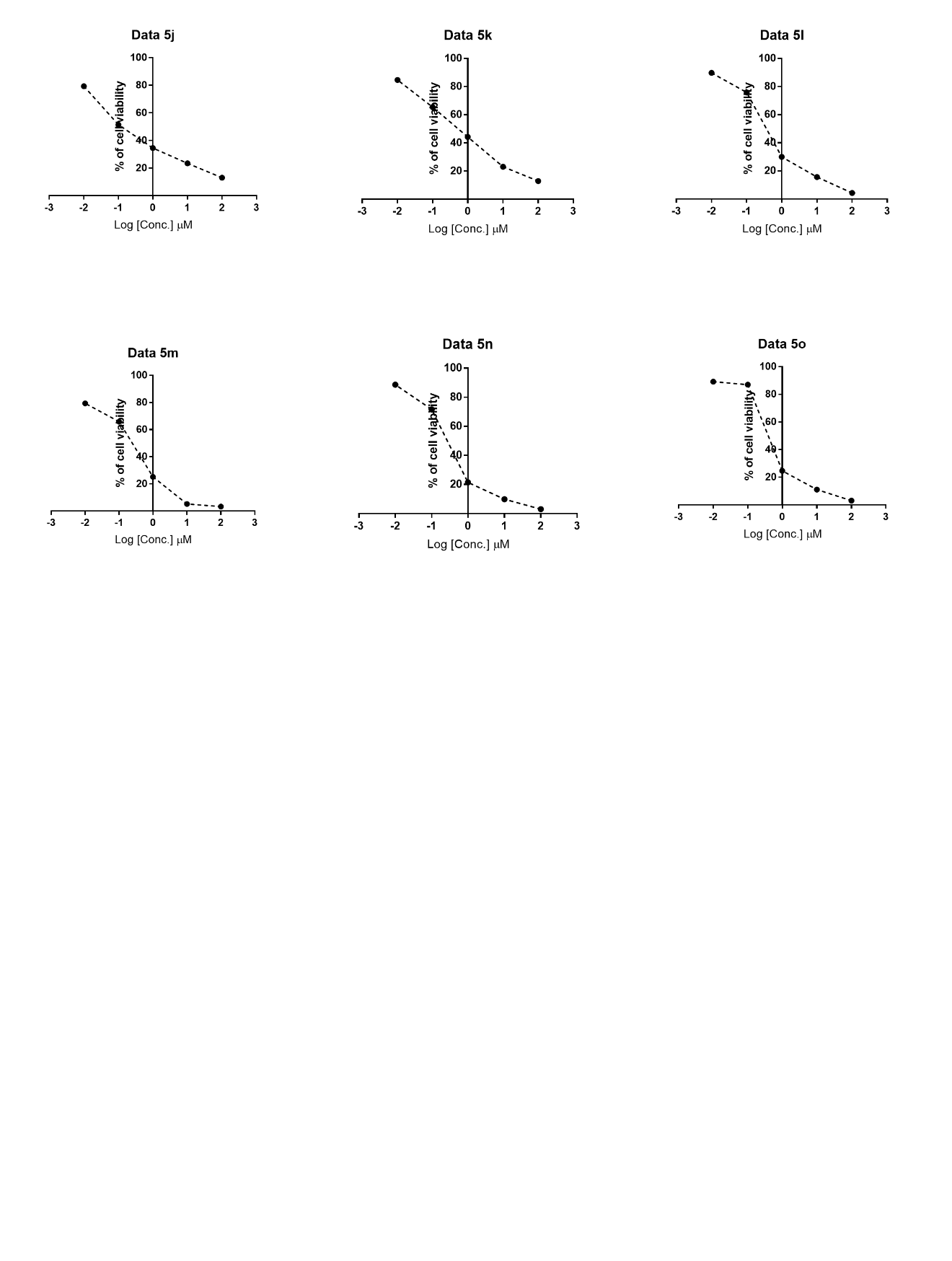
**

- **Enzyme targeting of the three compounds against CDK-2 and EGFR inhibition.**

1. **EGFR**

| **EGFR/CDk-2** |  |  |  |  |  |  |  |  | **Erlotinib=30 nM** |
| --- | --- | --- | --- | --- | --- | --- | --- | --- | --- |
| **ΔT=30 min** |  |  |  |  |  |  |  |  |  |
| **Compound [µM]** | **Cont.** | **10** | **5** | **2.5** | **1** | **0.1** | **0.01** |  |  |
| **5g** | 0.649 | 0.079 | 0.212 | 0.217 | 0.261 | 0.49 | 0.53 |  |  |
|  | 0.64 | 0.034 | 0.21 | 0.208 | 0.256 | 0.438 | 0.59 |  |  |
|  | 0.643 | 0.039 | 0.189 | 0.22 | 0.261 | 0.419 | 0.63 | **IC50= 260 nM±5.8** | |
| Mean | 0.644 | 0.050666667 | 0.203667 | 0.215 | 0.259333333 | 0.449 | 0.583333333 |  |  |
| SD | 0.004583 | 0.024664414 | 0.012741 | 0.006244998 | 0.002886751 | 0.036755952 | 0.05033223 |  |  |
| % of valibility | 100 | 7.867494824 | 31.62526 | 33.38509317 | 40.26915114 | 69.72049689 | 90.57971014 |  |  |
| % of inhibition | 0 | 92.60250518 | 68.37474 | 66.61490683 | 59.73084886 | 30.27950311 | 9.420289855 |  |  |
|  |  |  |  |  |  |  |  |  |  |
| **ΔT=30 min** |  |  |  |  |  |  |  |  |  |
| **Compound [µM]** | **Cont.** | **10** | **5** | **2.5** | **1** | **0.1** | **0.01** |  |  |
| **5l** | 0.649 | 0.079 | 0.128 | 0.259 | 0.301 | 0.414 | 0.429 |  |  |
|  | 0.64 | 0.072 | 0.127 | 0.279 | 0.33 | 0.419 | 0.439 |  |  |
|  | 0.643 | 0.076 | 0.129 | 0.259 | 0.313 | 0.329 | 0.479 | **IC50= 670 nM±10.04** | |
| Mean | 0.644 | 0.075666667 | 0.128 | 0.265666667 | 0.314666667 | 0.387333333 | 0.449 |  |  |
| SD | 0.004583 | 0.003511885 | 0.001 | 0.011547005 | 0.014571662 | 0.05057997 | 0.026457513 |  |  |
| % of valibility | 100 | 11.7494824 | 19.87578 | 41.25258799 | 48.86128364 | 60.14492754 | 69.72049689 |  |  |
| % of inhibition | 0 | 89.8505176 | 80.12422 | 58.74741201 | 51.13871636 | 39.85507246 | 30.27950311 |  |  |
|  |  |  |  |  |  |  |  |  |  |
|  |  |  |  |  |  |  |  |  |  |
|  |  |  |  |  |  |  |  |  |  |
| **ΔT=30 min** |  |  |  |  |  |  |  |  |  |
| **Compound [µM]** | **Cont.** | **10** | **5** | **2.5** | **1** | **0.1** | **0.01** |  |  |
| **5n** | 0.649 | 0.067 | 0.201 | 0.241 | 0.239 | 0.382 | 0.49 |  |  |
|  | 0.64 | 0.059 | 0.179 | 0.249 | 0.241 | 0.369 | 0.479 |  |  |
|  | 0.643 | 0.039 | 0.211 | 0.233 | 0.218 | 0.401 | 0.412 | **IC50= 400 nM±11.2** | |
| Mean | 0.644 | 0.055 | 0.197 | 0.241 | 0.232666667 | 0.384 | 0.460333333 |  |  |
| SD | 0.004583 | 0.014422205 | 0.016371 | 0.008 | 0.01274101 | 0.016093477 | 0.04221769 |  |  |
| % of valibility | 100 | 8.540372671 | 30.59006 | 37.42236025 | 36.12836439 | 59.62732919 | 71.48033126 |  |  |
| % of inhibition | 0 | 91.21962733 | 69.40994 | 62.57763975 | 63.87163561 | 40.37267081 | 28.51966874 |  |  |

**2. CDK2**

|  |  |  |  |  |  |  |  |  | **Roscovtine=30 nM** | |
| --- | --- | --- | --- | --- | --- | --- | --- | --- | --- | --- |
| **ΔT=30 min** |  |  |  |  |  |  |  |  |  |  |
| **Compound [µM]** | **Cont.** | **10** | **5** | **2.5** | **1** | **0.1** | **0.01** |  |  |  |
| **5g** | 0.651 | 0.069 | 0.211 | 0.398 | 0.408 | 0.491 | 0.599 |  |  |  |
|  | 0.642 | 0.072 | 0.211 | 0.389 | 0.421 | 0.491 | 0.549 |  |  |  |
|  | 0.628 | 0.082 | 0.231 | 0.379 | 0.411 | 0.526 | 0.583 | **IC50= 0.301 uM= 301 nM** | | |
| Mean | 0.640333 | 0.174333 | 0.217667 | 0.388667 | 0.413333 | 0.502667 | 0.577 |  |  |  |
| % of valibility | 100 | 27.2254 | 33.99271 | 60.69755 | 64.54971 | 78.50078 | 90.10932 |  |  |  |
| % of inhibition | 0 | 91.9 | 66.00729 | 39.30245 | 35.45029 | 21.49922 | 9.890682 |  |  |  |
|  |  |  |  |  |  |  |  |  |  |  |
|  |  |  |  |  |  |  |  |  |  |  |
| **ΔT=30 min** |  |  |  |  |  |  |  |  |  |  |
| **Compound [µM]** | **Cont.** | **10** | **5** | **2.5** | **1** | **0.1** | **0.01** |  |  |  |
| **5l** | 0.651 | 0.065 | 0.231 | 0.385 | 0.398 | 0.498 | 0.595 |  |  |  |
|  | 0.642 | 0.064 | 0.221 | 0.367 | 0.385 | 0.478 | 0.581 |  |  |  |
|  | 0.628 | 0.068 | 0.241 | 0.325 | 0.378 | 0.484 | 0.572 | **IC50= 0.345 uM= 345 nM** | | |
| Mean | 0.640333 | 0.187667 | 0.231 | 0.359 | 0.387 | 0.486667 | 0.582667 |  |  |  |
| % of valibility | 100 | 29.30765 | 36.07496 | 56.06455 | 60.43727 | 76.00208 | 90.99427 |  |  |  |
| % of inhibition | 0 | 89.4 | 63.92504 | 43.93545 | 39.56273 | 23.99792 | 9.005726 |  |  |  |
|  |  |  |  |  |  |  |  |  |  |  |
|  |  |  |  |  |  |  |  |  |  |  |
| **ΔT=30 min** |  |  |  |  |  |  |  |  |  |  |
| **Compound [µM]** | **Cont.** | **10** | **5** | **2.5** | **1** | **0.1** | **0.01** |  |  |  |
| **5n** | 0.651 | 0.075 | 0.302 | 0.395 | 0.401 | 0.472 | 0.615 |  |  |  |
|  | 0.642 | 0.084 | 0.302 | 0.396 | 0.412 | 0.482 | 0.618 |  |  |  |
|  | 0.628 | 0.078 | 0.304 | 0.396 | 0.402 | 0.476 | 0.516 | **IC50= 0.557 uM= 557 nM** | | |
| Mean | 0.640333 | 0.274333 | 0.302667 | 0.395667 | 0.405 | 0.476667 | 0.583 |  |  |  |
| % of valibility | 100 | 42.84227 | 47.26705 | 61.79073 | 63.24831 | 74.4404 | 91.04633 |  |  |  |
| % of inhibition | 0 | 88.7 | 52.73295 | 38.20927 | 36.75169 | 25.5596 | 8.95367 |  |  |  |

- **Flow cytometry using Annexin V/PI staining with cell cycle analysis.**

**
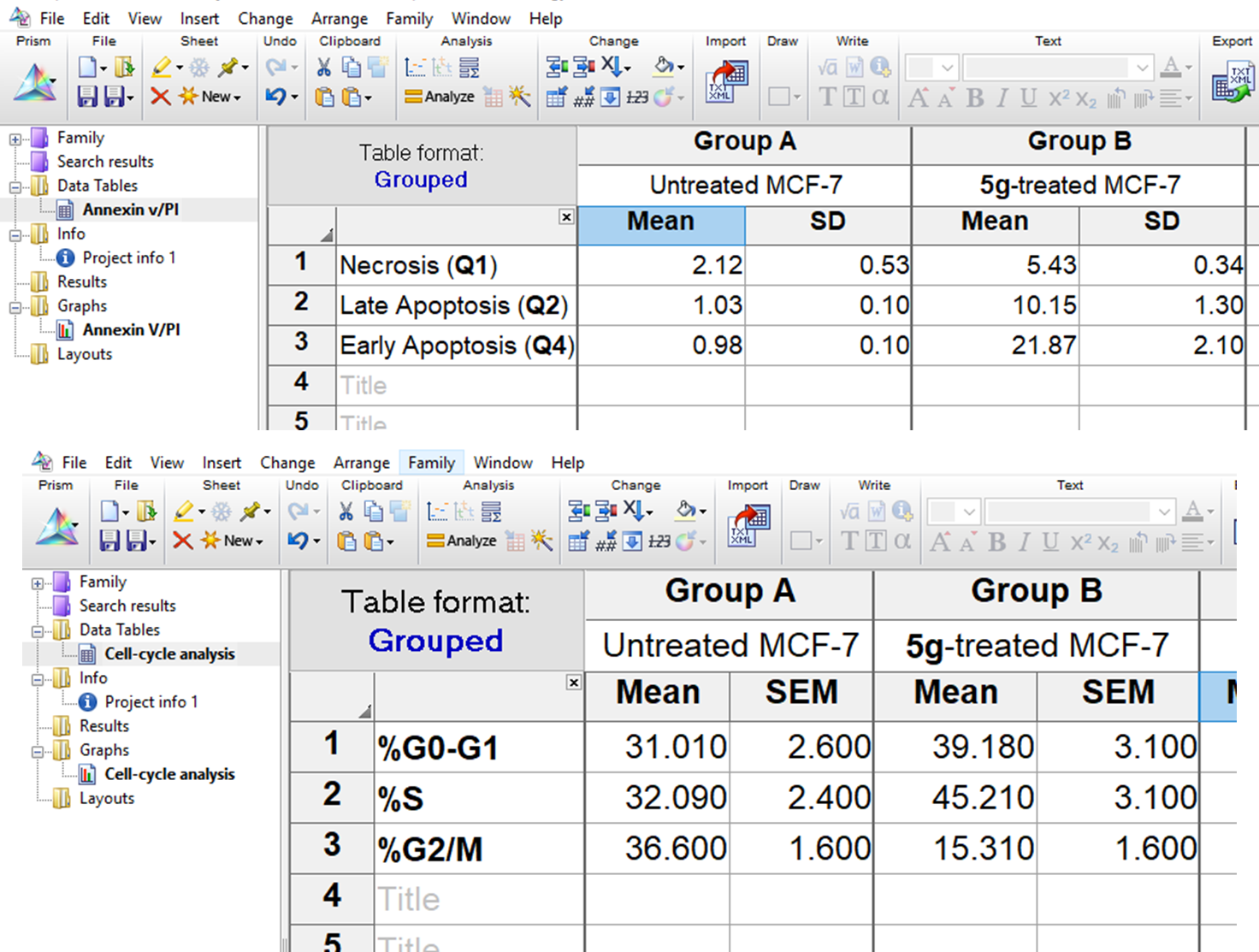
**

- **RT-PCR**


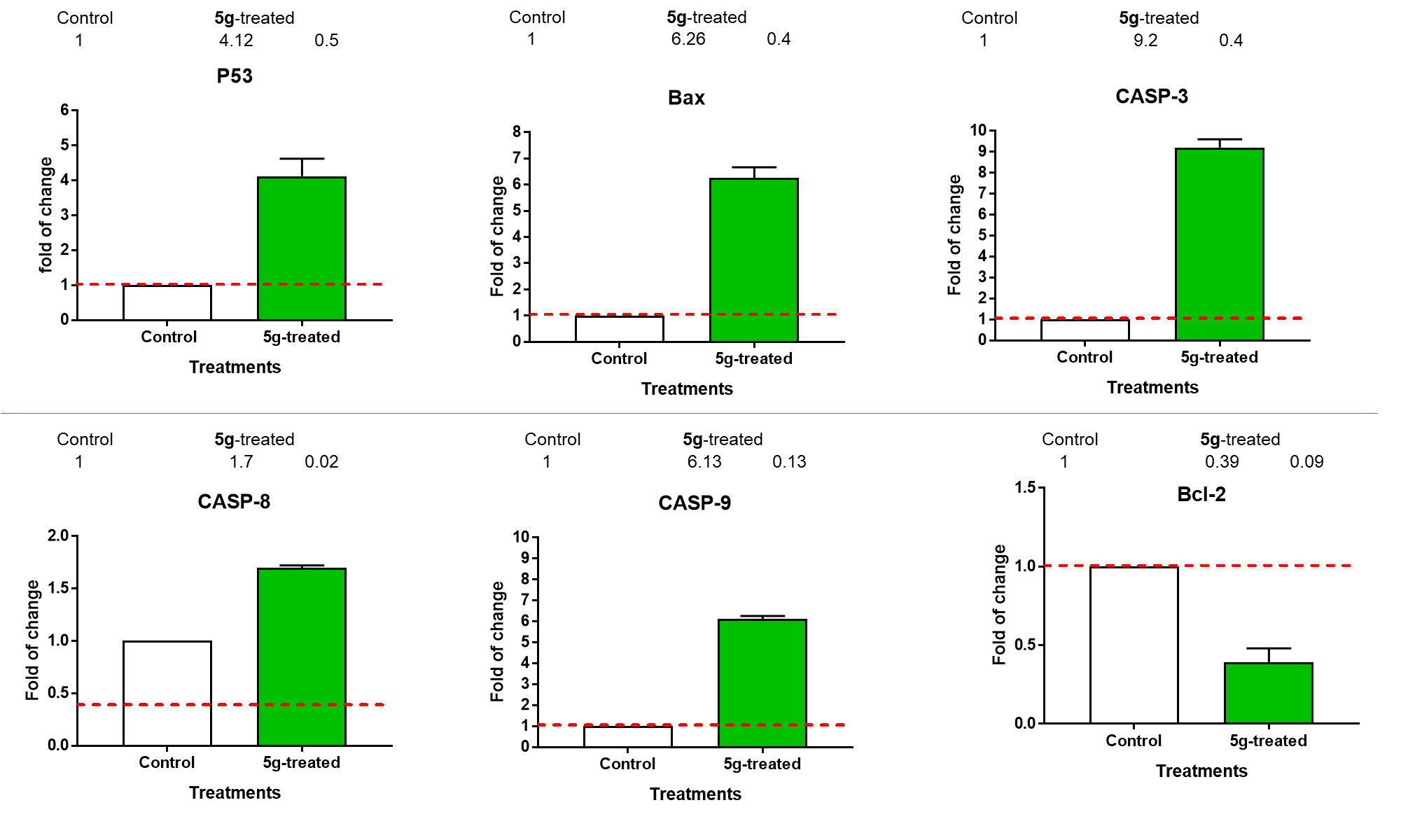


**3.Molecular docking protocol**

**Material and Methods:**

In order to understand binding mode and inhibitory mechanism of newly designed spirooxindole engrafted rhodanine analogue as anticancer agent, we conducted molecular docking experiments on two specific targets: CDK2 (PDB ID 6Q4G) [13] and EGFR (PDB ID 1M17) [14]. The target protein's crystal structures were obtained from the Protein Data Bank (www.rcsb.org/pdb). Both proteins were protonated, charged, and minimized using the MOE 2019.01 suite [15]. The chemical structures of active molecule **5g, 5l, 5n** and reference drugs roscovitine and erlotinib were produced utilizing the Builder tool integrated into MOE. The structures underwent additional preparation steps, such as atom-type corrections, protonation, minimization, and charge assignment. They were then saved in their respective 3D conformations. Before conducting the molecular docking experiments, a re-docking was carried out to evaluate the effectiveness of the software and docking technique. The results of the re-docking experiments revealed that MOE is a suitable software for conducting additional docking investigations on spirooxindole engrafted rhodanine derivatives.

In addition, a brief simulation of 100 nanoseconds was conducted using AMBER22 [16] to examine the dynamic stability of compound 5g within the active site of CDK2. The system was prepared according to the previously reported method, which involved the processes of minimization and equilibrium [17]. The trajectory analysis was conducted using the CPPTRAJ module, which is a part of the AMBER suite [18].

**References**

1. Rikagu Oxford Diffraction, *CrysAlisPro*, 2020, Rikagu Oxford Diffraction inc., Yarnton, Oxfordshire, England.
2. Sheldrick, G. M. *Acta Cryst.* **2015**, *A71*, 3-8.
3. Sheldrick, G. M. *Acta Cryst.* **2015**, *C71*, 3-8.
4. Hübschle, C. B.; Sheldrick, G. M.; Dittrich, B. *J. Appl. Cryst.* **2011**, *44*, 1281-1284.
5. T. Mosmann, Rapid colorimetric assay for cellular growth and survival: Application to proliferation and cytotoxicity assays, J. Immunol. Methods 65 (1983) 55–63. https://doi.org/10.1016/0022-1759(83)90303-4.
6. E.S. Tantawy, A.M. Amer, E.K. Mohamed, M.M. Abd Alla, M.S. Nafie, Synthesis, characterization of some pyrazine derivatives as anti-cancer agents: In vitro and in Silico approaches, J. Mol. Struct. 1210 (2020) 128013. https://doi.org/10.1016/j.molstruc.2020.128013.
7. A. Barakat, S. Alshahrani, A.M. Al-Majid, A.S. Alamary, M. Haukka, M.M. Abu-Serie, L.R. Domingo, S. Ashraf, Z. Ul-Haq, M.S. Nafie, M. Teleb, New spiro-indeno[1,2-b]quinoxalines clubbed with benzimidazole scaffold as CDK2 inhibitors for halting non-small cell lung cancer; stereoselective synthesis, molecular dynamics and structural insights, J. Enzyme Inhib. Med. Chem. 38 (2023) 2281260. https://doi.org/10.1080/14756366.2023.2281260.
8. CST - HTScan® Pim-1 Kinase Assay Kit, (n.d.). www.cellsignal.com/products/cellular-assay-kits/htscan-pim-1-kinase-assay-kit/7573 (accessed July 17, 2020).
9. M.S. Nafie, K. Arafa, N.K. Sedky, A.A. Alakhdar, R.K. Arafa, Triaryl dicationic DNA minor-groove binders with antioxidant activity display cytotoxicity and induce apoptosis in breast cancer, Chem. Biol. Interact. 324 (2020) 109087. https://doi.org/10.1016/j.cbi.2020.109087.
10. M.S. Nafie, A.M. Amer, A.K. Mohamed, E.S. Tantawy, Discovery of novel pyrazolo[3,4-b]pyridine scaffold-based derivatives as potential PIM-1 kinase inhibitors in breast cancer MCF-7 cells, Bioorg. Med. Chem. 28 (2020) 115828. https://doi.org/10.1016/j.bmc.2020.115828.
11. E.M. Gad, M.S. Nafie, E.H. Eltamany, M.S.A.G. Hammad, A. Barakat, A.T.A. Boraei, Discovery of New Apoptosis-Inducing Agents for Breast Cancer Based on Ethyl 2-Amino-4,5,6,7-Tetra Hydrobenzo[b]Thiophene-3-Carboxylate: Synthesis, In Vitro, and In Vivo Activity Evaluation, Molecules 25 (2020) 2523. https://doi.org/10.3390/molecules25112523.
12. M.S. Nafie, S. Mahgoub, A.M. Amer, Antimicrobial and antiproliferative activities of novel synthesized 6-(quinolin-2-ylthio) pyridine derivatives with molecular docking study as multi-targeted JAK2/STAT3 inhibitors, Chem. Biol. Drug Des. n/a (n.d.). https://doi.org/10.1111/cbdd.13791.
13. Wood DJ, Lopez-Fernandez JD, Knight LE, Al-Khawaldeh I, Gai C, Lin S, Martin MP, Miller DC, Cano C, Endicott JA, Hardcastle IR. FragLites—minimal, halogenated fragments displaying pharmacophore doublets. An efficient approach to druggability assessment and hit generation. Journal of Medicinal Chemistry. 2019 Mar 12;62(7):3741-52.
14. Stamos J, Sliwkowski MX, Eigenbrot C. Structure of the epidermal growth factor receptor kinase domain alone and in complex with a 4-anilinoquinazoline inhibitor. Journal of biological chemistry. 2002 Nov 29;277(48):46265-72.
15. “Molecular Operating Environment (MOE), 2013.08; Chemical Computing Group ULC, 1010 Sherbooke St. West, Suite #910, Montreal, QC, Canada, H3A 2R7, 2019.” 2019.
16. D.A. Case, H.M. Aktulga, K. Belfon, I.Y. Ben-Shalom, J.T. Berryman, S.R. Brozell, D.S. Cerutti, T.E. Cheatham, III, G.A. Cisneros, V.W.D. Cruzeiro, T.A. Darden, N. Forouzesh, G. Giambaşu, T. Giese, M.K. Gilson, H. Gohlke, A.W. Goetz, J. Harris, S. Izadi, S.A. Izmailov, K. Kasavajhala, M.C. Kaymak, E. King, A. Kovalenko, T. Kurtzman, T.S. Lee, P. Li, C. Lin, J. Liu, T. Luchko, R. Luo, M. Machado, V. Man, M. Manathunga, K.M. Merz, Y. Miao, O. Mikhailovskii, G. Monard, H. Nguyen, K.A. O’Hearn, A. Onufriev, F. Pan, S. Pantano, R. Qi, A. Rahnamoun, D.R. Roe, A. Roitberg, C. Sagui, S. Schott-Verdugo, A. Shajan, J. Shen, C.L. Simmerling, N.R. Skrynnikov, J. Smith, J. Swails, R.C. Walker, J. Wang, J. Wang, H. Wei, X. Wu, Y. Wu, Y. Xiong, Y. Xue, D.M. York, S. Zhao, Q. Zhu, and P.A. Kollman (2023), Amber 2023, University of California, San Francisco.
17. Khalil R, Ashraf S, Khalid A, Ul-Haq Z. Exploring Novel N-Myristoyltransferase Inhibitors: A Molecular Dynamics Simulation Approach. ACS omega. 2019 Aug 15;4(9):13658-70.
18. Roe DR, Cheatham III TE. PTRAJ and CPPTRAJ: software for processing and analysis of molecular dynamics trajectory data. Journal of chemical theory and computation. 2013 Jul 9;9(7):3084-95.


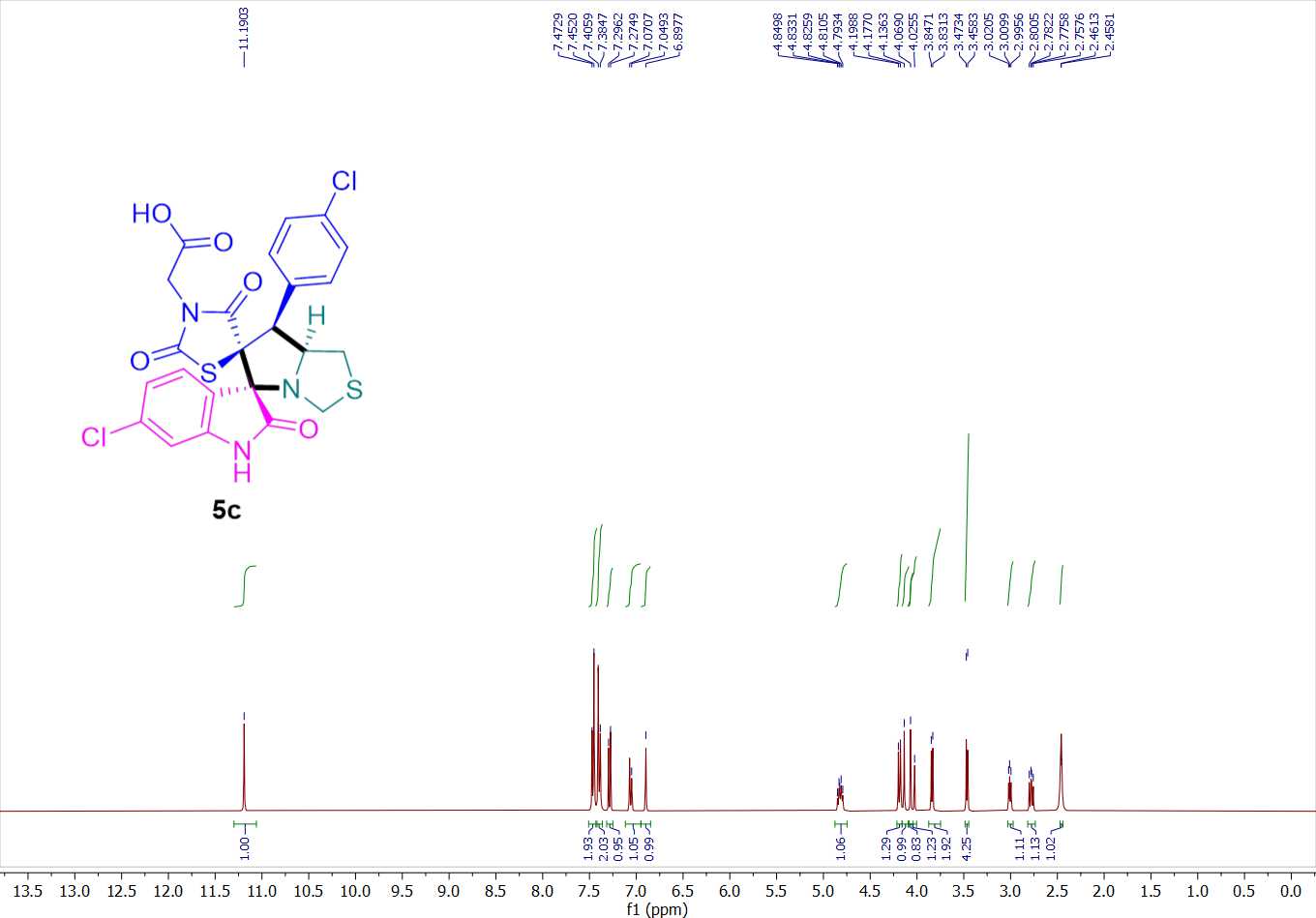


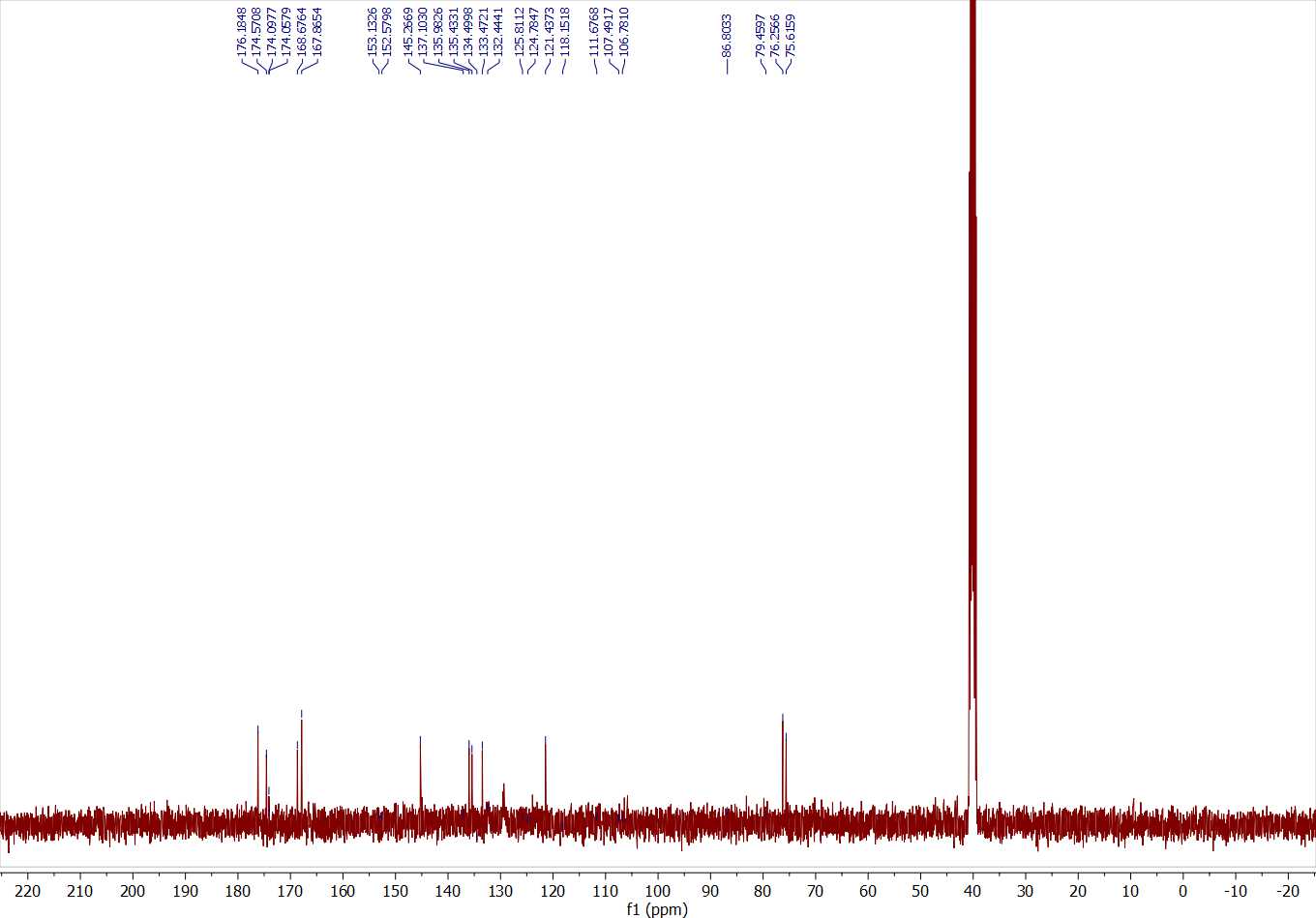


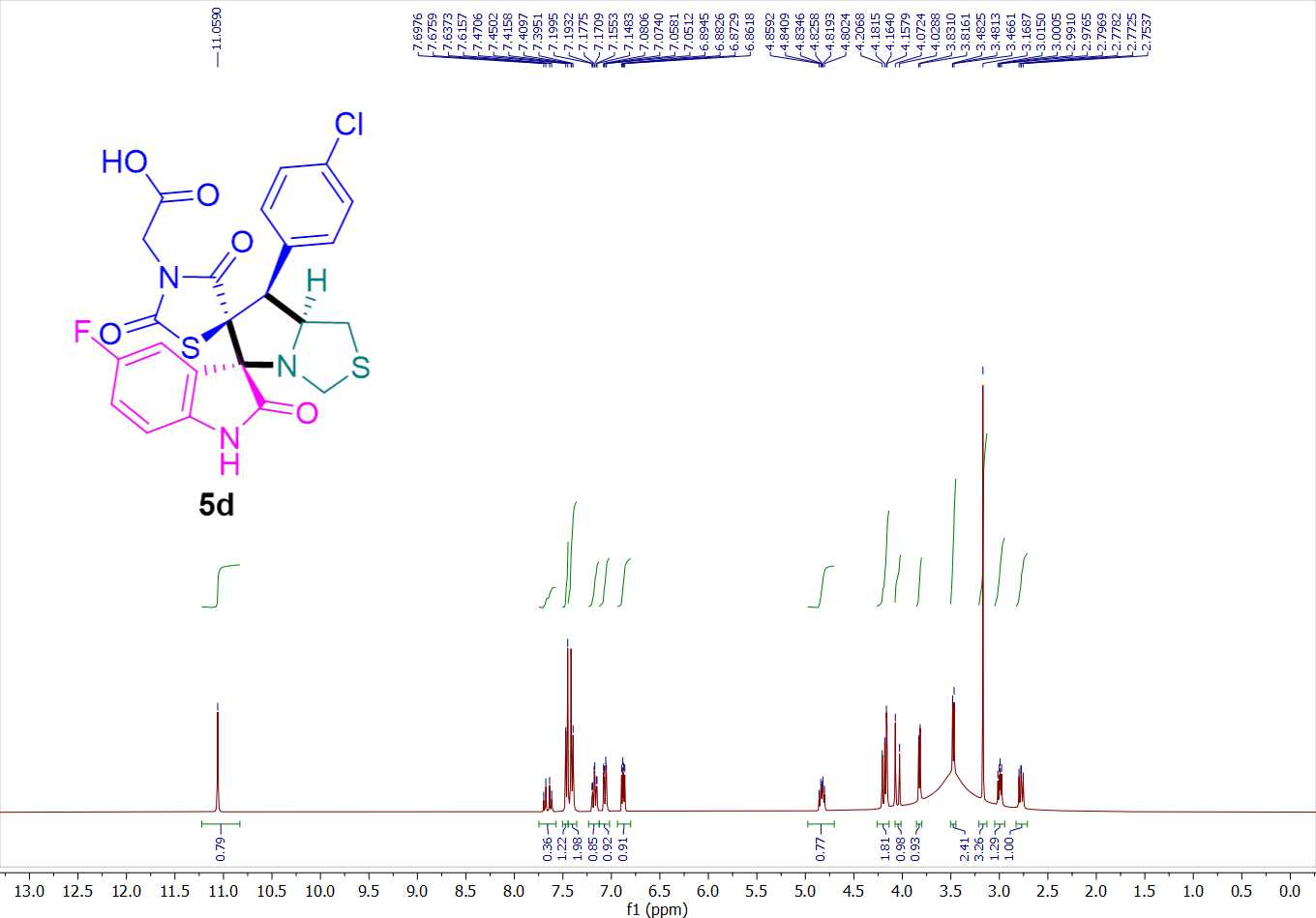


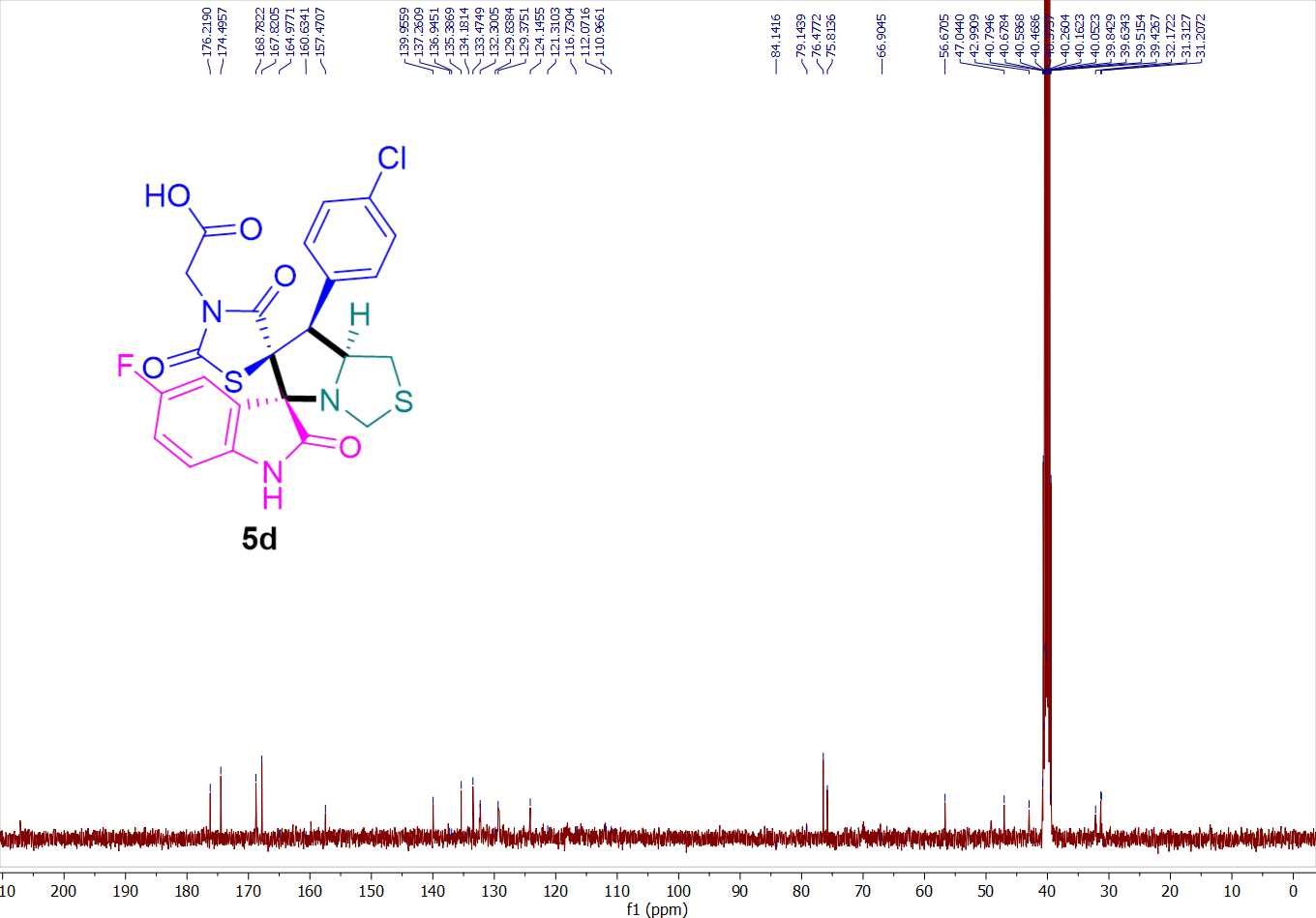


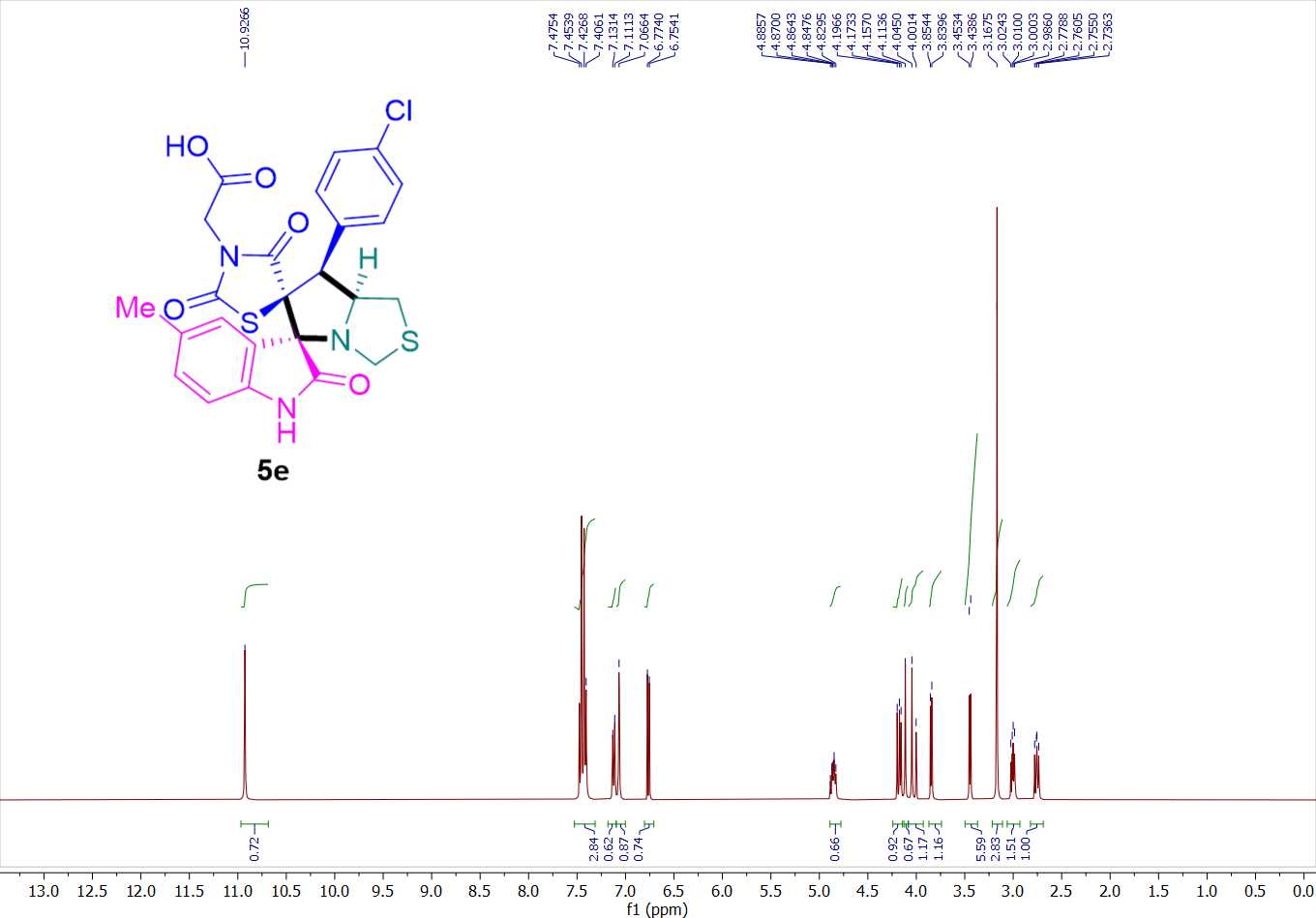


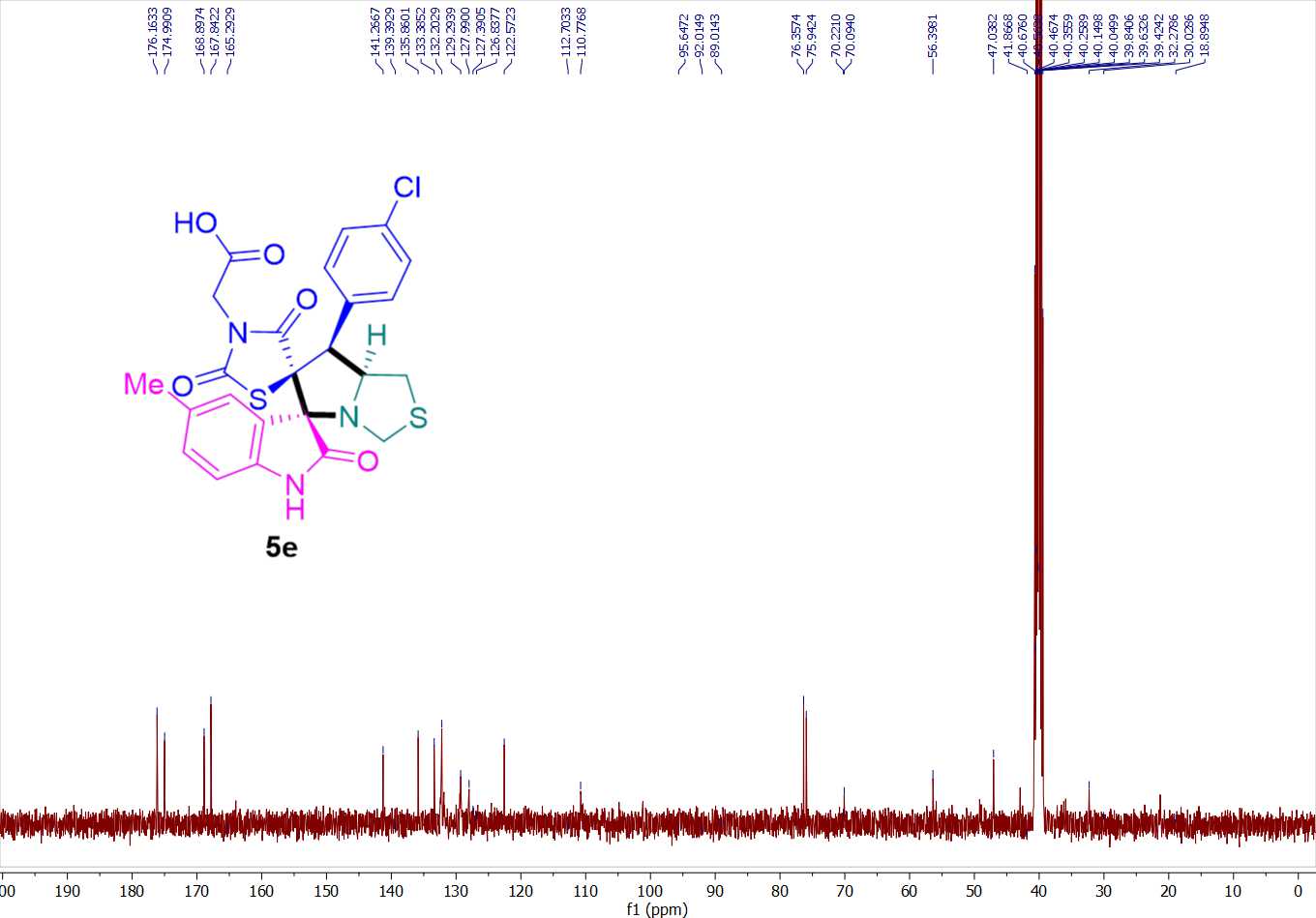


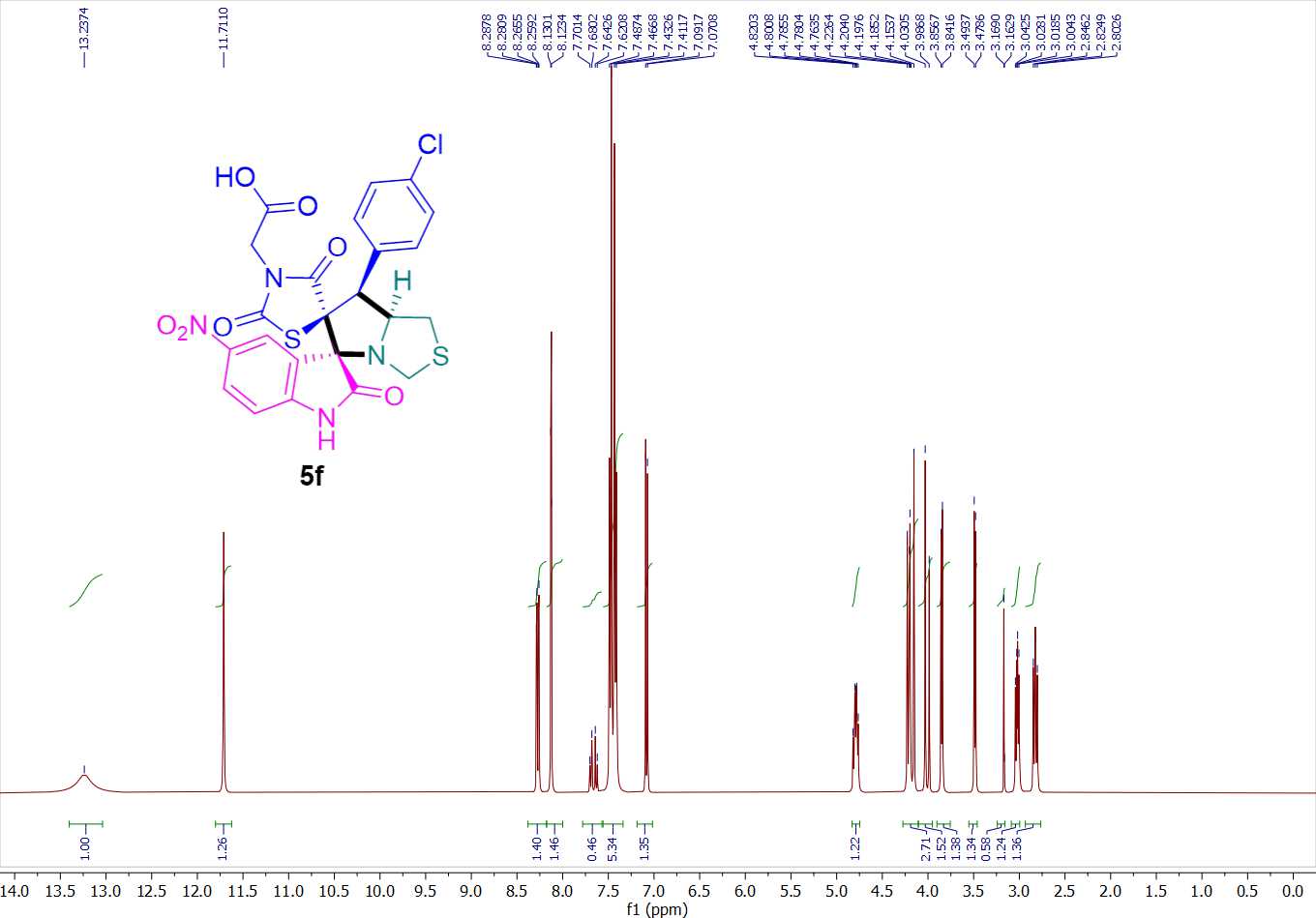


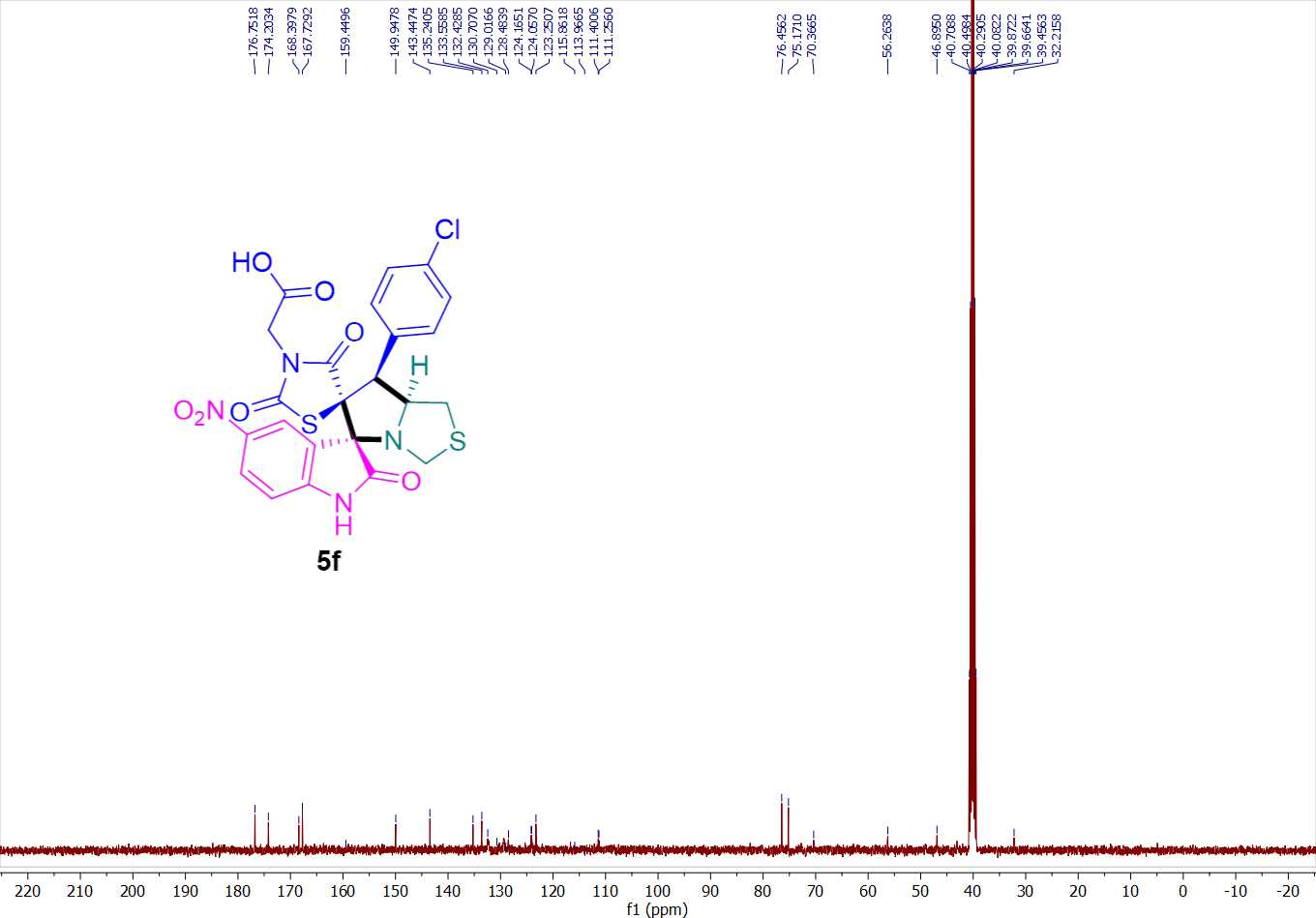


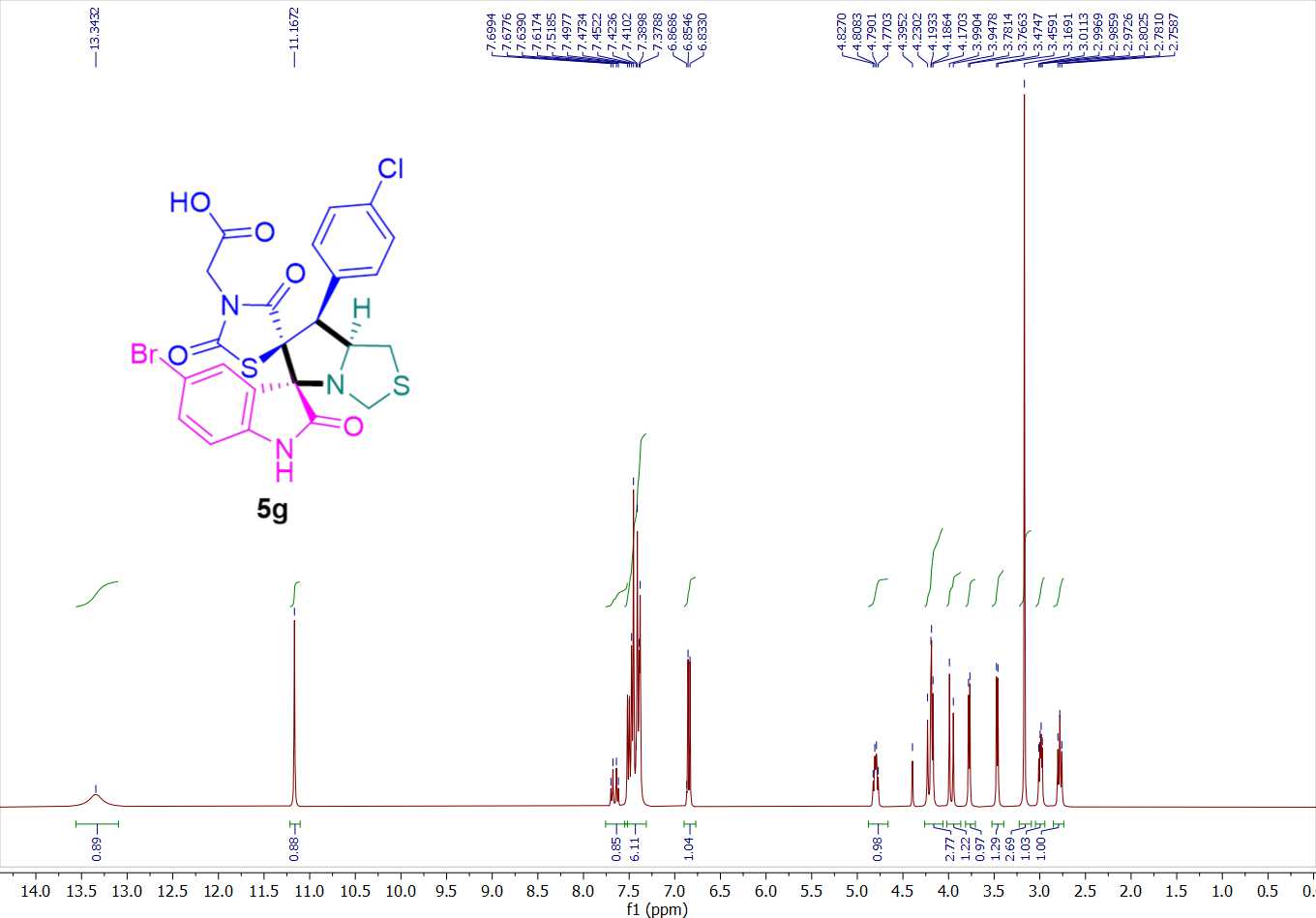


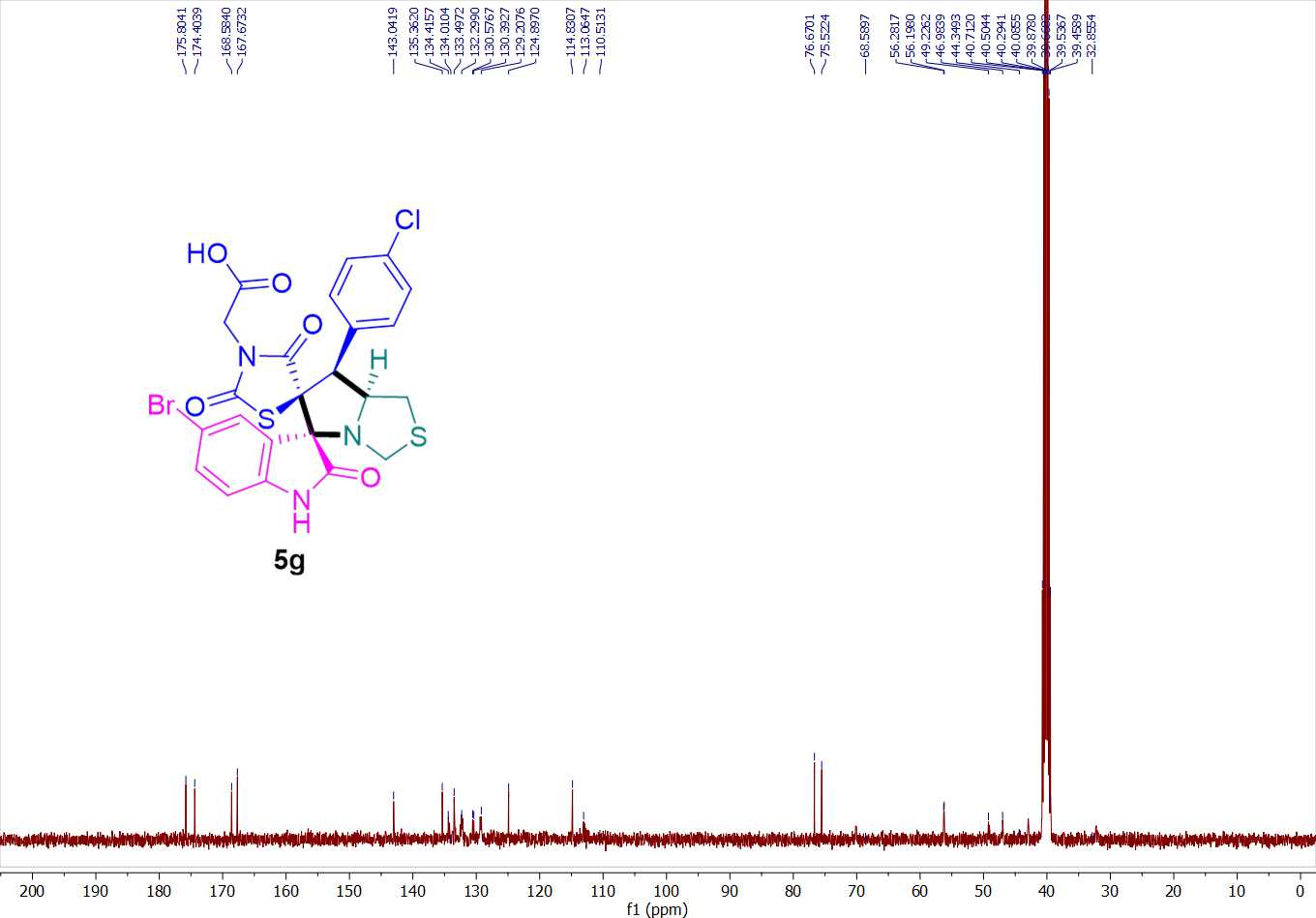


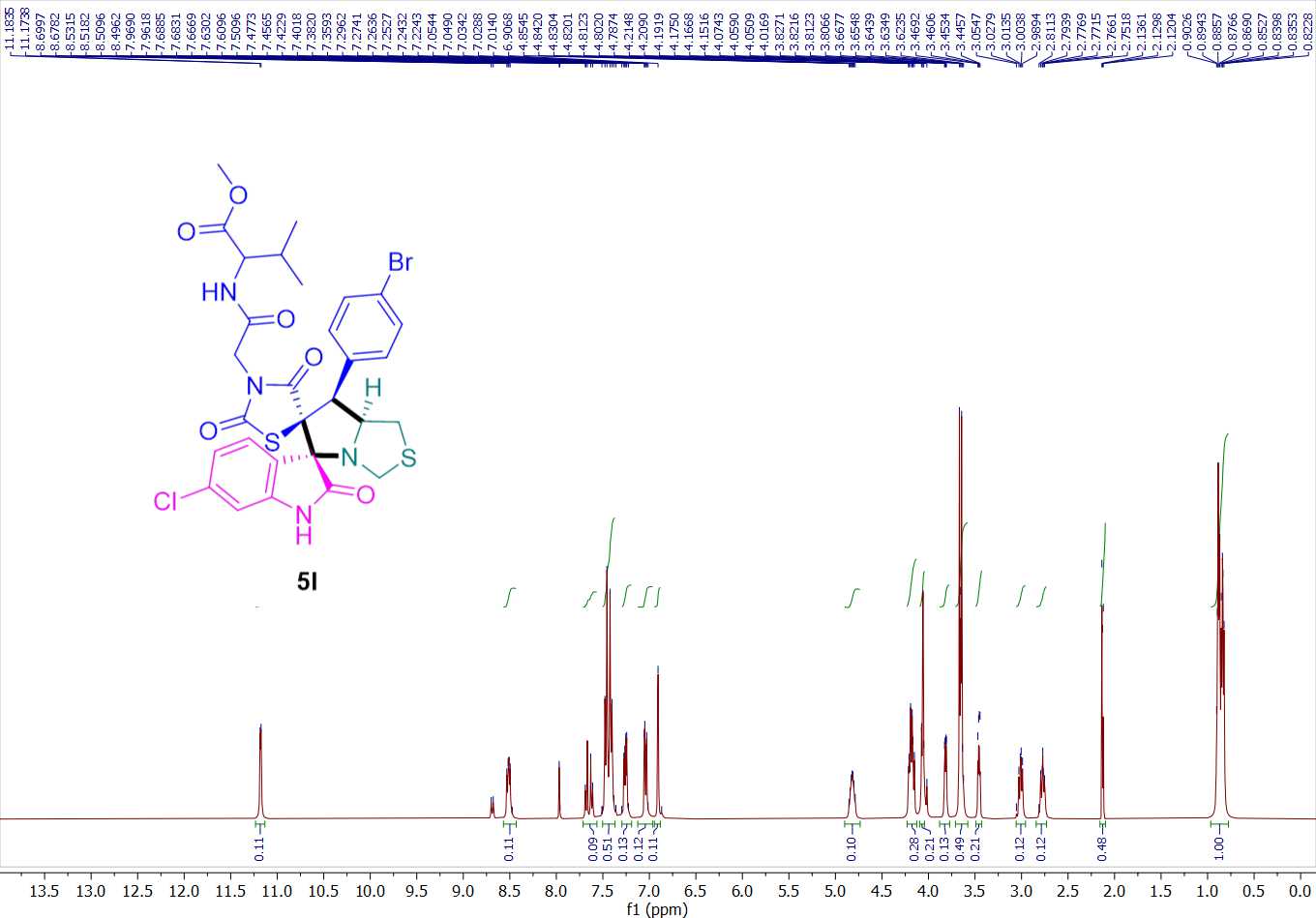


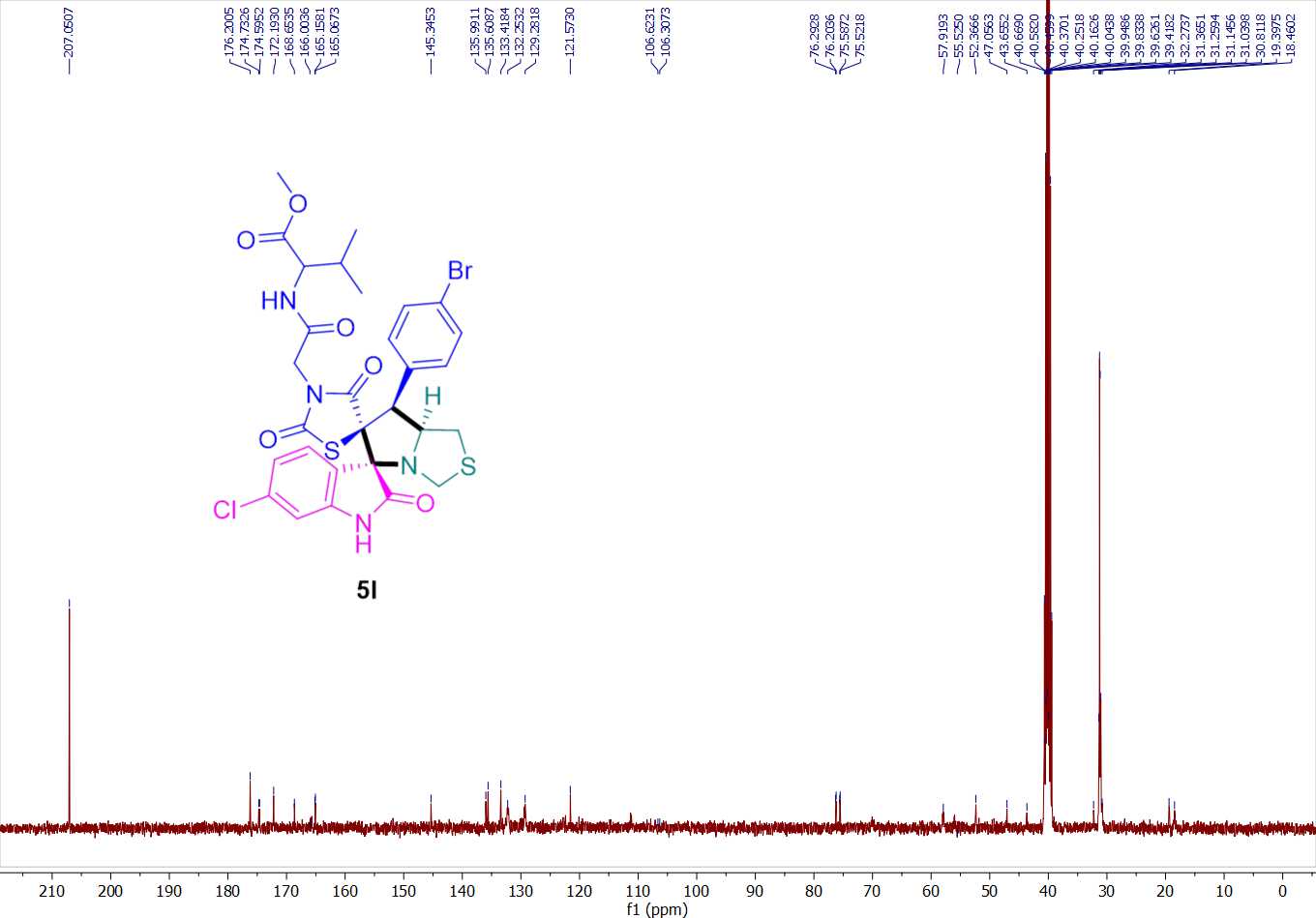


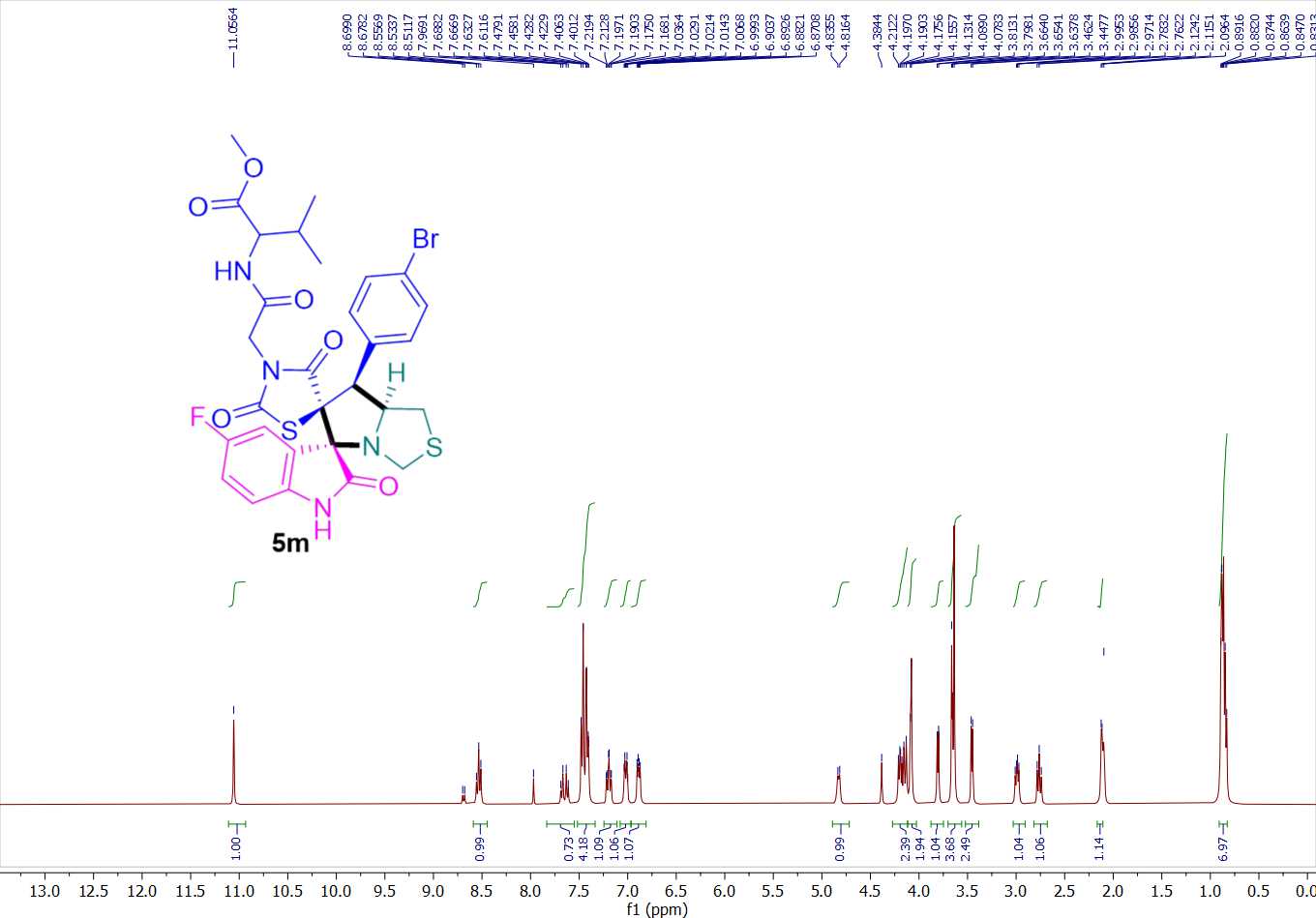


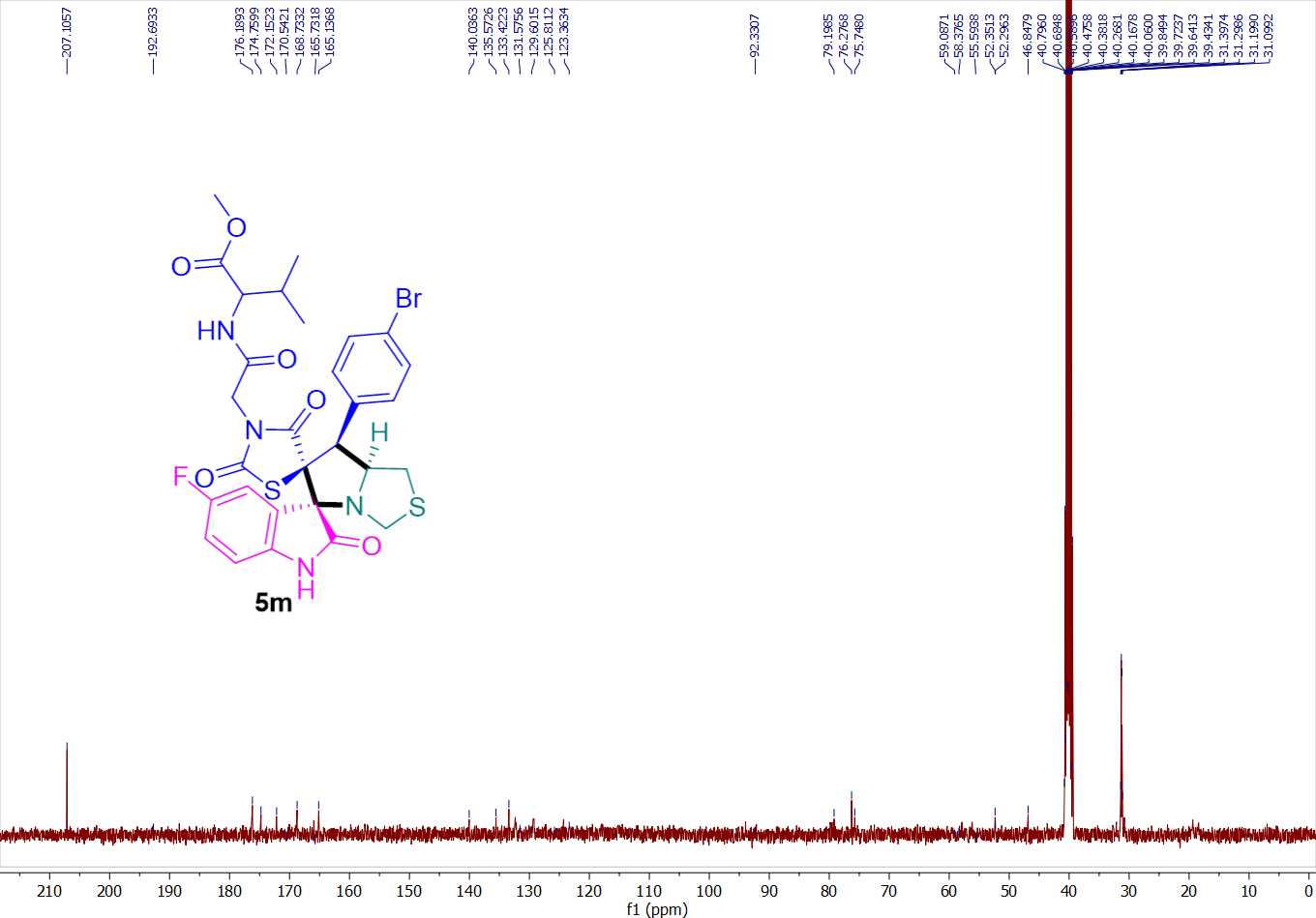


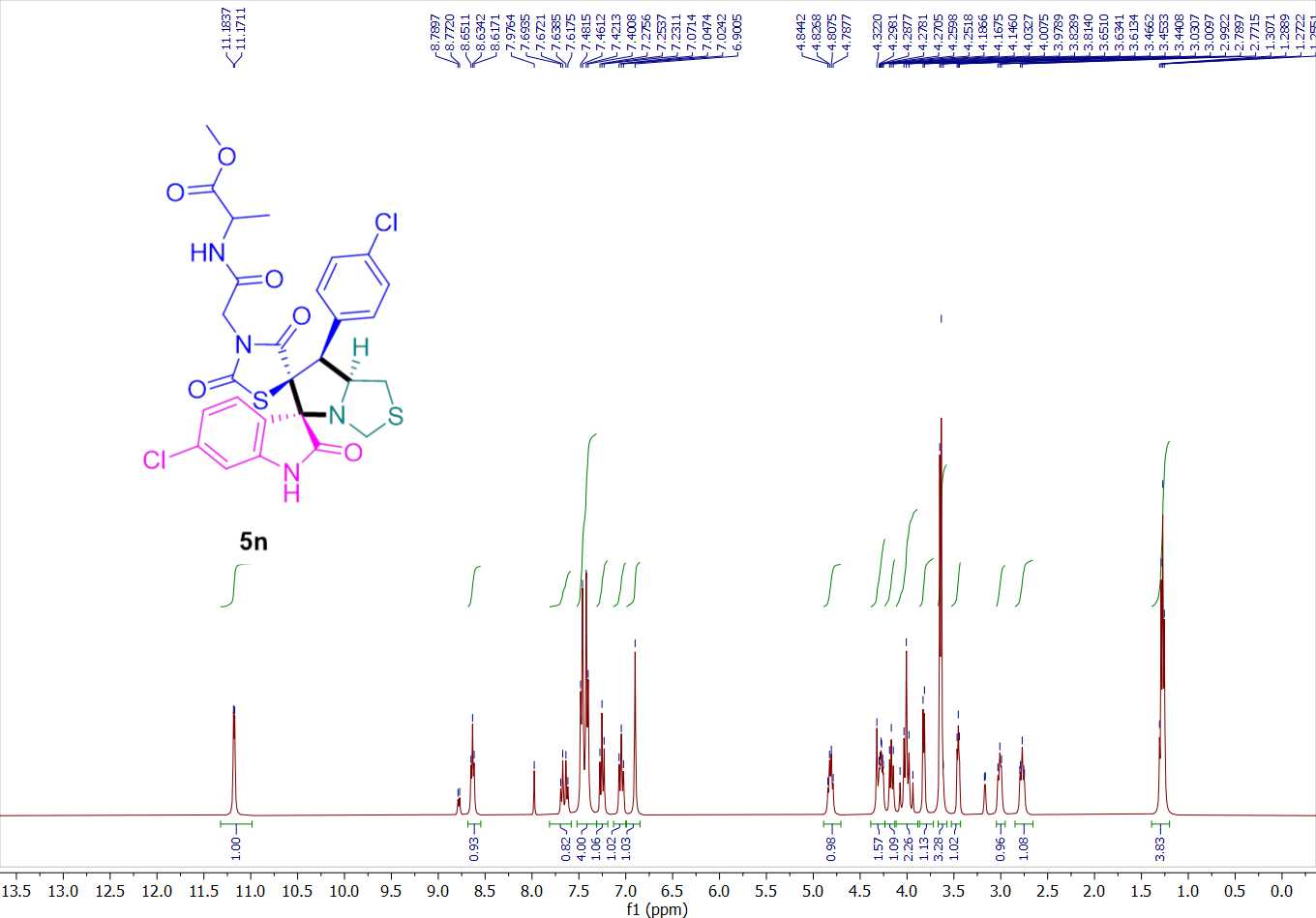


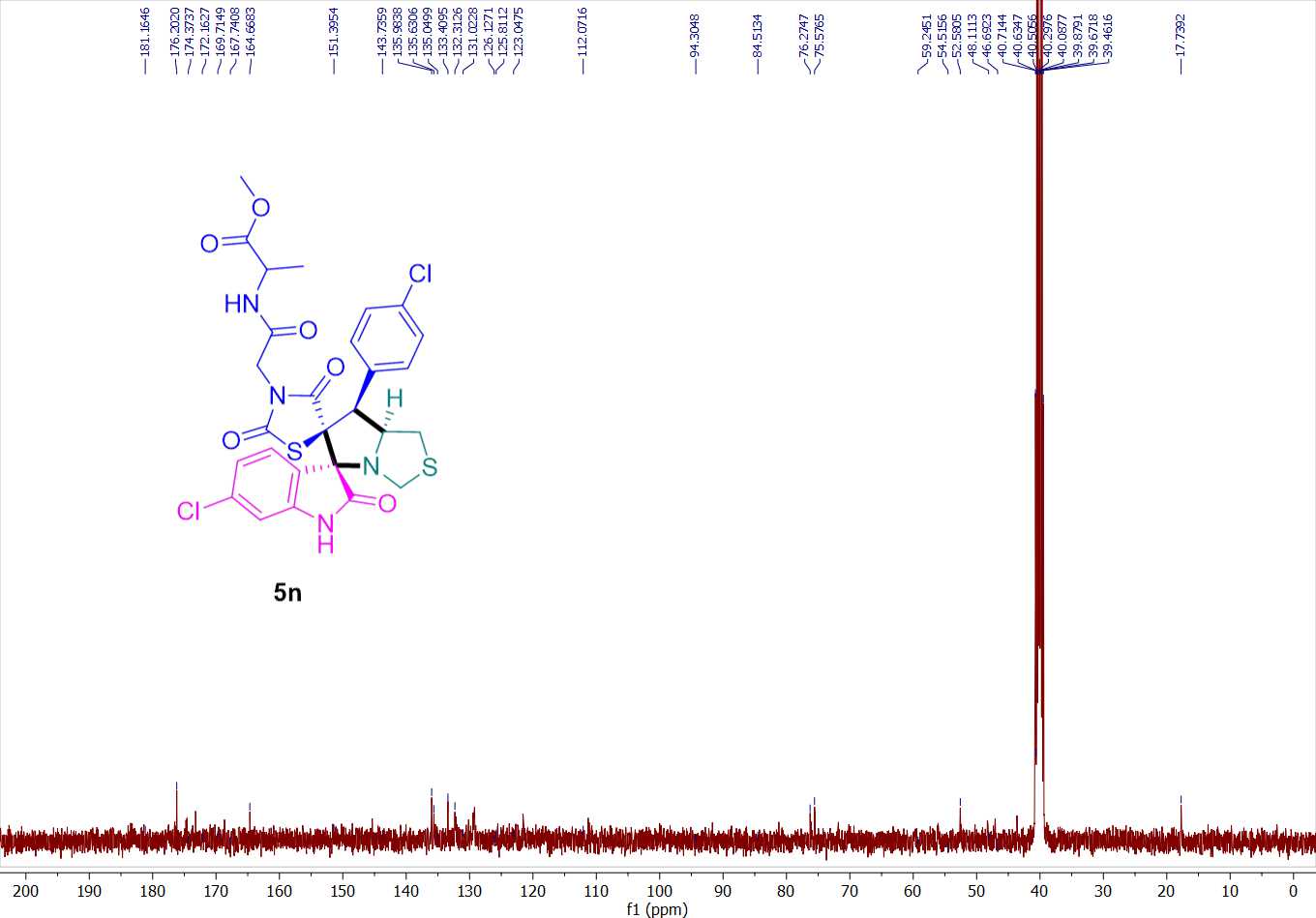


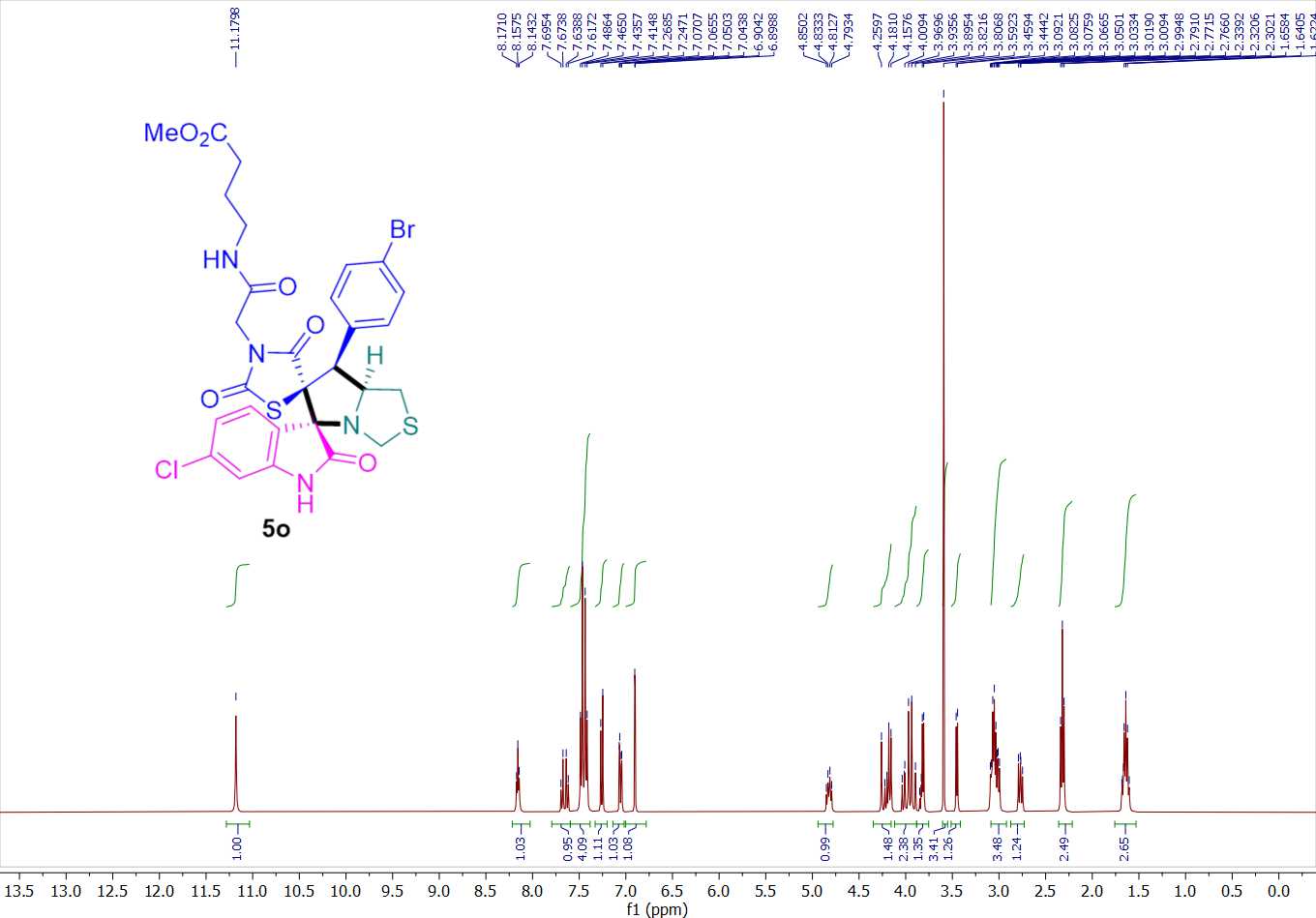


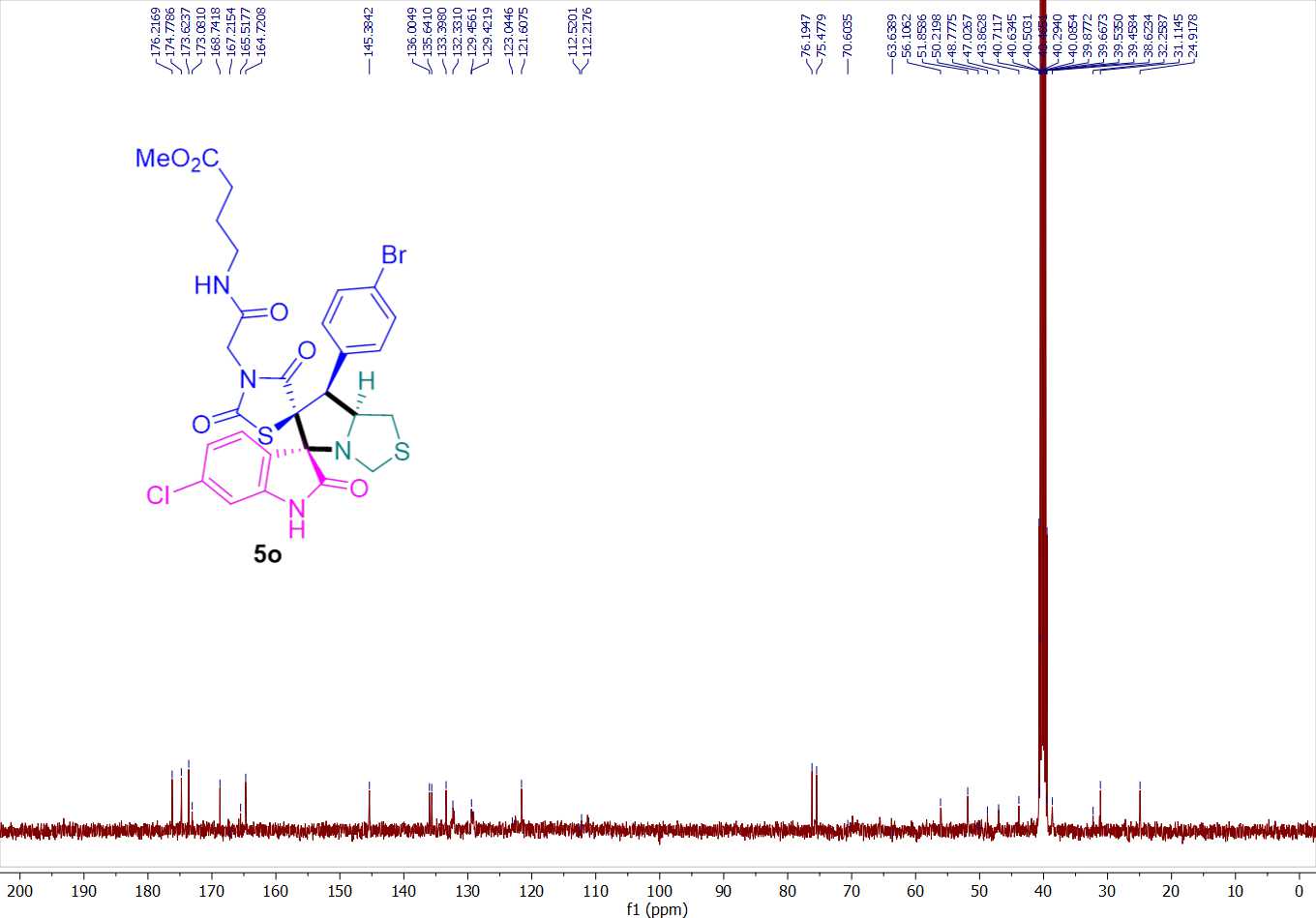

Supplement: Supplementary file 2 [file DataSheet2.docx]
